# A Hierarchical and Multiscale Framework for Characterizing Mouse Sleep–Wake Dynamics from 14-Day Continuous EEG: Validation of Age- and Sex-Dependent Remodeling

**DOI:** 10.3390/cells15121075

**Published:** 2026-06-13

**Authors:** Andrey Kostin, Anton Saevskiy, Md Aftab Alam, Yiqun Jiang, Natalia Suntsova, Md Noor Alam

**Affiliations:** 1 Research Service (151A3), Veterans Affairs Greater Los Angeles Healthcare System, Sepulveda, Los Angeles, CA 91343, USA; aftabalam@ucla.edu; 2 Scientific Research and Technology Center for Neurotechnology, Southern Federal University, 344006 Rostov-on-Don, Russia; saevskiy@sfedu.ru; 3 Department of Medicine, David Geffen School of Medicine, University of California, Los Angeles, CA 90095, USA; yiqunjiang@mednet.ucla.edu (Y.J.); suntsova@ucla.edu (N.S.)

**Keywords:** aging, NREM, sex differences, EEG, vigilance states, theta-dominant wake, power spectra, circadian regulation, multi-day recording, sleep–wake architecture

## Abstract

**Highlights:**

**What are the main findings?**

- Aging causes a dark-phase-specific 17–18% loss of theta-dominant active wake (TDW) in both male and female C57BL/6J mice, with reciprocal increases in quiet wake (nTDW) and NREM sleep, indicating circadian-phase-selective sleep–wake instability.
- A recurring N-shaped motif at the dark-to-light transition—a late-dark NREM rise, a pre-lights-on trough, and an early-light NREM peak—identifies a circadian window in which age-related sleep–wake instability and several sex-associated differences are most apparent.

**What are the implications of the main findings?**

- Integrating multi-day, multiscale vigilance-state analyses across circadian, ultradian, and spectral domains identifies sensitive candidate EEG-derived endpoints of sleep–wake aging that are often missed by short, male-only, single-metric study designs.
- Because cohort size contributes far more to statistical power than extending recording duration, sample expansion should be the primary design guidance for translational sleep-aging studies in inflammaging, neurodegeneration, and dementia.

**Abstract:**

Aging disrupts sleep, but how these changes are structured across circadian time, vigilance states, and sex remains poorly understood, because most prior studies used single-sex cohorts and few days of recordings. We continuously recorded 14 days of EEG/EMG in 24 C57BL/6J mice using a balanced 2 × 2 design (young vs. old; male vs. female; *n* = 6/group). A comprehensive multiscale analysis of the extended dataset enabled detailed reconstruction of 24 h sleep–wake architecture, better characterization of natural day-to-day variability including across multiple estrous cycles, and detection of rare bouts and transition events. Across seven levels of analysis, from circadian profiles to EEG spectral parameterization, the strongest aging effect was a dark-phase-specific 17–18% loss of theta-dominant active wake (TDW) in both sexes, with reciprocal increases in quiet wake (nTDW) and NREM sleep. We also identified a recurring N-shaped structural motif at the dark-to-light transition, where age-related and several sex-associated differences were most apparent. Broadly, old mice exhibited (i) shorter TDW bouts; (ii) a shift in NREM exit kinetics toward wakefulness; (iii) more brief and poorly consolidated “out-block” NREM episodes; and (iv) a slowing of waking theta and higher low-frequency TDW power. Variance decomposition indicated that statistical power depends more on sample size than on recording length. Together, aging reflects a coordinated, circadian-phase-specific reorganization of sleep–wake architecture. Sex-related and interaction findings should be interpreted as hypothesis-generating pending larger cohorts.

## 1. Introduction

Sleep and aging are tightly linked. Insufficient or poor-quality sleep is associated with accelerated biological deterioration across neural and peripheral systems, contributing to biological aging and to earlier onset or faster progression of neurodegenerative, cardiovascular, and metabolic disorders [1,2]. Conversely, aging alters sleep amount, quality, continuity, circadian organization, and increases primary sleep disorders [1,3,4,5]. Quantitative EEG-derived sleep metrics are therefore increasingly viewed as candidate biomarkers of biological aging [6,7,8].

Rodent models, particularly mice, offer unique advantages for mechanistic investigations of sleep and aging due to their genetic accessibility, evolutionary conservation of sleep–wake regulation, and suitability for invasive physiological measurements [9,10]. Age-associated changes in sleep–wake dynamics show broad parallels between mice and humans, including increased vigilance states instability, reflected by waking disruption with frequent sleep intrusions and greater sleep during the active (dark) period, analogous to heightened daytime sleepiness in humans [6,7,9,10,11,12], although a few studies found these changes insignificant [9]. While findings on non-rapid eye movement (NREM) sleep during the resting (light) phase remain inconsistent, with reports of either reduced or unchanged amounts [6,7,12], REM sleep episodes consistently shorten with age [7,9,10,12]. These inconsistencies may be due to methodological differences among studies, including strain background, age definitions, recording duration, and temporal binning strategy [6,7,9,10,13].

Conventional analysis of vigilance states typically includes (1) quantifying total duration of wake, NREM, and REM across light/dark periods, (2) assessing vigilance state fragmentation, and (3) spectral analysis of slow-wave activity during NREM sleep. Notably, most rodent studies have relied on only one to three days of sleep–wake recordings, often restricted to male cohorts, which may be insufficient to capture intra-individual variability. Female rodents present additional challenges, as sleep architecture may fluctuate across the estrous cycle [14,15], requiring hormonal/estrous staging that introduces stress and analytical complications. Whether males or older females exhibit comparable day-to-day variability is unknown, raising concerns that few-day sampling may be insufficient for robust quantitative and qualitative assessment of sleep architecture [10,16].

Despite considerable progress, a major limitation that remains is the lack of standardized and sensitive analytical frameworks capable of detecting subtle and biologically meaningful age- and sex-dependent variations in sleep architecture across studies [6,16]. As populations age, sensitive and reproducible EEG-based markers are increasingly needed, for tracking biological aging, for developing interventions, and importantly for aligning rodent and human findings for translational relevance.

In this study, we applied a multiscale analytical framework to 14 days of continuously recorded EEG from 24 C57BL/6J mice in a balanced 2 × 2 design (age: young 4 months vs. old 24 months; sex: male vs. female). This approach addressed key limitations in the field, including short recording durations, male-biased cohorts, intra-individual and estrous-cycle variability, and the lack of standardized methods for detecting subtle age- and sex-dependent changes.

The novelty of the study lies in its prolonged, sex-balanced recording design combined with an integrated multi-level analysis that extends beyond conventional sleep–wake state summaries. Rather than relying on a single metric or timescale, we systematically characterized sleep–wake architecture across seven complementary levels that include (1) four-state vigilance classification into active theta-dominant wake (TDW), quiet non-theta-dominant wake (nTDW), NREM, and REM sleep using a validated automated classifier; (2) circadian and ultradian structure; (3) bout-length-specific fragmentation; (4) transition dynamics; (5) ultradian block organization, including a statistical framework for distinguishing consolidated NREM–REM blocks from brief “out-block” episodes; (6) full-spectrum EEG analysis; and (7) variance-based assessment of metric stability and statistical power. Together, this framework provides a coherent view of sleep organization across multiple temporal and analytical scales, enabling detection of patterns that are not apparent at any single level. In particular, ultradian block analysis captures sleep organization at an intermediate scale between individual bouts and circadian structure, an aspect that has been largely underexplored. In addition, for the first time, variance-based analyses quantify the relative contributions of sample size and recording duration, offering practical guidance for designing future sleep-aging research studies, particularly those based on small pilot cohorts.

The integrated approach used in this study revealed age-related changes typically masked by short recordings, male-only cohorts, one-state wake scoring, or coarse averaging. Key findings included altered dark-phase wake quality, disrupted NREM-exit architecture, increased isolated sleep fragments, and temporally localized spectral signatures of aging. Across analytical levels, we identified a recurrent temporal pattern at the dark-to-light transition, a late-dark NREM rise, a transient pre-lights-on wake surge, and an early-light NREM peak, visible in vigilance-state composition, bout dynamics, transition probabilities, block-level assembly, and spectral organization, and where age × sex effects concentrated. Ultradian block analysis further identified “out-block” NREM episodes, isolated NREM sleep fragments that fail to assemble into full NREM–REM ultradian cycles, as a distinct and poorly consolidated sleep event that increased with aging, offering a candidate marker of consolidation failure distinct from conventional fragmentation indices. Variance analyses further showed that sample size, not recording length, is the primary determinant of statistical power in sleep-aging studies.

By integrating circadian, ultradian, episode-level, transition-level, spectral, and variance perspectives within a single balanced cohort, this study provides a high-resolution, scalable framework for characterizing age- and sex-dependent features of sleep–wake architecture in mice and for prioritizing candidate endpoints for future translational work.

## 2. Materials and Methods

### 2.1. Experimental Subjects

All experiments were conducted in accordance with the National Institutes of Health Guide for the Care and Use of Laboratory Animals and were approved by the Institutional Animal Care and Use Committee (IACUC) of the VA Greater Los Angeles Healthcare System.

A total of 24 adult C57BL/6J mice were studied in a balanced 2 × 2 factorial design crossing age (young, 4 months; old, 24 months) with sex (male, female), yielding four groups of *n =* 6 each: young male (YM), young female (YF), old male (OM), and old female (OF). Animals were housed individually in Plexiglas cages within sound-attenuated, temperature-controlled (24 ± 2 °C) recording chambers under a 12:12 h light–dark cycle (lights on at 07:00 h, ZT0; 100–150 lux) with food and water available ad libitum. The study was conducted under undisturbed conditions without experimental intervention. Animals were implanted with chronic EEG/EMG electrodes for 14 days of continuous polysomnographic recording.

Surgical procedures were described previously [7,17]. Briefly, under anesthesia with ketamine (ketamine hydrochloride injection; Hikma Pharmaceuticals USA Inc., Berkeley Heights, NJ, USA; manufactured by Hikma Farmacêutica, Portugal) and xylazine (AnaSed; Akorn Animal Health Inc., Lake Forest, IL, USA) (100 + 15 mg/kg), maintained with 1–2% isoflurane (Piramal Critical Care Inc., Bethlehem, PA, USA; manufactured by Piramal Pharma Ltd., Telangana, India), and under aseptic conditions, EEG and EMG electrodes were implanted for polygraphic monitoring of sleep–wake states. For unilateral bipolar EEG recording, two stainless steel screw electrodes were placed at 1 mm rostral/1 mm lateral and 4 mm posterior/2 mm lateral to bregma. Two flexible silver wires (A-M Systems, Sequim, WA, USA) were implanted into the dorsal cervical musculature. All electrodes were soldered to a miniature connector and secured with dental acrylic.

Estrous cycle staging was not performed, which is a limitation. However, the 14-day recording window likely captured two to three estrous cycles in young female C57BL/6J mice (cycle length ≈ 4–5 days), and 14-day averaging was intended to emphasize stable circadian features rather than cycle-dependent fluctuations. This likely reduced estrous-related variance at the group level but could not eliminate within-animal cycle effects. Accordingly, comparisons involving young females, especially age comparisons within females and sex comparisons, should be interpreted cautiously; cycle-stratified analyses with vaginal cytology or endocrine measures will be needed in future work.

### 2.2. Data Acquisition

EEG and EMG signals were amplified using an A-M Systems Model 3500 AC/DC amplifier (A-M Systems, Sequim, WA, USA), band-pass filtered at 1–100 Hz for EEG and 10–300 Hz for EMG, and notch filtered at 60 Hz. Signals were digitized at 200 Hz using a CED Micro1401 acquisition system (Cambridge Electronic Design, Cambridge, UK) running Spike2 software (Version 6) for off-line analysis. Continuous recordings were performed for up to three weeks to obtain stable multi-day datasets. Bedding-change days were excluded because they perturb sleep–wake dynamics. To maintain balanced sampling across animals and groups, 14 consecutive undisturbed days were retained for each mouse. Mice were euthanized at study completion with pentobarbital sodium (100 mg/kg, i.p.) (Fatal-Plus, 390 mg/mL; Vortech Pharmaceuticals Ltd., Dearborn, MI, USA).

### 2.3. Vigilance-State Scoring and Validation

Although both EEG and EMG were acquired, vigilance-state scoring was performed using single-channel EEG only. EEG-only scoring was used because the automated classifier applied here [18] operates on single-channel EEG. EMG was acquired during recording but was not used for vigilance-state scoring or subsequent analyses. Continuous recordings were segmented into non-overlapping 10 s epochs (360 epochs/h; 8640 epochs/24 h), and each epoch was first classified as wakefulness (W), NREM sleep, or REM sleep using a previously validated automated scoring algorithm [18]. Wake epochs were then subdivided into theta-dominated wakefulness (TDW), an operational surrogate of active waking characterized by a dominant 7–12 Hz rhythm, and non-theta-dominated wakefulness (nTDW), corresponding to quiet waking, using a spectral-shape algorithm adapted from Vassalli and Franken [19].

A theta prominence index was computed for each wake epoch as θr = P(7–12 Hz)/P(3.5–45 Hz), where P denotes integrated power spectral density across the specified band; epochs with θr > 0.228 were classified as TDW and otherwise as nTDW. This two-step procedure yielded a four-state classification: TDW, nTDW, NREM, and REM. Throughout this manuscript, TDW refers to active wakefulness with prominent theta-range activity in the recorded cortical derivation (consistent with, but not a direct measure of, hippocampal theta), and nTDW refers to quiet wakefulness without dominant theta. Artifact detection and rejection were performed within the SAGER scoring pipeline [18], and artifact-flagged epochs were excluded from subsequent analyses (per-group artifact burden 1.0–1.4%).

To confirm that the age effect on dark-phase TDW% was not an artifact of the θr = 0.228 threshold, TDW/nTDW classification was recomputed at seven θr values (0.18, 0.20, 0.22, 0.228, 0.24, 0.26, 0.28) on the 3-day spectral subset used for PSD analyses. Between-group contrasts were evaluated at each threshold using Hedges’ g with 10,000-resample bootstrap 95% confidence intervals, Welch’s *t*-test for the pooled young vs. old contrast (df = 22), and Holm–Bonferroni correction for sex-stratified contrasts (Supplementary Figure S1).

### 2.4. Analytic Data Hierarchy

All variance-decomposition, temporal-stability, power, and optimal-allocation analyses used the full retained 14-day dataset (24 mice × 14 days × 8640 epochs/day = 2,903,040 epochs). The same dataset was used for artifact and sparse-bin quantification. Only spectral analyses (Section 2.11) used a shorter 3-day subset.

Spectral analyses used a 72 h subset per mouse, selected on the basis of signal quality and artifact burden before any group-level spectral comparisons. This duration is consistent with the recording windows commonly used for rodent sleep-EEG spectral analysis and provided sufficient artifact-free epochs for state-conditioned PSD estimation. Full 14-day vigilance-state analyses showed no systematic acclimation effect or secular drift in sleep–wake metrics (Supplementary Note S1), and the dark-phase TDW age effect was preserved within this subset across seven TDW/nTDW classification thresholds (Supplementary Figure S1).

### 2.5. Data Aggregation and Preprocessing

#### 2.5.1. Vigilance-State Percentage Analyses

Hypnograms were compiled into a unified database annotated with animal ID, age, sex, and recording day. All analyses, tables, and figures were generated using customized Python v3.11 pipelines (numpy, scipy, pandas, pyGAM, statsmodels), supplemented by R v4.3.3 (glmmTMB v1.1.8, lme4 v1.1.35.1, emmeans v1.10.0) for selected GLMM and planned-contrast analyses. Spectral parameterization used specparam (formerly FOOOF). Python package versions were not separately archived for the analysis environment; the Python interpreter (v3.11) and all R package versions are reported.

For vigilance-state percentage analyses, hourly state percentages were averaged across 14 days for each mouse, yielding one 24-point circadian profile per mouse per state. For phase-level analyses, these hourly profiles were collapsed into dark-phase (ZT12–ZT0) and light-phase (ZT0–ZT12) means. This averaging strategy estimated each animal’s stable circadian phenotype while reducing transient day-to-day variability, at the cost of explicit day-level variance; the implications of this trade-off are addressed in Supplementary Note S1.

#### 2.5.2. NREM Temporal Architecture

For NREM temporal architecture analyses, epochs were aggregated into 15 min bins (90 epochs/bin; 96 bins/24 h). For each animal, a 14-day mean profile was computed by averaging state percentages across days within each bin, yielding one representative 24 h profile per state. Profiles were smoothed with a Savitzky–Golay filter (window = 5 bins, polynomial order = 2). Day-level validation confirmed that major findings persisted at the daily level.

#### 2.5.3. Episode Identification

A vigilance-state episode was defined as a contiguous run of identical state codes. Episodes were identified by run-length encoding applied to the scored epoch sequence for each animal and recording day.

#### 2.5.4. EEG Spectral Processing

For whole-spectrum analyses, one power spectral density (PSD) curve was computed for each mouse in each state × phase combination by averaging Welch periodograms (4 s Hanning sub-windows, 50% overlap) from all artifact-free epochs assigned to that state and phase. Spectra were evaluated on an interpolated 0.1 Hz grid from 1.0 to 20.0 Hz, with absolute PSD (log10 µV^2^/Hz) as the primary representation.

For hourly spectral-distribution analyses, power was collapsed into nineteen 1 Hz bands spanning 1–20 Hz. Mean spectral power was calculated across all clean epochs in each mouse × day × hour × state cell (cells with < 6 clean epochs excluded), and then averaged across days to yield one 24 h profile per state × band combination.

#### 2.5.5. Sparse-Bin Quantification

For each mouse × ZT hour × vigilance state, the number of clean epochs was computed for each recording day. Bins containing fewer than 12 clean epochs/day (<2 min within a 1 h window) were flagged as sparse. Sparsity was assessed per day across all 14 days. REM accounted for most sparse bins (10.4% vs. 1.7% for TDW, 0% for nTDW, 1.2% for NREM), reflecting its brief and intermittent nature.

#### 2.5.6. Compositional Constraint

Because TDW, nTDW, NREM, and REM sum to 100% in every time bin, the states are compositional. Although centered log-ratio (CLR) transformation can address this dependency [20], primary analyses modeled each state separately using linear mixed models on percentage values, because vigilance states have distinct circuit-level correlates and percentage-based effects are more directly interpretable than CLRs. To verify that this strategy did not bias the main conclusions, all primary vigilance-state percentage analyses were replicated using CLR-transformed data (Supplementary Methods S1).

### 2.6. Statistical Analysis: Twenty-Four-Hour Vigilance-State Profiles

A three-tier inferential framework was used: phase-level mouse-based contrasts (Tier 1, confirmatory), omnibus linear mixed models on hourly profiles (Tier 2, global profile shape), and cascade-gated hourly estimated marginal mean contrasts (Tier 3, exploratory localization). Only the phase-level contrasts were treated as formally confirmatory. Throughout the Results, Tier 1 phase-level effects are referred to as primary (confirmatory) findings, Tier 2 omnibus and landmark analyses as secondary/supporting, and Tier 3 localized, sex, and interaction effects as exploratory and hypothesis-generating. Full specifications, including multiplicity control, parametric bootstrap procedures for borderline likelihood-ratio tests, and harmonic alternatives to categorical hourly parameterization, are provided in Supplementary Methods S3.

Tier 1 between-group comparisons used independent-samples *t*-tests (df = 10); within-group dark-versus-light comparisons used paired *t*-tests (df = 5). The primary Holm–Bonferroni family comprised four pairwise between-group contrasts per state × phase cell (old vs. young within each sex, plus male vs. female within each age group). Effect sizes were expressed as Hedges’ g with 10,000-resample bootstrap 95% confidence intervals.

With *n* = 6 per group, the design provided ≥80% power only for large effects (Cohen’s d ≥ 1.80, e.g., dark-phase TDW reduction); for moderate effects (d = 1.0–1.5), power declined to ~45–55%, and age × sex interactions were detectable only if very large (Supplementary Note S1). Accordingly, corrected large effects are treated as primary, whereas sex differences, interactions, and moderate effects are treated as hypothesis-generating. Non-significant differences were not interpreted as equivalence.

### 2.7. Statistical Analysis: NREM Temporal Architecture (Section 3.2)

A hierarchical analytical sequence was applied: circadian whole-profile contrasts via group-specific GAMs (Phase 1), ultradian oscillation characterization (Phase 2), N-shape landmark extraction within the predefined ZT16–ZT6 window (Phase 3), state-composition analysis at transitions (Phase 4), and robustness/sensitivity checks (Phase 5). The N-shape window was defined a priori from published 24 h profiles [9,10,12,21] to capture the late-dark NREM rise, pre-lights-on wake increase, and early-light NREM surge. Three inferential tiers were used (Tier 1 confirmatory, Tier 2 Holm-corrected, Tier 3 exploratory). Full phase-by-phase specifications—GAM bootstrap procedures, peak-detection settings, landmark extraction with circular-statistics handling for trough ZT, binomial GLM for wake microcomposition, and parameter sensitivity—are reported in Supplementary Methods S4.

The primary confirmatory endpoint was the TDW fraction at ZT16, a fixed pre-specified time point corresponding to N-shape onset. It was tested in a factorial age × sex ANOVA (OLS, Type II SS) at the 14-day-averaged level (*n* = 24), with day-level validation in a mixed-effects model (*n* = 320 animal-days). ZT16 was chosen a priori because it marks the expected TDW peak in the N-shape framework; the other four pre-specified points (ZT20, ZT23.5, ZT0.5, ZT3) were treated as supporting analyses. Because ZT16 was fixed before inspection of the data, it is robust to selection bias affecting data-driven landmarks. The Tier 2 trough analysis characterizes where within the ZT20–ZT2 window the age-related TDW suppression is maximal and is supportive rather than confirmatory.

### 2.8. Statistical Analysis: Episode Architecture and Bout-Length Distributions (Section 3.3)

Episode architecture was quantified by mean episode duration and episode count for each state, with phase-level duration and count as the primary outcomes. Bout-length distributions were analyzed using a complementary three-tier framework: Tier 1 used Cox proportional hazards survival models with cluster-robust standard errors (Mouse_ID as cluster); Tier 2 used per-mouse summary metrics (mean/median duration, short/long-bout fractions, bout rate, duration CV) and bi-exponential mixture decomposition; Tier 3 examined binned bout-length distributions across 3 h circadian segments. Holm–Bonferroni correction was applied within each state for primary between-group contrasts. Full survival-model, AFT, mixture-model, and distributional specifications are reported in Supplementary Methods S5.

### 2.9. Statistical Analysis: NREM Sleep Transition Dynamics (Section 3.4)

State-Transition Quantification: Two transition types were quantified from consecutive epoch pairs: NREM → wake (NREM followed by TDW or nTDW) and NREM → REM (NREM followed by REM). Transition counts were computed for each mouse × day × clock hour, yielding 8064 observations. Because both transitions are conditional on NREM occurrence, counts were modeled as rates normalized to NREM opportunity using a log-offset for NREM-epoch count. Hours with zero NREM epochs (745/8064 observations, 9.2%; predominantly dark-phase sustained wakefulness) were excluded, leaving 7319 hourly observations.

NREM → Wake and NREM → REM Models: NREM → wake counts were modeled with a negative binomial (NB) generalized linear mixed model (GLMM) to account for overdispersion (ΔAIC vs. Poisson = 491.3). NREM → REM counts were modeled similarly; zero-inflated NB was evaluated but not adopted because zero inflation was non-significant and AIC favored the simpler NB model. Randomized quantile residual diagnostics confirmed adequate fit.

Both primary models included age × sex × phase (dark/light) as fixed effects, with log(NREM epochs) as an offset to model transition rates per unit NREM exposure. This formulation ensured that age effects reflected altered NREM exit dynamics rather than differences in NREM quantity. Random intercepts for Mouse and Mouse:Day accounted for individual and day-to-day variability. The model in R/glmmTMB syntax is count ~ age × sex × phase + offset(log(NREM_epochs)) + (1|Mouse) + (1|Mouse:Day). Supplementary analyses (circadian cosinor model, binomial REM-gating model, 2 h aggregation sensitivity, no-offset robustness check) are reported in Supplementary Methods S6.

Fixed effects were evaluated using Type III Wald χ^2^ tests. Planned post hoc contrasts were extracted from estimated marginal means (EMMs; emmeans v1.10.0) and reported as rate ratios (RR = exp(β)) with 95% confidence intervals. All rate ratios were expressed in the biologically interpretable direction: old/young for age and female/male for sex. Twelve planned contrasts per model were Holm-adjusted.

### 2.10. Statistical Analysis: Ultradian Block Architecture (Section 3.5)

Ultradian Block Formation and Classification: Ultradian sleep blocks were defined as continuous sequences of NREM and REM sleep in which intervening wake episodes ≤ 3 min (≤18 consecutive 10 s wake epochs) were bridged as intra-block brief wakefulness; wake episodes > 3 min terminated the block. First and last partial blocks at 24 h boundaries were excluded. This 3 min bridging threshold follows established approaches to rodent ultradian block segmentation [22,23].

Single-state blocks (NREM-only or REM-only, without NREM ↔ REM transitions) were classified as out-block episodes if both (i) total duration was <500 s (<50 epochs) and (ii) separation from the nearest adjacent sleep episode was ≥180 s on both sides. Blocks failing either condition were retained as valid blocks. Out-block episodes were excluded from primary circadian-bin analyses but modeled separately as a marker of failed sleep consolidation. The 500 s duration and 180 s separation criteria were chosen empirically from the bimodal distribution of single-state block durations and are intended as operational definitions rather than physiological constants; their robustness was confirmed across alternative threshold settings (Supplementary Methods S2; Supplementary Table S30). This procedure identified 5333 valid ultradian blocks and 2828 out-block episodes across all recordings. Threshold rationale and sensitivity analyses are reported in Supplementary Methods S2.

Statistical Models for Block Architecture: Generalized linear mixed models (GLMMs) were fitted with fixed effects of age, sex, bin (8 levels of 3 h circadian segments), and their full factorial interactions, with random intercepts for Mouse and Mouse:Day. Model families were matched to distributional properties: Gamma GLMM with log link for duration, Beta GLMM with logit link for state fractions, and NB GLMM for counts and transitions. Because intra-block wake, NREM, and REM fractions sum to unity, these metrics are compositionally constrained. Omnibus tests used Type III Wald χ^2^ statistics supplemented by likelihood-ratio tests for the three-way interaction. Post hoc contrasts tested age × sex effects within dark and light phases with FDR correction.

Population-level cosinor analysis included cosine and sine harmonic terms within the GLMM; per-mouse cosinor parameters were tested via age × sex ANOVA. Supplementary 6 h analyses examined time allocation, fragmentation rate, distributional shape, and out-block occurrence/intensity.

### 2.11. Statistical Analysis: EEG Spectral Analysis (Section 3.6)

The spectral analysis was organized across three tiers: Tier 1, primary whole-spectrum inference using cluster-based permutation testing (CBPT) of absolute PSD; Tier 2, temporal-allocation analysis using hourly normalized spectral profiles under three normalization frameworks; and Tier 3, interpretive analyses using relative PSD, scalar band-power summaries, and spectral parameterization (FOOOF/specparam). All formal whole-spectrum significance claims are based on CBPT.

CBPT was applied separately within each state × phase stratum to full-resolution absolute PSD curves. At each frequency bin, a linear model with age, sex, and age × sex fixed effects (deviation-coded) yielded t-statistic profiles across 1.0–20.0 Hz. Adjacent bins exceeding |t| > 2.086 were grouped into clusters, with cluster mass defined as the sum of t-statistics. Cluster-mass null distributions were generated from 5000 mouse-level permutations of factorial group labels, controlling family-wise error at α = 0.05. Detailed bin-wise models, frequency-interaction tests, effect-size quantification, hourly normalization frameworks, relative-PSD analyses, scalar band-power analyses, and FOOOF/specparam parameterization are reported in Supplementary Methods S6.

### 2.12. Methodological Limitations of Spectral Analysis

Several limitations should be considered when interpreting the spectral results. First, normalization-based temporal-allocation analyses address redistribution of spectral power across the day rather than absolute amplitude; effects visible only in absolute PSD may be suppressed by normalization. Second, state-specific data density differed markedly, especially in REM, where 15.7% of cells contained fewer than 12 clean epochs compared with 1.1% for NREM, potentially reducing estimator stability. Third, because state occupancy may vary across age and sex groups, estimator precision may differ systematically across the design. Fourth, the hourly cell-wise framework does not explicitly model temporal or spectral autocorrelation; isolated effects were therefore interpreted cautiously, whereas recurrent effects across adjacent time-frequency loci were given greater weight. Fifth, the bin-wise models used in the absolute PSD analysis are secondary and do not replace CBPT for primary inference; scalar band-power summaries are lossy compressions of frequency-resolved data, and FOOOF fits are model-dependent. Finally, with *n* = 6 per group and a single cortical derivation, power for higher-order interactions is limited and topographic generalization should be cautious. Variance-decomposition methodology, dataset-validation analyses, and design-implication results are reported separately in Supplementary Note S1. To characterize the detectable effect sizes of the present design and to inform future studies, we performed a variance-component-based design and sensitivity analysis that quantifies detectable effects and the relative value of adding animals versus recording days (Supplementary Note S1, Table S32); this analysis indicates that the design is powered mainly for large effects, so sex-related and interaction findings are treated as hypothesis-generating.

## 3. Results

### 3.1. Twenty-Four-Hour Vigilance-State Profiles

#### 3.1.1. Cohort Integrity and Dataset Completeness

All 24 mice completed the 14-day recording protocol without data loss, yielding a total of 8064 h of observations with complete coverage. Within-mouse coefficient of variation (CV) across the 14 recording days was highest for TDW (0.76 ± 0.40) and REM (0.74 ± 0.39) and lowest for nTDW (0.49 ± 0.22), reflecting the inherently greater variability of states that occupy smaller proportions of total time. Five young mice exhibited CVs exceeding 0.80 in at least one state, but all were retained in primary analyses; a sensitivity analysis excluding these animals confirmed that all primary conclusions were robust.

#### 3.1.2. Age and Sex Effects on 24-h Vigilance-State Profiles

The age × hour interaction was highly significant for all four vigilance states by ML likelihood-ratio test: TDW (χ^2^(23) = 258.2, *p* < 0.0001), nTDW (χ^2^(23) = 151.6, *p* < 0.0001), NREM (χ^2^(23) = 110.4, *p* < 0.0001), and REM (χ^2^(23) = 81.7, *p* < 0.0001). Sex × hour interactions were significant in three of the four states—TDW (χ^2^(23) = 74.4, *p* < 0.001), NREM (χ^2^(23) = 67.3, *p* < 0.001), and REM (χ^2^(23) = 46.7, *p* = 0.002)—and marginal in nTDW (χ^2^(23) = 38.1, *p* = 0.025 by asymptotic test; *p* = 0.066 by parametric bootstrap with 500 resamples). The Tier 2 hour-containing interaction tests remained directionally consistent across alternative time-factor parameterizations (Supplementary Table S1). These results establish that aging reshapes the circadian distribution of all four states, not merely their 24 h totals, and that sex-related differences in circadian state profiles are also present in at least three of the four states. Critically, the three-way age × sex × hour interaction did not reach significance for any state (all χ^2^(23) < 34, all *p* > 0.07), indicating that neither the age-related nor the sex-related reshaping of circadian vigilance-state profiles depended on the other factor in this sample; however, these omnibus effects translated into limited confirmatory sex differences at the phase level (see below). Model diagnostics including variance components and convergence indices are summarized in Supplementary Table S2, and Nakagawa’s marginal and conditional R^2^ values quantifying the variance explained by fixed and random effects are reported in Supplementary Table S3. Per-mouse autocorrelation diagnostics of LMM residuals (lag-1 ACF, Ljung–Box test, and Bartlett-effective sample size) are reported in Supplementary Table S4, and graphical convergence and residual diagnostics are presented in Supplementary Figure S2 (residual diagnostics) and Supplementary Figure S3 (LMM convergence and optimizer stability).

#### 3.1.3. Aging Reduces Dark-Phase TDW in Both Sexes

**Figure 1:**
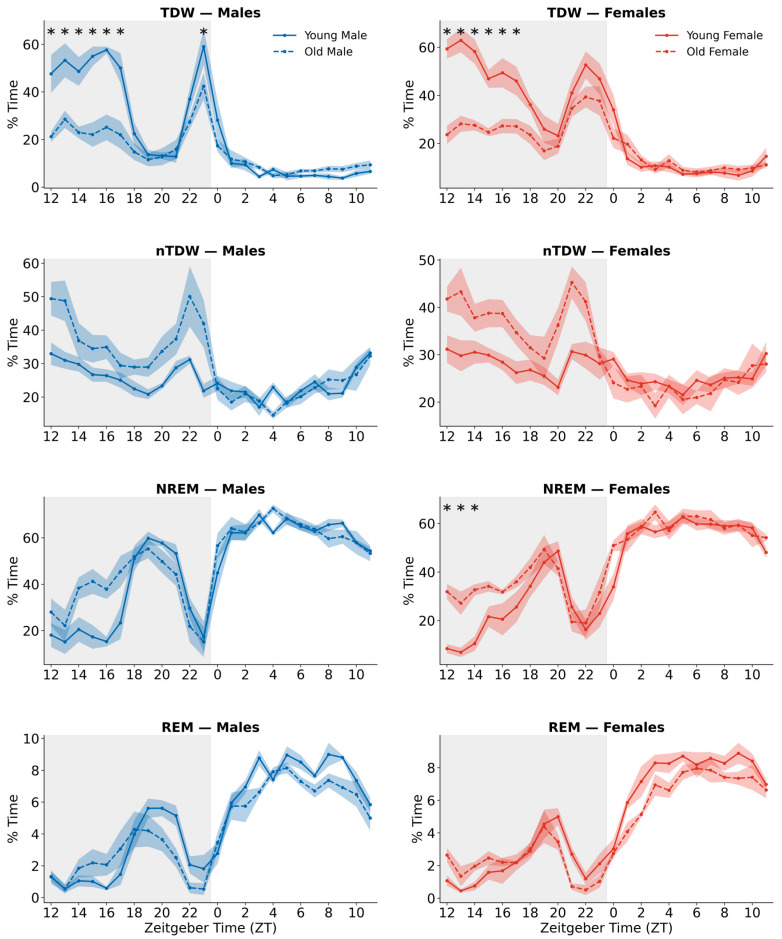
Age reshapes the 24 h distribution of vigilance-state occupancy in both sexes. Hourly percentage of time (% per ZT hour) in four vigilance states, TDW, nTDW, NREM, and REM, across the 24 h cycle (ZT0 = lights-on) in young (4-mo) and old (24-mo) C57BL/6J mice. Each trace is the group mean of per-animal 14-day means; shading = SEM. Gray shading: dark phase (ZT12–ZT0). Stars: hourly age contrasts (young vs. old within sex), *p*Holm < 0.05 after cascade-gated Holm correction within each 12 h family (Section 2.6); confirmatory phase-level inference in Figure 2. *n* = 6/group (YM, YF, OM, OF); *n* = 24.

**Figure 2:**
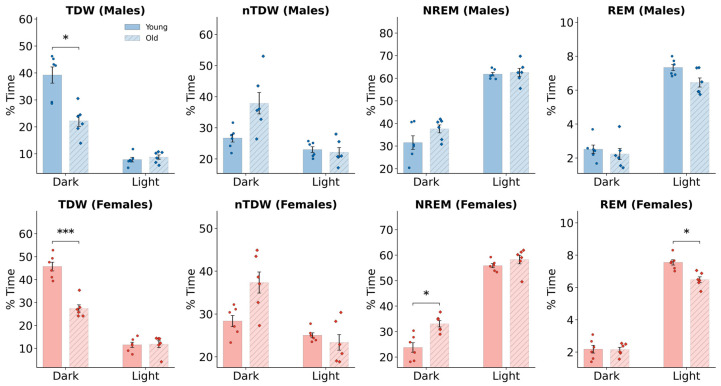
Age reduces dark-phase TDW in both sexes and reshapes sleep–wake architecture. Group means ± SEM of the percentage of time in TDW, nTDW, NREM, and REM during the dark (ZT12–ZT0) and light (ZT0–ZT12) phases, computed from per-animal 14-day means (dots) in young and old mice. Stars denote Tier 1 confirmatory Holm-corrected age differences within sex (Section 2.6). *, *p*Holm < 0.05, ***, *p*Holm < 0.001; absence of a star above a bracket indicates pHolm ≥ 0.05. *n* = 6/group (YM, YF, OM, OF); *n* = 24.

The most prominent finding was a large, dark-phase-specific reduction in TDW with aging. Old females showed an 18.3 percentage-point reduction in dark-phase TDW relative to young females (Hedges’ g = −3.60 [95% CI: −7.91, −2.53]; *p*Holm < 0.001), and old males showed a 17.0 percentage-point reduction relative to young males (g = −2.26 [−6.82, −1.33]; *p*Holm = 0.005). These are among the largest effect sizes observed in the entire dataset and exceed the minimum detectable |d| of 1.80 determined by the sensitivity analysis (Figure 1 and Figure 2; Supplementary Table S5). In contrast, light-phase TDW was uniformly low across all groups (7.9–11.8%) and showed no significant age or sex differences, indicating that the aging effect is confined to the active (dark) phase.

The dark–light TDW amplitude—the difference between dark-phase and light-phase TDW—was markedly attenuated in old mice. Young females showed a dark–light amplitude of +34.1 [+26.8, +41.5] percentage points, while old females showed only +15.6 [+10.4, +20.8], representing a greater than twofold reduction in the circadian contrast of TDW. This amplitude attenuation was similar in males. A two-harmonic cosinor model provided a compact low-dimensional summary consistent with these observations: TDW overall amplitude (peak-to-trough half-range) was reduced by more than half in old mice (young: 27–28%; old: 10–12%), with the dark-phase peak occurring near ZT 14 in young animals and showing a modest phase redistribution with aging that was more pronounced in females than in males (Supplementary Figure S4; Supplementary Table S6).

#### 3.1.4. Reciprocal Redistribution of Vigilance States

The loss of dark-phase TDW in aged mice was accompanied by a reciprocal redistribution toward quieter vigilance states. Old females exhibited a +9.3% increase in dark-phase NREM (g = +2.02 [+1.26, +4.20]; *p*Holm = 0.014). Dark-phase nTDW was also elevated in old mice (+9–11%), with effect sizes exceeding 1.4, but these contrasts did not survive Holm correction (*p*Holm = 0.058 for both sexes), narrowly missing the significance threshold (Supplementary Figure S5; Supplementary Table S5). The magnitude and direction of these reciprocal changes are consistent with the compositional constraint (states sum to 100%) but also with a biologically meaningful shift from consolidated active wakefulness toward fragmented, drowsy states—a pattern elaborated by the episode-level and transition analyses in Section 3.3, Section 3.4 and Section 3.5.

In addition, old females exhibited a small but significant reduction in light-phase REM sleep (−1.1%, g = −2.17 [−4.22, −1.49]; *p*Holm = 0.009; visible as asterisk bracket in Figure 2 and as a Holm-surviving contrast in the Supplementary Figure S5 forest plot). This effect, while modest in absolute magnitude, represents a substantial relative decrease (~15% of baseline REM time) and represents a small reduction that will require bout-level and spectral analyses to interpret mechanistically. The effect was directionally similar in old males (−0.9%, g = −1.32) but did not reach significance after correction (*p*Holm = 0.097), consistent with the reduced power for moderate effects noted above. Hourly localization of all phase-level effects is provided in Supplementary Figure S6.

#### 3.1.5. Effect Sizes for Phase-Level Age Contrasts

Hedges’ g and bootstrap 95% confidence intervals were computed for all 16 between-group age contrasts (4 states × 2 phases × 2 sex strata; Figure 2 and Figure S5; Supplementary Table S5). Four contrasts survived Holm–Bonferroni correction: dark-phase TDW in both sexes (females: g = −3.60 [−7.91, −2.53], *p*Holm < 0.001; males: g = −2.26 [−6.82, −1.33], *p*Holm = 0.005), dark-phase NREM in females (g = +2.02 [+1.26, +4.20], *p*Holm = 0.014), and light-phase REM in females (g = −2.17 [−4.22, −1.49], *p*Holm = 0.009). All four exceeded |d| = 1.80, the minimum detectable effect size at 80% power for the current design. The remaining 12 contrasts ranged from |g| = 0.04 (dark-phase REM in females) to |g| = 1.57 (dark-phase nTDW in females), with confidence intervals consistently including zero after correction. The dark-phase nTDW contrasts in both sexes (g = +1.49 to +1.57) are noteworthy as provisional findings: their effect sizes are large, their confidence intervals narrowly miss excluding zero, and they are directionally consistent with the TDW-to-nTDW redistribution pattern, but they did not survive Holm correction at *n* = 6. Overall, the largest effects cluster in dark-phase wake states (TDW reduction, nTDW increase), followed by dark-phase NREM (increase in females), while light-phase and REM contrasts are generally small and imprecise.

#### 3.1.6. Decomposition of Sex × Hour Interactions

The sex × hour interaction was significant in three of the four vigilance states (TDW, NREM, and REM) and marginal in nTDW (Section 3.1.2), indicating that sex differences in vigilance-state time varied across the 24 h cycle in most states. Decomposition of this interaction by phase revealed one contrast that survived Holm correction: young females had 5.9 percentage points less light-phase NREM than young males (g = −2.60 [−4.96, −1.81]; *p*Holm = 0.005; Supplementary Table S5). Other sex contrasts showed modest effect sizes (g = 0.89–0.97 for TDW) without approaching significance after correction; the broader landscape of sex contrasts and their effect-size magnitudes is summarized in Supplementary Figure S7, with the corresponding Hedges’ g forest plot in Supplementary Figure S8 showing dark-phase wake-state contrasts clustering near |g| = 0.9–1.4 with confidence intervals that cross zero after correction.

#### 3.1.7. Robustness Analyses

All five significant contrasts preserved their direction when five high-CV mice were excluded from the analysis. Four of the five attenuated by 16–45% (Supplementary Table S7); the NREM light-phase sex contrast in young mice (Δ 5.9 → 7.6 pp) instead strengthened after exclusion, reflecting that the excluded animals were close to the YF group mean on this metric. The Hedges’ g correction reduced all effect sizes by approximately 10% relative to Cohen’s d, and all bootstrap 95% CIs for significant contrasts excluded zero. Sensitivity analysis confirmed that the minimum detectable |d| at 80% power was 1.80 for between-group and 1.44 for paired contrasts; all significant contrasts exceeded these thresholds (Supplementary Figure S9 [age-related effect-size hot zones in early-to-mid dark phase], and Supplementary Figure S10 [full set of 32 phase-level contrasts]).

The dark-phase age effect on TDW% was additionally robust to the TDW classifier’s θr threshold: across a sweep from 0.18 to 0.28 bracketing the baseline 0.228, the pooled young vs. old contrast yielded Hedges’ g = 1.20–1.60 with Welch *p* ≤ 0.007 at every threshold, and the direction of the effect (young > old) was preserved at all seven tested thresholds and both phases (Supplementary Figure S1).

### 3.2. NREM Temporal Architecture: Ultradian Oscillations and N-Shape Landmarks

#### 3.2.1. Phase 1: The 24-Hour NREM Profile Diverges by Age and Sex in the Dark Phase

**Figure 3:**
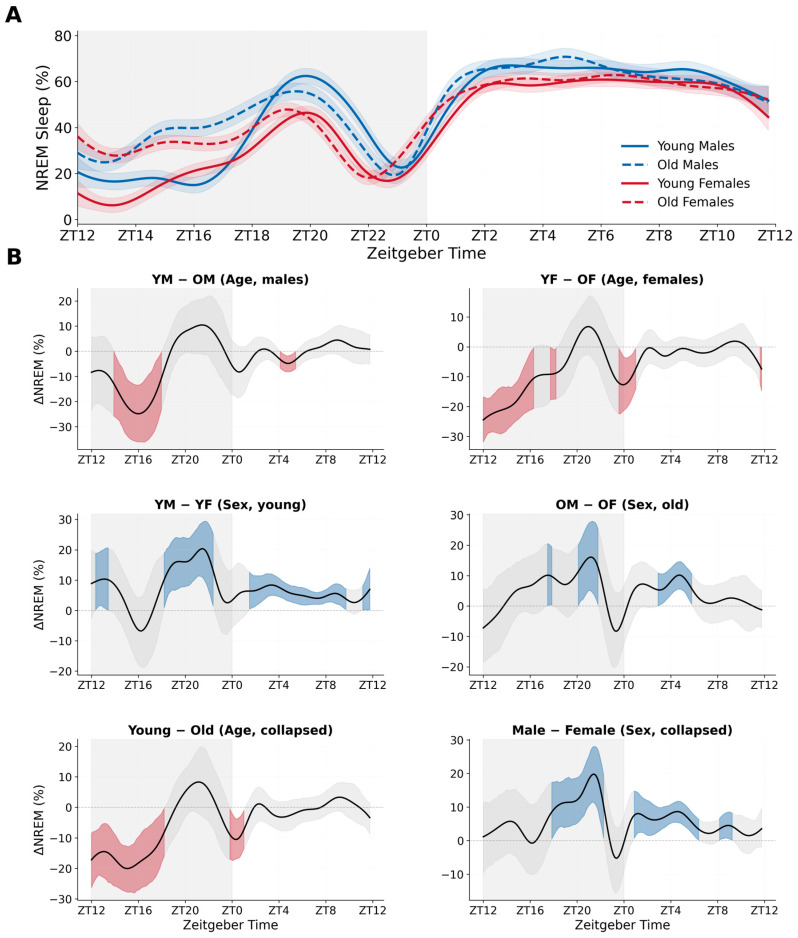
Age-related NREM elevation localizes to the early-mid dark phase; sex differences localize to late-dark and early-light intervals. (**A**) Group-specific GAM fits (20 thin-plate splines, cubic) for 24 h NREM profiles (15 min bins) with 95% CIs. YM solid blue, OM dashed blue, YF solid red, OF dashed red; gray = dark phase. (**B**) Bootstrap mean-difference smooths (95% CI) for six marginal contrasts. Blue: Group 1 > Group 2; red: Group 1 < Group 2. Tier 3 exploratory; cluster boundaries in Supplementary Table S8; Tier 1 confirmatory endpoint (TDW fraction at ZT16) in Supplementary Figure S11.

All four groups exhibited the characteristic N-shaped temporal structure: a progressive rise in NREM during the late-dark phase, a sharp dip at the dark–light transition, and a sustained plateau during the light phase (Figure 3). Throughout, we use “N-shape” as a descriptive label for this three-landmark motif—a late-dark NREM rise (Peak 1), a pre-lights-on trough, and an early-light NREM peak (Peak 2)—without implying a specific generating mechanism.

Group-specific GAM fits captured 57–75% of variance in the 24 h NREM profile (R^2^: 0.57 [old male] to 0.75 [young female]). Bootstrap difference smooths with significant-run extraction revealed that group divergence localized mainly to the dark phase and the dark–light transition interval. Old mice showed higher NREM than young mice in the early-to-mid dark phase (both sexes), and males showed higher NREM than females in the late-dark and early-light phases, with the sex contrast most extensive in young animals. Exact cluster boundaries for all six contrasts are provided in Supplementary Table S8. These are marginal contrasts, not main effects from an integrated factorial model; confirmatory inference on the primary endpoint is provided in Section 3.2.3 below.

Leave-one-out jackknife confirmed that the divergence patterns were not driven by any single animal: 84% of ZT grid points retained significance with any one animal removed.

#### 3.2.2. Phase 2: Aging Preserves Ultradian Oscillation Frequency but Alters Amplitude Characteristics

Across the full 24 h cycle, animals exhibited 6.8–7.8 prominent NREM peaks (mean inter-peak interval, IPI: 2.6–3.4 h), with no significant difference by age (*p* = 0.76) or sex (*p* = 0.65) in total peak count. No significant group differences were detected in IPI CV (0.42–0.53, *p* > 0.19), indicating broadly preserved ultradian recurrence structure (Supplementary Figure S12; Supplementary Table S9).

The one exception among global ultradian metrics was weighted circular variance, which showed a nominal age effect (*p* = 0.040; Supplementary Table S9). We do not emphasize this result in our primary interpretation because weighted circular variance in the present framework conflates phase concentration with prominence weighting, and its biological meaning in isolation is ambiguous. It is reported here for completeness and warrants targeted investigation in larger samples.

Despite similar oscillation frequency, amplitude characteristics differed by circadian phase (Supplementary Figure S13; Tier 3 exploratory). In the late-dark phase (ZT18–ZT0), males showed higher peak amplitudes than females (65% vs. 56%, nominal *p* = 0.034), and this difference was also present in the early-light phase (76% vs. 71%, nominal *p* = 0.007). Trough depth showed a nominal age effect in the early-dark phase (ZT12–ZT18): old mice had mean trough NREM of 19% compared to 6% in young mice (nominal *p* = 0.002).

The Phase 2 finding with the largest age contrast was the TDW fraction at troughs, which showed a strong dark-phase-specific age contrast (Supplementary Figure S11). In the early-dark phase, young mice had a TDW fraction of 0.68 compared to 0.45 in old mice (nominal *p* < 0.001); in the late-dark phase, it was 0.63 vs. 0.47 (nominal *p* = 0.026). This age gap was not detected in the light phase (0.19–0.37, *p* > 0.18). The oscillation range showed no significant group differences in any phase (all *p* > 0.30), indicating that the detected age contrast on trough depth was stronger than any effect on range. These phase-binned findings motivated the pre-specified Tier 1 test on TDW fraction at the N-shape onset (ZT16), as well as a complementary Tier 2 characterization at the data-driven trough, both reported in Section 3.2.3.

#### 3.2.3. Phase 3: N-Shape Landmarks Reveal Dissociable Age and Sex Effects on Amplitude and TDW Enrichment

Tier 1 confirmatory test: In a factorial age × sex ANOVA on TDW fraction at ZT16 (*n* = 24), the age main effect was highly significant (F(1, 20) = 26.94, *p* < 0.0001, Hedges’ g = 2.05, 95% CI [1.14, 2.97]), with no sex main effect (F(1, 20) = 0.76, *p* = 0.392) and no age × sex interaction (F(1, 20) = 1.12, *p* = 0.303; Supplementary Table S10, Panels B and C). Young mice had a TDW fraction of 0.628 ± 0.083 at ZT16, compared to 0.392 ± 0.133 in old mice—a 37.6% relative reduction. Because ZT16 is fixed in advance of data inspection, this endpoint is robust to the selection bias that attends landmark-based analyses, and because it is sampled at a fixed bin it does not depend on peak-detection smoothing or prominence parameters.

Tier 2 landmark analysis: Within the predefined ZT16–ZT6 window, Peak 1 occurred at ZT19.0–ZT20.6 across groups, with no Holm-corrected difference by age (*p* = 1.00) or sex (*p* = 0.18; Supplementary Figure S11). No Holm-corrected differences were detected in time to Peak 1 or FWHM. Peak 1 ZT showed poor stability across preprocessing variants (ICC = 0.30) and FWHM showed poor stability (ICC = 0.48); we therefore explicitly do not interpret Peak 1 timing or FWHM as inferential anchors.

TDW fraction at the data-driven trough was the only Tier 2 landmark to survive Holm–Bonferroni correction (*p* = 0.004, Cohen’s d = 1.43, 95% CI [0.68, 2.52]; Supplementary Figure S11; Supplementary Table S11). Young mice showed a TDW fraction of 0.70 ± 0.11 at their individual trough locations, compared to 0.52 ± 0.14 in old mice (F(1, 20) = 12.58, *p* = 0.002). This metric was the most robust across preprocessing variants among the landmark-based measures (ICC = 0.91). The trough analysis complements the Tier 1 ZT16 result by characterizing where within the ZT20–ZT2 window the age-related TDW suppression is maximally expressed; the effect size at the trough (d = 1.43) is smaller than at ZT16 (g = 2.05) but in the same direction, and the agreement of the two endpoints on the direction and significance of the age effect strengthens confidence that the observed TDW reorganization is not an artifact of the landmark-selection procedure.

Peak 1 amplitude showed a nominal sex effect (*p* = 0.035, d = 1.04 [0.34, 1.98]) that did not survive correction: males reached 68% NREM at Peak 1 compared to 59% in females. This metric was stable across preprocessing (ICC = 0.86). Among exploratory landmarks (Supplementary Table S12; Tier 3), after correcting the circular time handling for trough ZT, a significant sex effect on trough timing emerged: males’ troughs occurred ~1.1 h later than females’ (*p* = 0.001, d = 1.66 on the linear “hours after ZT20” representation); this effect was not detected under the original linear-averaging analysis. The NREM–TDW Pearson correlation showed a significant age effect (*p* < 0.001, d = −1.22): the inverse relationship between NREM and TDW weakened from r = −0.94 in young to r = −0.88 in old mice. Peak 2 amplitude showed a nominal sex effect (*p* = 0.026, d = 1.17). Descriptive landmarks (trough amplitude, ΔNREM, slopes, Peak 2 ZT) are reported without hypothesis testing in Supplementary Table S12.

#### 3.2.4. Phase 4: The Pre-Lights-On Trough Is a Wake-Dominated Transition Whose TDW Enrichment Declines with Aging

State composition analysis at the three N-shape landmarks showed predominantly NREM at Peak 1 and Peak 2 (60–81%) with modest REM (5–12%), while the trough was almost entirely wake (NREM 6–12%, REM < 1%; Supplementary Figure S14). The composition of wake at the trough, however, differed markedly by age. Pooled across sex, young mice showed a TDW fraction of 0.70 ± 0.04, compared to 0.52 ± 0.05 in old mice (d = 1.43). This decline reflected both reduced absolute TDW (young 64% → old 48%, nominal *p* = 0.019, d = 1.09) and elevated absolute nTDW (young 26% → old 43%, nominal *p* = 0.003, d = −1.53).

Because wake microcomposition is compositional, this effect was also tested with a binomial generalized linear model on TDW counts out of total wake counts (Supplementary Table S13). Aging reduced the odds of TDW (versus nTDW) at the trough approximately three-fold (OR = 0.32, *p* < 0.0001). A significant age × sex interaction also emerged (OR = 2.03, *p* = 0.0002); post hoc contrasts showed that the age-related TDW reduction was larger in males (OR = 0.32, *p* < 0.001) than in females (OR = 0.64, *p* < 0.001). This interaction was model-dependent—null in the per-animal ANOVA (*p* = 0.156), borderline in the daily level mixed model (*p* = 0.067), and significant only in the binomial model, which uses count-level data with wake-denominator information. The age main effect on trough TDW fraction was consistent across all three models; sex-dependent modulation should be interpreted cautiously pending replication.

The TDW fraction trajectory across the five pre-specified fixed ZT reference points (Supplementary Figure S15, Supplementary Table S10) revealed that the age gap was not confined to the data-driven trough but extended across the dark phase, with the effect strongest at the N-shape onset (ZT16: young 0.628, old 0.392; F(1, 20) = 26.94, *p* < 0.0001, g = 2.05—the Tier 1 primary endpoint) and intermediate at Peak 1 (young 0.41, old 0.28, *p* = 0.035). By ZT3, the direction had reversed (old 0.343 > young 0.255, *p* = 0.024, d = −1.04), indicating that the age-related TDW redistribution is temporally structured across the N-shape. This trajectory converges with the Tier 2 data-driven trough result (F = 12.58, *p* = 0.002, d = 1.43; Section 3.2.3). After lights-on, the age gap narrowed, and by Peak 2 and beyond, group differences in TDW fraction were no longer detected (0.18–0.37 across groups).

#### 3.2.5. Sensitivity Analyses and Day-Level Validation Confirm Robustness of Primary Findings

Parameter sensitivity analysis across nine smoothing/prominence combinations showed high stability for TDW fraction at trough (ICC = 0.91) and Peak 1 amplitude (ICC = 0.86; Supplementary Figure S16; Supplementary Table S14). Peak 1 ZT was more sensitive (ICC = 0.30), reflecting the broad morphology of the late-dark NREM peak. Exclusion of Day 0 preserved the primary TDW fraction age effect (*p* = 0.023). Additional robustness checks—including leave-one-out jackknife on the Phase 1 GAM, linear day-slope analysis, and two-harmonic cosinor bimodality of ZT16–ZT22—are provided in Supplementary Results; none altered the primary finding.

Day-level validation of the primary endpoint: To directly address whether the primary age effect could be an artifact of 14-day averaging, the ZT16 TDW fraction was re-extracted independently for each of the 14 recording days and each of 24 animals (320 animal-day observations after excluding 16 animal-days with zero wake epochs in the ZT16 bin) and analyzed with a mixed-effects model (ZT16 TDW fraction ~ age × sex + (1|Mouse); Supplementary Table S15, Panel A). The age main effect was significant at the day level (β = −0.229, SE = 0.060, *p* < 0.001), with a day-drift coefficient of −0.002/day (*p* = 0.68, estimated in a separate additive-day model) indicating no progressive drift across the 14-day window. The animal-level ICC was 0.06, indicating that most day-to-day variance at this single 15 min ZT bin reflects within-animal fluctuation rather than stable between-animal differences. Day-by-day inspection showed that the young > old TDW-fraction contrast at ZT16 was present on 13 of 14 recording days. An equivalent day-level validation at the data-driven trough (Tier 2 supporting; 336 animal-day observations) yielded a directionally consistent age contrast (β = −0.200, SE = 0.085, *p* = 0.019; 12 of 14 days young > old; Supplementary Table S15, Panel B). Together, these day-level results argue against an averaging artifact at both the Tier 1 primary (ZT16) and Tier 2 descriptive (trough) endpoints, and indicate that the age-related TDW reduction is already present at the single-day level; 14-day averaging reduces within-animal noise to produce cleaner group estimates.

### 3.3. Episode Architecture and Bout-Length Distributions

#### 3.3.1. Aging Shortens Theta-Dominated Wake Bouts and Increases Their Frequency

**Figure 4:**
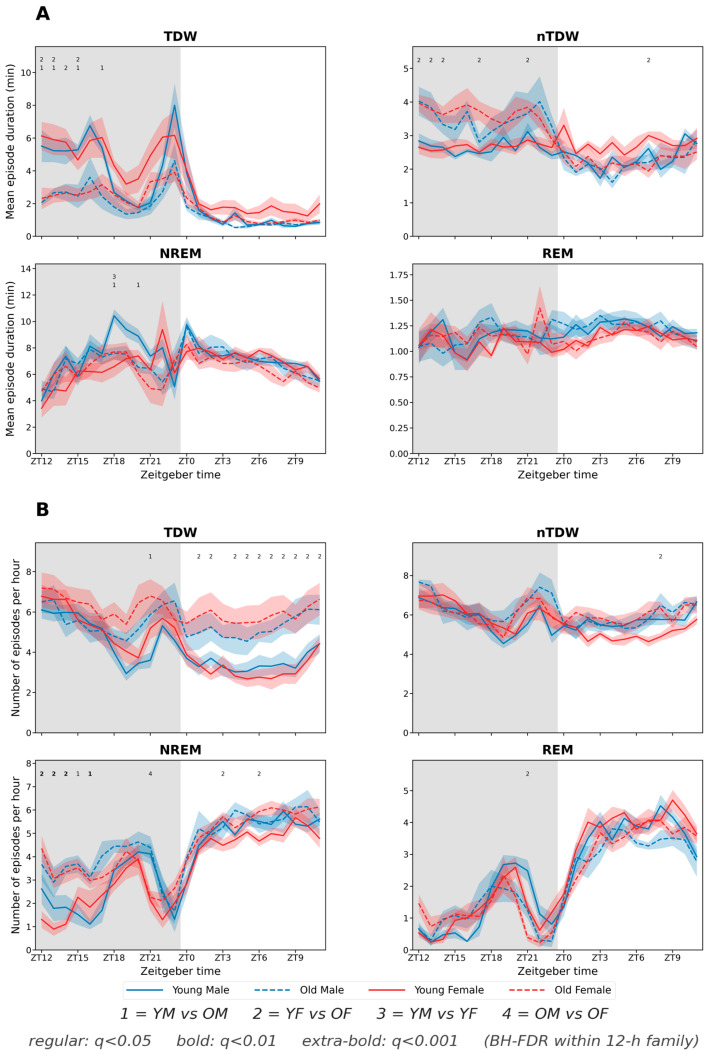
Aging shortens dark-phase TDW bouts, lengthens nTDW bouts, and increases NREM episode count; REM episode architecture is preserved. (**A**) Hourly mean episode duration (min) and (**B**) hourly episode count, computed from 10 s epoch run-length encoding in 1 h bins, per-mouse 14-day means. Shading: ± SEM. Gray shading: dark phase. Numeric pair-code significance markers above each ZT bin indicate Tier 3 (exploratory) cascade-gated hourly age contrasts; font weight encodes significance: regular *p* < 0.05, bold *p* < 0.01, extra-bold *p* < 0.001 (all Holm-corrected within each 12 h phase family). Phase-level summary in Supplementary Figure S17. *n* = 6/group.

Old mice of both sexes exhibited markedly shorter TDW bouts during the dark phase (Figure 4). Old males had TDW bouts 2.17 min shorter than young males (YM: 4.55 ± 0.39; OM: 2.37 ± 0.42 min; Hedges’ g = 2.04 [1.03, 4.88]; *p*Holm = 0.037; full contrast table in the master supplement). The collapsed young-versus-old dark-phase contrast was the strongest effect in the entire episode architecture dataset (Δ = 2.28 min; g = 2.10 [1.37, 3.47]; *p*Holm < 0.001). In the light phase, all groups showed uniformly short TDW bouts (0.93–2.04 min), with no significant group differences. The within-group dark–light TDW bout difference was significant in all groups (all *p*Holm < 0.02), but was substantially attenuated in old mice (old: Δ = +1.44 to +1.49 min vs. young: Δ = +2.93 to +3.35 min) (Supplementary Figure S17).

TDW episode count showed a complementary pattern: old mice produced more TDW episodes during the light phase (collapsed Δ = −28.6 episodes/12 h; *g* = −1.60 [−2.90, −0.89]; *p*Holm = 0.006). The omnibus LMM confirmed a significant age × hour interaction (LRT χ^2^(23) = 143.9, *p* < 0.0001). Together, these findings are consistent with a shift from consolidated dark-phase TDW toward shorter bouts, accompanied by more frequent TDW intrusions during the light phase—a pattern suggestive of reduced TDW consolidation and altered circadian wake organization.

#### 3.3.2. Aging Lengthens Non-Theta-Dominated Wake Bouts and Increases NREM Episode Number

In a pattern that mirrors the TDW shortening, old mice had significantly longer dark-phase nTDW bouts (old: 3.29–3.42 min vs. young: 2.56–2.58 min; collapsed Δ = −0.78 min; g = −1.45 [−2.38, −0.94]; *p*Holm = 0.016). Cascade-gated hourly analysis localized the age effect to ZT12–ZT16. nTDW episode count did not differ between groups, indicating that the nTDW elongation reflects sustained drowsy episodes rather than more frequent nTDW entries. NREM bout duration tended to be shorter in old mice during the dark phase but did not survive Holm correction (g = 1.10; *p*Holm = 0.052). However, NREM episode count showed a clear age effect: old mice had more dark-phase NREM episodes (collapsed Δ = −14.0; g = −1.46 [−2.95, −0.67]; *p*Holm = 0.015). REM episode architecture was preserved with aging: no between-group contrasts survived correction. Only one sex contrast survived Holm: young females had longer light-phase nTDW bouts than young males (Δ = −0.49 min; g = −1.96; *p*Holm = 0.046). Phase-level summary in Supplementary Figure S17.

#### 3.3.3. Vigilance-State Redistribution Confirmed at the Bout Level

Two-way ANOVA on state time recapitulated the redistribution pattern established in Section 3.1 at the bout level. No age × sex interactions were detected for any state. During the dark phase, old mice spent less time in TDW (F(1, 20) = 52.35, q < 0.001, η^2^ = 0.67) and more time in nTDW (q = 0.004) and NREM (q = 0.012). This confirms that the bout-architecture changes described below operate on the same redistributed total-time substrate characterized in Section 3.1.

#### 3.3.4. Survival Analysis Reveals State-Specific Aging Effects on Bout Stability

Cox proportional hazards modeling provided a continuous-time descriptive convergence test on state stability. Because Schoenfeld residuals detected proportional-hazards violations that phase stratification alone cannot fully resolve, and because animal-level replication remains bounded at *n* = 24, episode-level Cox results are reported here as convergent supportive evidence rather than as independent confirmatory inference. Among the four vigilance states, TDW showed the strongest age effect: old mice exited TDW at a 61% higher rate than young mice (hazard ratio [HR] = 1.61, 95% CI: 1.33–1.95, *p* < 0.0001). Phase-stratified analysis revealed a dark-phase age HR of 1.78 (*p* < 0.0001) and a light-phase age HR of 1.45, with an additional light-phase sex effect (HR = 1.49, *p* < 0.001) and a modest but significant age × sex interaction (HR = 0.75, *p* = 0.036), indicating that the age-related acceleration of TDW exit was more pronounced in males than in females during the light phase.

nTDW exhibited a striking circadian reversal: dark-phase HR = 0.74 (*p* < 0.001), meaning old mice stayed in nTDW longer during the dark phase; light-phase HR = 1.21 (*p* < 0.001), meaning old mice exited nTDW faster during the light phase. This reversal is compatible with aged mice settling into prolonged quiet drowsiness during the active phase, while light-phase nTDW shortening is compatible with increased homeostatic sleep drive during the inactive phase—though direct attribution to sleep pressure would require independent measurement of homeostatic proxies such as delta power. NREM showed no significant survival effects (Age HR = 1.13, *p* = 0.22), and REM was unaffected by aging, with only a modest dark-phase sex effect (HR = 0.84, *p* = 0.011). Per-mouse Cox models with cosinor harmonics alone (cos_ZT, sin_ZT coefficients extracted as per-animal summary statistics of circadian bout stability; all 24 mice converged) confirmed the age effect at the animal level (age effect on nTDW sin_ZT: F(1, 20) = 29.59, *p* < 0.001; age effect on TDW sin_ZT: F(1, 20) = 5.22, *p* = 0.033). This validation was added post hoc to place survival inference on the same *n* = 6 per cell replication scale as the primary phase-level contrasts, and reduces concern that the episode-level Cox effects are solely driven by episode-count pseudoreplication.

#### 3.3.5. Mixture Decomposition Reveals Remodeling of TDW Bout Architecture

Bi-exponential mixture models decomposed the TDW bout-length distribution into short-bout and long-bout components (Supplementary Table S16; Supplementary Figure S18, panel A). Mixtures were fit independently per mouse, and animal-level ANOVAs were computed on the extracted parameters (*n* = 6 per cell). BIC favored 2-component mixture fits for TDW in 79% of mice in the dark phase and 100% of mice in the light phase, and 1-component fits for NREM and REM in all mice. Consistent with this single-component architecture, NREM and REM episode-count distributions showed an age-related increase in NREM episode number and largely preserved REM episode architecture (Supplementary Figure S19). The two-component structure was therefore robust in the light phase and prevalent, though not universal, in the dark phase; interpretation of the dark-phase mixture parameters in old males should accordingly be treated with caution. Aging collapsed both the short-bout and long-bout components in dark-phase TDW. The complementary nTDW analysis (Supplementary Figure S20) showed lengthening of the dark-phase bout distribution, consistent with a shift from consolidated TDW toward drowsier nTDW wake.

### 3.4. NREM Sleep Transition Dynamics

#### 3.4.1. NREM → Wake Transitions Are Elevated in Aged Mice Across Sexes and Phases

**Figure 5:**
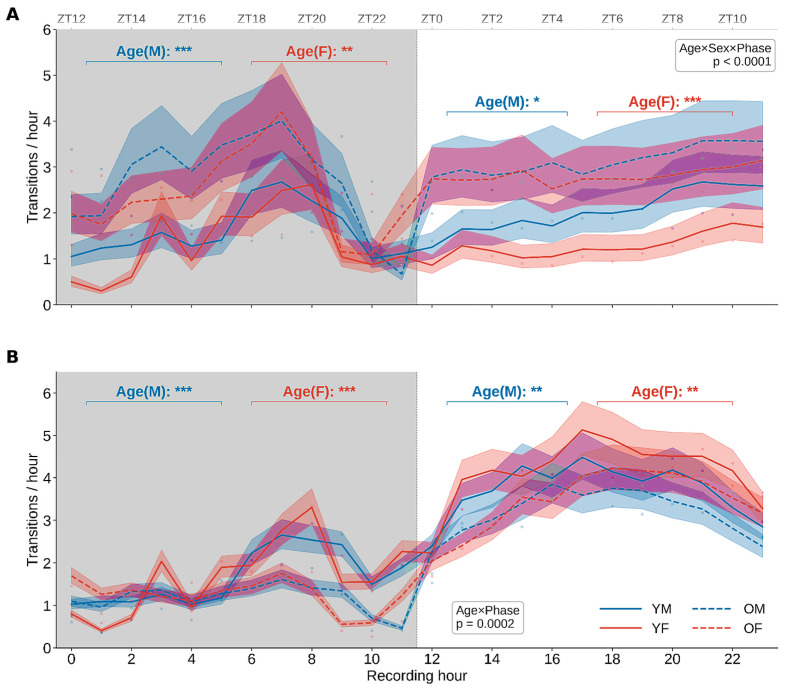
Aging shifts NREM exit dynamics away from REM and toward wakefulness, with phase- and sex-dependent modulation. NB GLMM-predicted transitions per hour with log(NREM epochs) offset (rates per unit NREM opportunity, not raw counts). (**A**) NREM → wake. (**B**) NREM → REM. Lines + shaded 95% CIs; faint points: observed means. Group coding as in Figure 3; gray shading = dark phase. Significance bars: planned Holm-adjusted age contrasts within sex × phase shown as horizontal brackets labeled by sex (blue = male, age (M); red = female, age (F)); asterisk convention * *p* < 0.05, ** *p* < 0.01, *** *p* < 0.001. Omnibus interactions—NREM → wake: age × sex × phase *p* < 10^−4^; NREM → REM: age × phase *p* = 2 × 10^−4^. *n* = 6/group.

The NB GLMM revealed a significant three-way age × sex × phase interaction (χ^2^(1) = 34.6, *p* < 0.0001; Figure 5 and Supplementary Figure S21; Supplementary Tables S17–S18), indicating that the effect of aging on the NREM → wake transition rates differed by sex and circadian phase. Because the model included log(NREM epochs) as an offset, this effect is not attributable to differences in NREM quantity; it reflects altered exit dynamics from NREM itself. Decomposition of the three-way interaction is presented in the phase-resolved contrasts below.

Model-predicted transition rates revealed that the age-related increase was most pronounced in females during the light phase (Old/Young RR = 2.11, 95% CI [1.58, 2.83]; *p* < 0.0001), compared to a 1.45-fold increase in males (RR = 1.45, [1.07, 1.96]; *p* = 0.016). During the dark phase, aging increased rates 1.73-fold in males (*p* = 0.0005) and 1.67-fold in females (*p* = 0.002). A sex difference in light-phase rates was present in young mice (female/male RR = 0.67; *p* = 0.008) but abolished in old mice (RR = 0.98; *p* = 0.89, Supplementary Table S18), indicating convergence of fragmentation patterns with aging—a finding that parallels the convergence of vigilance-state percentages observed in Section 3.1.

A caveat is warranted: although the three-way interaction was statistically significant, biological replication was limited to *n* = 6 per cell, and effect-size estimates for higher-order interactions may be inflated in small samples. The cell-specific rate ratios reported above should therefore be interpreted as provisional estimates that require replication in larger cohorts (*n* ≥ 10–12 per cell) to establish their precise magnitude.

The cosinor model provided a superior fit to the full 24 h profile (ΔAIC = 108.1) and indicated group differences in the rhythmic organization of NREM → wake transitions. Young females showed the largest estimated circadian amplitude, and old females the smallest, consistent with attenuation of daily modulation in aged females. These harmonic estimates should be treated as compact descriptors of rhythmic structure rather than mechanistic parameters, because hourly transition profiles are not strictly sinusoidal; phase (dark/light) is therefore retained as the primary basis for inference.

#### 3.4.2. NREM → REM Transitions Are Reduced in Aged Mice with Phase-Dependent Modulation

NREM → REM transitions displayed a robust circadian pattern, with markedly higher rates during the light phase. The NB GLMM revealed no three-way age × sex × phase interaction (*p* = 0.54) and no age × sex interaction (*p* = 0.91), but a significant age × phase interaction (χ^2^(1) = 14.2, *p* = 0.0002; Supplementary Table S19), indicating that the age-related reduction in NREM → REM transitions differed between dark and light phases. A significant sex main effect was also present (χ^2^(1) = 8.72, *p* = 0.003), indicating consistently higher NREM → REM rates in females regardless of age or phase.

Decomposition of the age × phase interaction showed that old mice had lower NREM → REM rates in both phases, with the reduction approximately twice as large during the dark phase (old/young RR = 0.71 males, 0.67 females; both *p* < 0.0001) compared to the light phase (RR = 0.85 and 0.84; both *p* < 0.01). Because the model included NREM exposure as an offset, this result indicates that aging reduced REM-directed exit from NREM per unit NREM opportunity, rather than merely reflecting reduced total sleep time. Females had consistently higher NREM → REM rates than males across ages and phases (female/male RR = 1.17–1.23, all *p* < 0.03), but this sex effect was small relative to the age and phase dependence of the response. The light-to-dark ratio was greater in old mice (1.67–1.69) than young (1.35–1.40), reflecting disproportionate dark-phase REM loss with aging.

A supplementary binomial GLMM confirmed that aging also reduced the hourly probability that any NREM → REM transition occurred (age main effect χ^2^(1) = 5.27, *p* = 0.022; Supplementary Figure S22, Supplementary Table S20). Because the rate-based count model and the occurrence-based binomial model converged directionally, the reduction in NREM → REM transitions does not appear to reflect a simple threshold effect at very low transition frequencies; rather, the data support a broader age-related suppression of REM-directed transitions from NREM, with the rate effect being quantitatively dominant. We note that “REM gating” in this framework is an operational descriptor of transition permissibility at the behavioral-state level, and should not be read as direct evidence for a specific circuit-level REM gating mechanism.

Taken together, these analyses show that aging remodels NREM exit architecture in a destination-specific manner. When NREM occurs, old mice are more likely than young mice to exit NREM toward wake, but less likely to exit NREM toward REM. This bidirectional shift is compatible with increased instability of NREM maintenance together with reduced REM entry propensity, and it is modulated by circadian phase, with particularly strong suppression of NREM → REM transitions during the dark phase. The robust, sex- and phase-general finding is that aging shifts NREM exit dynamics away from REM and toward wake.

### 3.5. Ultradian Block Architecture

A total of 5333 valid ultradian blocks and 2828 out-block episodes were identified across 24 mice over 14 recording days. Block architecture showed strong circadian modulation, with shorter, more frequent blocks during the dark phase and longer, more consolidated blocks during the light phase.

**Figure 6:**
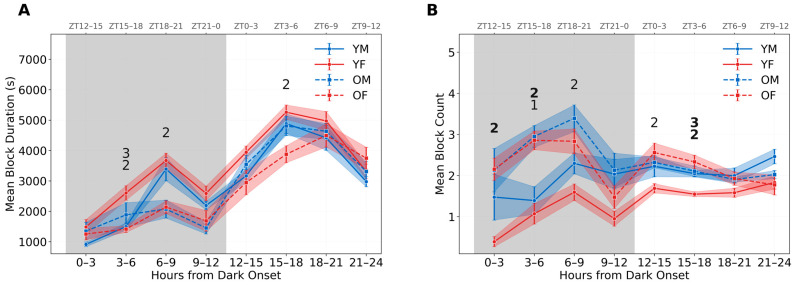
Ultradian blocks are shorter and more numerous in aged mice, with the age effect most pronounced in females. Block architecture per 3 h circadian bin (5333 blocks across 24 mice × 14 days). (**A**) Block duration (s); (**B**) block count. Lines: group means ± SEM. Group coding as in Figure 3; gray = dark, white = light phase. Numeric pair-code significance markers above each ZT bin (Tier 3, exploratory) (1 = YM vs. OM; 2 = YF vs. OF; 3 = YM vs. YF); font weight encodes significance: regular *p* < 0.05, bold *p* < 0.01, extra-bold *p* < 0.001. Gamma GLMM for duration; Poisson GLMM for count. *n* = 6/group.

Block duration peaked in the mid-light phase (ZT3–6) across all groups (Figure 6). The Gamma GLMM revealed significant age × bin (*p* < 0.001), sex × bin (*p* = 0.015), and age × sex × bin (*p* < 0.001) interactions; young females had longer dark-phase blocks than old females (Δ = 1204 s, d = 3.02, *p* = 0.002). Block count showed the inverse pattern (age: *p* = 0.001), indicating that aging fragments ultradian cycling into more numerous but shorter blocks. Per-mouse cosinor parameters and factorial ANOVAs are reported in Supplementary Tables S21 and S22.

Intra-block wake fraction was elevated in old mice (age: p = 0.004; age × sex × bin: *p* < 0.001), most prominently in light-phase old females compared to young females (d = −4.12, *p* < 0.001) (Supplementary Figure S23). NREM fraction declined modestly with circadian phase (sex effect: *p* = 0.007), and REM fraction was higher in young animals during the light phase. Poisson GEE modeling adjusted for total sleep duration confirmed elevated intra-block NREM → wake transition rates in aged light-phase blocks (age × early-light RR = 0.73, *p* = 0.025; age × late-light RR = 0.61, *p* < 0.001) (Supplementary Table S23).

Out-block episodes—isolated single-state sleep fragments too short and too separated from neighbors to qualify as ultradian blocks—were more frequent in old mice (age: *p* = 0.003; age × bin: *p* < 0.001), with a clear age gradient: old females 987 (34.9%), old males 780 (27.6%), young females 626 (22.1%), young males 435 (15.4%). Old males had significantly more dark-phase out-blocks than young males (d = −3.05, *p* = 0.004). These fragments clustered at short durations (<500 s) without circadian duration modulation, statistically separable from the short tail of valid blocks via the hurdle model (Supplementary Table S24, Supplementary Figure S24). Detailed analyses are provided in Supplementary Figures S24–S28 and Supplementary Tables S21–S24.

### 3.6. EEG Spectral Analysis

The spectral analysis addressed age- and sex-related effects at three hierarchical levels: (1) absolute whole-spectrum differences defined by cluster-based permutation testing (CBPT), providing the primary inferential backbone; (2) circadian temporal redistribution of spectral power assessed through hourly normalized analyses; and (3) secondary mechanistic decomposition through relative PSD, scalar band-power summaries, and spectral parameterization.

#### 3.6.1. Primary Whole-Spectrum Age and Sex Effects on Absolute PSD

Omnibus frequency-interaction tests supported the use of a frequency-resolved framework. Age × frequency interactions were significant in seven of eight state × phase combinations (all *p* < 0.001 except REM-light), and sex × frequency interactions reached significance in TDW, nTDW, and NREM in both phases and in REM during the dark phase, indicating that age and sex effects were generally frequency-dependent rather than uniform across the spectrum.

**Figure 7:**
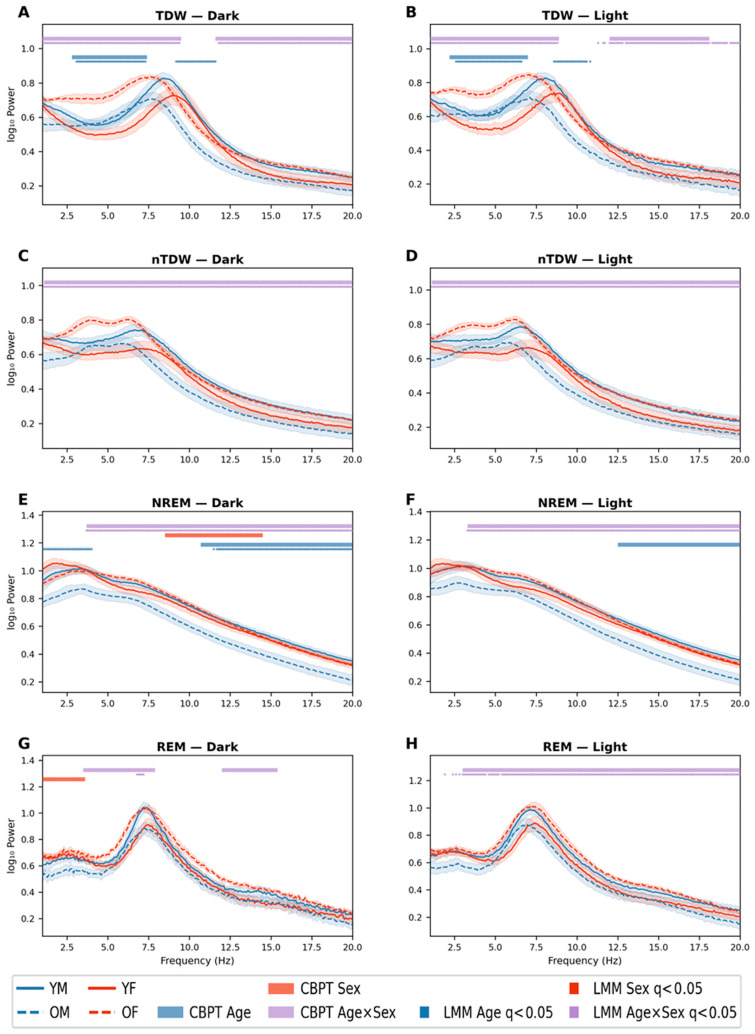
Age × Sex interaction spans the spectrum in wake states; age main effects localize to wake theta and NREM sigma/beta. Primary whole-spectrum inferential figure. Group-averaged log_10_ absolute PSD (µV^2^/Hz; 1–20 Hz, 0.1 Hz resolution; Welch, 4 s Hanning, 50% overlap) across four vigilance states × two phases, shown as (**A**) TDW, dark phase; (**B**) TDW, light phase; (**C**) nTDW, dark phase; (**D**) nTDW, light phase; (**E**) NREM, dark phase; (**F**) NREM, light phase; (**G**) REM, dark phase; (**H**) REM, light phase. Bars below panels: CBPT clusters (solid, p_FWER < 0.05); BH-FDR-significant bin-wise LM bins (dotted, q < 0.05). The CBPT clusters constitute the primary, family-wise-error-controlled whole-spectrum inference; the dotted bin-wise bars are a secondary, descriptive localization of where differences fall and should be interpreted accordingly. Blue = age; orange/salmon = sex; purple = age × sex. Color/bar convention used throughout the spectral figures. Cluster statistics: Supplementary Table S25. *n* = 6/group.

CBPT identified 15 significant clusters across the 24 state × phase × effect panels (Figure 7; Supplementary Table S25). The dominant whole-spectrum finding was a robust age × sex interaction producing significant clusters in all eight state × phase combinations: TDW interaction clusters spanned 1.0–9.5 Hz and 11.7–20.0 Hz (dark) and 1.0–9.1 Hz and 11.2–20.0 Hz (light); nTDW clusters spanned essentially the entire analyzed range in both phases; NREM clusters extended broadly above 3.7 Hz (dark) and 3.3 Hz (light); REM clusters were segmented in the dark phase but broader (2.8–20.0 Hz) in the light phase. The age- and sex-divergent spectral consequences thus extended across wake and sleep states.

Age main effects were more selective, with TDW theta-range clusters (dark: 2.8–7.6 Hz; light: 2.1–7.1 Hz) indicating an age-related reduction in wake theta power, and NREM clusters confined to higher frequencies (dark: 10.2–20.0 Hz; light: 11.9–20.0 Hz) indicating attenuation of sigma and higher-frequency NREM power. No age clusters reached FWER significance in nTDW or REM, and sex main effects produced no CBPT-significant clusters in any state × phase combination. Bin-wise effect-size profiles broadly supported the CBPT-defined effects (Supplementary Figures S29–S31), with age effects on absolute PSD reaching |Cohen’s d| > 0.8 in wake-state theta bands and in NREM sigma (Supplementary Figure S30).

#### 3.6.2. Circadian Temporal Organization of Spectral Differences

**Figure 8:**
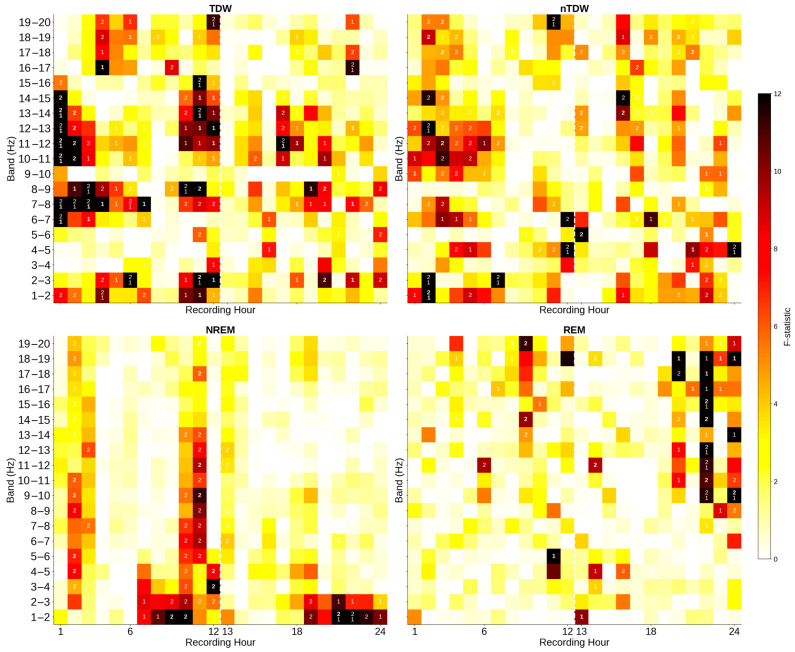
Age-related spectral redistribution concentrates at dark-phase onset (ZT12–ZT13), with strongest effects in TDW sigma/alpha (10–13 Hz) and nTDW low frequencies (1–2 Hz). F-statistic heatmap for the age main effect from 2 × 2 age × sex ANOVA per state × band × hour cell (19 bands × 24 h × 4 states = 1824 cells), 24 h normalization. Bands span 1–20 Hz in 1 Hz steps. Color encodes F-statistic magnitude; cells passing FDR *q* < 0.05 are outlined. The largest age effects cluster in the early-dark phase (ZT12–ZT15) in TDW (10–13 Hz) and nTDW (1–2 Hz). In-cell numerals are compact pair-code localization aids indicating which pairwise group contrast drives a given cell: 1 = YM vs. OM (age effect in males), 2 = YF vs. OF (age effect in females); font weight encodes significance level (bold = *p* < 0.01, regular = *p* < 0.05). These codes identify which contrast contributes to each cell rather than reporting exact statistics; exact F-values and effect sizes are tabulated in the Supplementary Materials. This panel is a secondary, descriptive localization analysis. See Supplementary Table S27 for top 20 cells by partial η^2^_p.

Under the primary 24 h normalized framework, aging was the dominant source of variation in temporal spectral redistribution. Across the 1824 state × band × hour cells, age main effects reached nominal significance in 373 cells (75 surviving FDR), versus 203 cells (four surviving) for sex and 104 cells (one surviving) for age × sex. The strongest age effects concentrated in the early-dark phase, particularly in wakefulness-associated states (Figure 8). The largest single effect occurred at TDW 11–12 Hz at recording hour 1 (F = 47.4, *p* = 1 × 10^−6^, η^2^p = 0.70), with closely related effects spanning the 10–13 Hz range at dark-phase onset; young mice showed positive normalized values at this time and old mice values near zero, indicating attenuation of the early-dark sigma/high-alpha peak (Supplementary Figure S32). In nTDW the dominant age effect was a 1–2 Hz attenuation at ZT13 (Supplementary Figure S33). In NREM, age-related changes concentrated in late-dark delta (3–4 Hz) and early-light low frequencies (1–2 Hz) (Supplementary Figure S34). REM showed broad but low-amplitude effects with higher data sparsity (Supplementary Figure S35). Sex and age × sex effects on normalized spectral power were weaker and concentrated mainly in TDW 4–6 Hz dark-phase and in REM. Partial-η^2^ magnitudes confirmed that the age effect was concentrated in early-dark TDW sigma/high-alpha (η^2^p up to 0.70) and in nTDW 1–2 Hz at ZT13 (η^2^p up to 0.65) (Supplementary Figure S36). These age effect localizations were robust to the choice of normalization reference: the convergence map across the three normalization frameworks (24 h, phase-matched, ZT8–11 anchored) identified four core age effect loci in TDW dark phase (ZT12, 10–13 Hz) and one in the nTDW dark phase (ZT13, 1–2 Hz) that surfaced in the top-20 partial-η^2^ ranking under all three frames (Supplementary Figures S37–S41 and Supplementary Table S26).

#### 3.6.3. Secondary Analyses

Secondary analyses provided interpretive context for the primary findings. Relative PSD showed widespread age-related shifts in proportional spectral allocation in waking states (FDR-significant in 168/191 TDW-dark and 175/191 nTDW-dark bins) but more restricted age effects in NREM (60/191 dark; 96/191 light), suggesting that the NREM absolute-power loss at higher frequencies partly reflects broadband scaling rather than frequency-selective reshaping (Supplementary Figure S31). Scalar band-power confirmed age-related declines in NREM delta (dark: q = 0.004; light: q = 0.014) and a stronger age-related reduction in NREM sigma (both phases q < 0.001) directly matching the primary CBPT clusters; TDW theta-centroid frequency was robustly lower in old mice in both phases, indicating age-related slowing of the dominant waking theta rhythm (Supplementary Table S28). Spectral parameterization (FOOOF) confirmed this slowing as a reduction in theta center frequency in TDW, nTDW, and NREM, with NREM theta-peak power increased and bandwidth narrowed; aperiodic exponent showed no FDR-significant age or sex effects, while aperiodic offset showed an age × sex interaction in TDW (Supplementary Figure S42, Supplementary Table S29). Together these analyses indicate that the primary spectral findings reflect a combination of broadband amplitude changes and state-specific remodeling of theta-related periodic structure.

## 4. Discussion

This study provides a multi-level characterization of sleep–wake architecture in young and old C57BL/6J mice of both sexes, using 14 days of continuous EEG/EMG recording spanning circadian vigilance-state profiles, ultradian dynamics, episode architecture, bout-length distributions, state-transition kinetics, ultradian block organization, and EEG spectral composition. A key strength of this study is the prolonged recording duration for sleep–wake architecture. Relative to 1–3-day recordings typical of rodent sleep studies, this extended sampling captures day-to-day variability, improves the stability of circadian, fragmentation, transition, and block-based metrics, and reduces distortion from estrous-related fluctuations by averaging across multiple cycles without invasive staging. It also reveals temporal features that are often missed in short recordings, including reproducible age-related degradation of dark-phase wake quality, altered NREM exit architecture, and accumulation of isolated short sleep fragments. The extended dataset further enables variance decomposition and power analysis, clarifying which measures are intrinsically stable and whether increasing recording duration or sample size yields greater statistical power. Spectral analyses, however, were performed on a 72 h subset, and should be interpreted within that window.

Across analytical levels, age effects were substantially larger than sex effects and consistently expressed in five domains: (1) a pronounced dark-phase reduction in TDW, with redistribution toward nTDW and NREM sleep; (2) shortened and fragmented TDW bouts, with remodeled bi-exponential structure; (3) increased NREM → wake transition, indicating reduced sleep stability; (4) reduced NREM → REM transition, indicating impaired REM recruitment; and (5) altered ultradian block organization, with increased out-block episodes and higher within-block wake. The magnitude, timing, and state specificity of these effects were strongly circadian-phase dependent. The sex effects were detectable but largely underpowered at *n* = 6 per group and should be considered hypothesis-generating pending replication in larger cohorts.

### 4.1. Interpretive Framework: Age Differences as Candidate Markers of Aging

Age-related differences in sleep metrics were interpreted relative to young mice as a reference for normative sleep organization. However, the present cross-sectional design cannot distinguish whether these differences reflect normative aging or age-associated pathology, or compensatory adaptations. Resolving these alternatives will require longitudinal within-animal studies across the lifespan and additional pathophysiological and molecular analyses. Although aged mice in this study appeared healthy, they may still harbor unrecognized metabolic, inflammatory, neurodegenerative, or other neuropathological conditions that influence sleep. The observed phenotypes should therefore be interpreted with these limitations in mind.

### 4.2. Empirically Supported Findings

#### Circadian Structure and the Consequences of Temporal Resolution

Overall, 24 h sleep–wake architecture was preserved across all four groups, with nocturnal wake dominance and a light-phase sleep plateau consistent with established rodent patterns under 12:12 light–dark cycles [6,9,10]. However, group differences were highly time-localized. Whole-profile GAM analysis (Section 3.2) showed that age-related NREM divergence was concentrated mainly in the early-to-mid dark phase (ZT12–ZT18) while sex effects appeared mainly in the late-dark through early-light phase (ZT18–ZT6). These localized effects would be diluted or missed by conventional 12 h phase averaging, likely contributing to the limited sensitivity and inconsistent findings of studies based on coarse temporal bins.

The late-dark/early-light interval showed the most pronounced dynamics, including the characteristic N-shaped NREM profile: a late-dark rise, a sharp pre-lights-on dip, and an early-light-phase peak. The transient pre-lights-on increase in wake seems reminiscent of the human wake-maintenance zone (WMZ) [24,25,26], though any comparison should remain cautious due to differences in circadian context and operational definition. Our data support a late-dark interval of increased wake drive and reduced sleep continuity, consistent with circadian gating of arousal in rodents, but does not establish direct homology with the human WMZ, which differs in both circadian context and definition [8,24,27].

### 4.3. Integrative Interpretation and Candidate Mechanisms

#### 4.3.1. Mechanistic Interpretation of the N-Shape Dynamics

The three landmarks of the N-shape, the late-dark NREM rise (Peak 1), the pre-lights-on dip with its TDW surge (trough), and the early-light NREM surge (Peak 2), occupy a narrow ZT18–ZT3 sub-interval within the broader ZT16–ZT6 analysis window. Across this ~6 h span, vigilance-state composition undergoes a triple reversal: from active-phase wake dominance, to NREM levels approaching those of the mid-light phase, to a TDW-dominated arousal surge matching or exceeding early-dark wake, and finally back to early-light NREM-dominance. The classic two-process framework [27,28] was not designed to adjudicate such fine-scale transition-zone events in polyphasic rodents. The density and speed of these state reversals underscore the need for modeling approaches beyond monotonic Process S and sinusoidal Process C. Accordingly, the mechanistic interpretations proposed below should be viewed as candidate explanations rather than definitive conclusions supported by the present dataset.

Although first defined here in vigilance-state composition, the N-shape emerges as a recurring structural signature of the dark-to-light transition across six of the seven analytical domains of this study: the hour-by-hour vigilance-state profile (Section 3.1); the 24 h NREM temporal architecture (Section 3.2); episode and bout-length distributions (Section 3.3); NREM transition dynamics (Section 3.4); ultradian block architecture (Section 3.5); and EEG spectral architecture (Section 3.6). Across these analyses, the same ZT18–ZT3 sub-interval consistently exhibits the corresponding landmark signature, with stable Peak 1, trough, and Peak 2 ZT positions. The convergence of six independent analytical perspectives on the same temporal landmark structure indicates that the N-shape is not an artifact of any analytical method but a fundamental feature of sleep–wake reorganization across the dark-to-light transition.

The late-dark NREM rise (Peak 1) aligns most naturally with homeostatic interpretation. As small nocturnal mammals, mice engage in dense early-dark-phase activity across the first ~6 h after dark onset (ZT12–ZT18), generating sufficient sleep pressure via a short Process S cycle nested within the polyphasic dark phase. This pressure drives the late-dark NREM rise through adenosinergic accumulation [29,30] coincident with the Φ1 → Φ2 transition of the bimodal dark-phase structure [31] and declining orexinergic tone [32]. Under this interpretation, Peak 1 is largely homeostatic, with Process C contributing permissively by reducing wake-promoting opposition as its dark-phase amplitude wanes. However, the contributions of an active circadian component cannot be excluded; fluorescent calcium imaging in hypothalamic slices and in freely behaving mice shows that the SCN output carries a superimposed ultradian structure (0.5–4 h) originating in the SPZ–PVN region [33,34], making an active sub-24 h suprachiasmatic contribution mechanistically plausible. Distinguishing permissive from active circadian roles requires experimental dissociation (e.g., SCN lesion, T-cycle, or forced desynchrony protocols), which is beyond the scope of this study.

The trough is the most mechanistically ambiguous landmark. Its compositional profile—TDW-dominant in young mice (64% TDW vs. 26% nTDW) and more evenly split in aged mice (48% vs. 43%; Section 3.2.4)—is compatible with two non-exclusive accounts: a transient circadian arousal signal briefly outpacing sleep pressure, analogous in function to the human WMZ [24,35,36] and supported by SCN → DMH → LC pathways [37] and late-night orexin re-recruitment [32], or state instability near the homeostatic upper threshold without active arousal drive. The TDW-dominant composition is more consistent with active arousal, though instability at high sleep pressure could also recruit high-theta wake. Trough timing is sex-modulated (males ~1.1 h later than females; *p* = 0.001, d = 1.66), weakly favoring the circadian-arousal account [38,39]. Distinguishing these mechanisms requires targeted experiments.

The early-light NREM surge (Peak 2) reflects the convergence of release from the trough-phase wake bout combined with the onset of rest-phase circadian signaling. Its sharpness, rather than a gradual settling into light-phase NREM, indicates that once the trough-phase TDW surge dissipates, the accumulated homeostatic sleep pressure becomes suddenly unopposed, while SCN output and photic masking shift toward rest-phase patterns. Peak 2 likely represents homeostatic rebound accumulated across both the active dark phase and the trough-phase TDW surge. Because Peak 1 likely only partially discharges accumulated Process S rather than resetting it, sufficient residual load remains to support a sharp early-light NREM rise once trough-phase arousal terminates. Thus, the magnitude and timing of Peak 2 depends jointly on prior wake load and phase-dependent circadian signal at lights-on.

Age × sex effects at the landmarks: The most striking age × sex signal within the N-shape occurred at the trough, where the age-related reduction in TDW probability was larger in males (OR = 0.32) than in females (OR = 0.64; Supplementary Table S13). This sex dimorphism was model-dependent (null in the per-animal ANOVA, *p* = 0.156; borderline in the daily-level mixed model, *p* = 0.067; significant only in the binomial count-level GLM; Section 3.2.4) and should be regarded as a candidate rather than confirmed interaction, pending replication. One interpretation is that the circadian-arousal component of the trough weakens more in aged males than in aged females; altered homeostatic scaling or threshold instability could generate a similar phenotype. The age-related attenuation of the analogous human evening arousal signal [25] is consistent with a circadian component. Transition-zone nodes such as the trough depend on tight coupling among competing regulatory drives and may be especially vulnerable to age-related loosening of coupling, plausibly explaining why age × sex effects concentrate at the trough while 24 h totals remain relatively preserved.

One speculative possibility, not supported by our data but motivating future work, is that the fine temporal structure of the N-shape is additionally modulated by a ~12 h transcriptional program operating alongside the canonical circadian clock. A cell-autonomous ~12 h program centered on the XBP1s–IRE1α branch of the unfolded protein response coordinates rhythmic proteostatic and lipid-remodeling capacity in peripheral rodent tissues, with outputs peaking near the two diurnal transitions [40,41,42,43]. The N-shape landmarks (ZT18–ZT3) fall within one of these transitions. Whether this phase alignment reflects a causal link or simply parallel tracking of major physiological transitions in nocturnal mammals cannot be distinguished without direct measurement of a 12 h program in rodent sleep-regulatory brain nuclei. At present, this remains a systems-level hypothesis rather than a mechanistic claim supported by the dataset.

#### 4.3.2. The Dark-Phase Reduction in Theta-Dominant Wake

The most robust finding across analytical levels was a marked dark-phase reduction in TDW in aged mice of both sexes (−17 to −18%). This was the largest age contrast in the dataset and along with dark-phase NREM and light-phase REM in females, one of four phase-level effects exceeding the minimum detectable effect size at 80% power for the present design. The result was supported convergently by phase-level contrasts, omnibus LMMs, GAM profiles, bout statistics, survival models, and bi-exponential mixture decomposition, arguing against a statistical artifact. Importantly, TDW loss was redistributed across nTDW and NREM rather than offset by any single state, indicating a shift from consolidated, locomotor-engaged wake to drowsier and more fragmented vigilance states. CLR analysis further confirmed that this was not a compositional artifact.

Episode-level analyses showed that aging altered not only the amount but also the structure of TDW: dark-phase TDW bouts were shorter, more fragmented, and had reduced time constants for both short- and long-bout components, consistent with a generalized loss of wake consolidation. Survival modeling supported this interpretation, showing faster TDW termination in old mice and a complementary pattern in nTDW, with longer dark-phase nTDW bouts but shorter light-phase bouts. Combined with the elevated NREM → wake transition rate, these findings suggest that aging preserves the ability to interrupt sleep but impairs the stabilization of active wake. This pattern is consistent with age-related weakening of arousal-promoting systems, including orexin/hypocretin and LC–norepinephrine pathways, although this mechanistic interpretation remains provisional and will require direct circuit-level testing [44,45,46].

#### 4.3.3. Fragmentation Is State- and Phase-Specific, Not a Single Scalar Phenotype

A key contribution of this study is the demonstration that sleep–wake fragmentation in aged mice is not uniform, but state-specific, circadian-phase-dependent, and directionally heterogeneous. Conventional fragmentation indices collapse distinct processes into a single measure, obscuring whether aging shortens wake or sleep bouts, increases brief awakenings, or alters transition probabilities. By separating these components, we find that aging affects wake sub-states in opposite directions, shortened TDW but prolonged dark-phase nTDW, alters sleep states unequally, increased NREM episode number but preserved REM episode duration, and concentrates many of these effects within specific circadian windows. Aging also eliminates sex-specific baseline differences: in young animals, light-phase NREM → wake transition rates are substantially lower in females than in males (female/male RR = 0.67, *p* = 0.008), but this sex difference disappears in aged animals (RR = 0.98, *p* = 0.89; Supplementary Table S18), indicating loss of a sex-dependent component of sleep stability.

For REM sleep, aging primarily reduced episode occurrence, while largely preserving duration, suggesting that aging primarily impairs REM recruitment rather than REM maintenance. Consistent with this interpretation, aged mice show reduced NREM → REM transition rates, and reduced hourly probability of REM occurrence in aged males. Together, these findings suggest impaired recruitment of REM-generating mechanisms rather than destabilization of REM once initiated [47].

#### 4.3.4. NREM Termination and REM Reduction

Quantifying NREM → wake and NREM → REM transitions provided a complementary index of sleep stability. Aging shifted NREM termination toward wake and reduced transitions into REM, consistent with increased fragmentation and impaired REM recruitment. This pattern aligns with models in which wake, NREM, and REM emerge from competitive interactions among reciprocal regulatory networks [46,47]. Although an age × sex × phase interaction was detected for NREM → wake transitions, this result should be interpreted cautiously at the current sample size.

Notably, the strongest transition abnormalities occurred in the late-dark/early-light interval, the same circadian window in which TDW loss, ultradian block fragmentation, and out-block frequency diverged most strongly with age. This convergence suggests that specific circadian windows are especially sensitive to aging-related instability and may represent the most sensitive targets for interventions aimed at stabilizing sleep–wake architecture.

#### 4.3.5. Ultradian Block Organization Captures Higher-Order Disruption of NREM–REM Cycling

Ultradian block analysis extends sleep characterization from individual bouts to the integrity of NREM–REM cycling as a coherent structural unit. A block, defined by the 3 min wake-gap bridging threshold (robust to 2- and 4 min sensitivity analyses), captures the sleep component of the ultradian oscillation; including inter-block wake, one complete cycle extends to ~2–4 h, similar to locomotor and metabolic ultradian rhythms in nocturnal rodents [31]. Block length is strongly circadian-modulated, mirroring the N-shape motif. Aging shifts this organization in a circadian-phase-dependent fashion: in the adjusted fragmentation model (Supplementary Table S23), the age × period interaction was significant in both light-phase segments (age × early-light RR = 0.73, *p* = 0.025; age × late-light RR = 0.61, *p* < 0.001), consistent with longer, less-interrupted light-phase blocks in aged mice; dark-phase blocks showed a directionally consistent but non-significant shortening (RR = 1.29, *p* = 0.138). Aging also increases the frequency of short out-block episodes, with the hurdle model confirming that out-blocks are statistically separable from normal blocks in both presence (age OR = 2.72, *p* = 0.010) and conditional count (age RR = 1.37, *p* < 0.001) components (Supplementary Table S24). Threshold-robustness analysis (Supplementary Table S30) confirmed this age effect across most alternative threshold settings.

Two candidate neural substrates with appropriate timescales merit consideration as contributors to the ultradian organization observed here. The dopaminergic ultradian oscillator (DUO) generates ~4 h rhythms of behavioral arousal that persist in SCN-lesioned and Bmal1^−^/^−^ mice [48,49], plausibly modulating inter-block wake. Separately, ultradian calcium dynamics in the glutamatergic SPZ–PVN network [33,34] span 0.5–4 h and could provide a sub-24 h hypothalamic timing scaffold. Neither substrate is established by our data; experimental dissociation will be required to determine their relative contributions.

Within this framework, the ~24 h modulation of block length most parsimoniously reflects phase-dependent gating of ultradian expression rather than modulation of an ultradian generator. Process S and Process C may determine when ultradian transitions become behaviorally visible as observable block–wake alternation rather than creating the ultradian cycle itself. Block-length modulation would then be a classic Aschoff-style masking phenomenon: the underlying ultradian organization is present across the cycle, but its behavioral expression is phase-dependent.

The statistical separability of out-blocks supports the hypothesis that they represent a distinct expression mode of incomplete block assembly rather than the short tail of a continuous distribution. Out-blocks may reflect episodes with sufficient drive for transient NREM entry but insufficient for progression into full NREM–REM block organization—a failure at block assembly rather than premature termination. The assembly-failure interpretation remains one hypothesis among several consistent with the observed separability.

The age-related pattern—longer, less-fragmented light-phase blocks alongside a directional tendency toward shorter dark-phase blocks and increased out-blocks—cannot be explained by generic fragmentation, which would predict unidirectional shortening. It instead suggests bidirectional loss of gating fidelity between the ultradian organization and the slower regulatory layers shaping its behavioral expression: dark-phase weakening of wake-promoting opposition allowing weak ultradian sleep-promoting events to penetrate behavior, and light-phase strengthening of sleep-promoting dominance resisting interruption. Out-blocks would represent a third expression of the same loss of gating fidelity. Consistent with a transition-zone coupling-vulnerability framework, the strongest effects concentrate at the trough of the N-shape and across the dark-to-light transition. Direct validation requires experimentally dissociating the ultradian organization from its gating layers.

#### 4.3.6. Translational Interpretation of Ultradian Block Organization

We hypothesize that a consolidated rodent ultradian block may represent a temporally compressed analog of a human night’s sleep, with rodents expressing multi-cycle NREM–REM architecture on a shorter timescale consistent with broader allometric scaling of cardiac, respiratory, metabolic, and neural dynamics. Rodent block architecture is also strongly shaped by circadian phase—shorter and less consolidated during the dark active phase, longer and more consolidated during the light resting phase—paralleling the reduced continuity of human sleep at adverse circadian phases [24,25,50]. Although formal homology remains unproven, these organizational similarities support the use of ultradian block metrics as informative translational endpoints in preclinical studies of sleep consolidation.

#### 4.3.7. Age- and Sex-Dependent Changes in EEG Spectral Architecture

State- and phase-resolved spectral analysis showed that aging shifted TDW toward lower frequencies, consistent with the prior rodent and human EEG-aging literature [5,51,52]. The theta-slowing is supported by three convergent absolute-PSD analyses, cluster-based permutation testing (CBPT age clusters in TDW at 2.8–7.6 Hz dark and 2.1–7.1 Hz light; Section 3.6.1), scalar TDW centroid-frequency analysis (β = −0.135 dark, β = −0.134 light; both q_FDR < 0.001; Supplementary Table S28), and FOOOF spectral parameterization (TDW theta center frequency β = −0.781; q_FDR = 0.003; Supplementary Table S29), indicating genuine neurophysiological slowing rather than normalization-dependent redistribution. Complementary relative-PSD analyses (§3.6.3) showed widespread age-related proportional redistribution in waking states (168/191 and 175/191 bins in TDW-dark and nTDW-dark respectively), which should be interpreted alongside, rather than instead of, the absolute-power effects [53,54]. State specificity, an age-related decline in NREM delta power (β = −0.159 dark, β = −0.122 light; Supplementary Table S28) occurring alongside wake-theta slowing, argues against a nonspecific amplitude artifact, which would produce same-direction effects across states.

The REM theta age × sex interaction is supported by both CBPT (theta-band interaction cluster at 3.5–7.9 Hz dark, with an additional alpha-band cluster at 9.6–15.5 Hz dark; pan-spectrum cluster at 2.8–20.0 Hz light; Section 3.6.1) and FOOOF (Theta PW REM age × sex, β = +0.125 indicating attenuation of the age-related decline in females; q_FDR = 0.042; Supplementary Table S29), though the scalar REM-theta analysis did not reach FDR significance for this interaction. Together, the REM and waking-state patterns suggest sex-dependent spectral aging that warrants confirmation in larger cohorts. More broadly, these results suggest that EEG-based aging markers may require sex stratification, because spectral signatures present in one sex may be absent, weaker, or reversed in the other [1,55,56,57].

#### 4.3.8. Sampling Optimization: Circadian Bottlenecks, REM Difficulty, and Diminishing Returns

Variance structure differed across age and sex strata in ways that have practical implications for design. Old mice exhibited higher between-animal reliability than young mice (mean ICC: OM 0.45, OF 0.39 vs. YM 0.28, YF 0.22), driven primarily by lower day-to-day variance; young females were the least statistically efficient subgroup, with the highest within-animal variance (up to 4.5-fold across metrics). Stabilization demands were highest for dark-phase and REM endpoints. Power gains rose rapidly over the first 4–6 recording days and then plateaued. Extended recording is most justified when the aim is to quantify infradian variability, analyze high-variance REM measures, or preserve fine circadian segmentation.

A supplementary variance decomposition analysis indicated that, for most architectural endpoints in this dataset, gains in inferential precision were driven more strongly by sample size than by additional recording days (Supplementary Note S1). This analysis is provided as practical design guidance rather than as a primary finding of this study.

#### 4.3.9. Sex Differences: Hypothesis-Generating Observations Within a Power-Limited Design

A transparent account of statistical power is essential for interpreting the sex-related findings. At *n* = 6 per group, only one sex contrast survived correction across the 32 between-group phase-level comparisons: young females showed 5.9 percentage points less light-phase NREM than young males (g = −2.60; *p*Holm = 0.005). This difference was not evident in old mice, raising the possibility that the sex effect seen in young animals may depend on gonadal hormonal state and diminish with reproductive aging. All other sex contrasts were more modest and did not survive multiple-comparison correction.

At the same time, omnibus sex × hour interactions were significant in three of the four vigilance states (TDW, NREM, and REM) and marginal in nTDW, and several sex-dependent patterns recurred across analytical levels, including higher NREM peak amplitudes in males, higher NREM → REM transition rates in females, and a distinct spectral profile in young females. Because estrous staging was not performed (see Limitations), the transition- and spectral-based sex patterns in particular cannot be fully separated from cycle-dependent hormonal modulation. These patterns are broadly consistent with prior reports of sex differences in mouse sleep [14,55,56,57], but at the current sample size they should be regarded as hypothesis-generating rather than definitive evidence for sex-specific aging trajectories. Larger cohorts, ideally with estrous tracking in young females and hormonal phenotyping in old females, will be needed to establish their robustness and mechanism.

#### 4.3.10. Limitations

Several limitations constrain inference in the present study. First, single-channel EEG limits spatial resolution and cannot address regional heterogeneity in sleep oscillations, including local sleep, topographic sigma patterns, or region-specific age effects on slow waves [1,6,58]. Multi-site EEG or local field potential recordings will be needed to determine whether the spectral changes observed here are globally expressed or region-specific.

Second, spectral analyses used a 72 h subset rather than the full 14-day recording. Although full-duration vigilance-state analyses showed no acclimation effect or secular drift, and the subset preserved the dark-phase TDW age effect, these controls do not establish full temporal stability of the spectral phenotype itself. Spectral findings should therefore be regarded as secondary and confirmed by full-duration spectral estimation or independent replication. Biophysical factors such as age-related skull thickening, electrode impedance, or other recording-interface differences also cannot be excluded as contributors to broadband absolute-power differences.

Third, estrous cycle staging was not performed. Although 14-day recording likely averaged across multiple cycles in young females, the absence of cycle-phase information means that some sex differences may partly reflect cyclic hormonal modulation rather than sex alone. This consideration applies most directly to state-transition dynamics and spectral features, which are more likely than coarse state-occupancy metrics to be sensitive to cycle-dependent hormonal variation; sex-related findings should therefore be interpreted with corresponding caution. Estrous tracking should therefore be incorporated in future work.

Fourth, the sample size of *n* = 6 per group provides adequate power mainly for large effects, leaving moderate effects and age × sex interactions underpowered. Accordingly, sex-related findings and interaction effects should be interpreted as provisional, as some significant estimates may be inflated by low-power bias.

Fifth, the use of only two age points (4 and 24 months) limits inference about developmental trajectories. Intermediate ages will be required to determine when these phenotypes emerge and whether they progress linearly or nonlinearly.

Sixth, vigilance-state scoring relied on single-channel EEG, and the acquired EMG signal was not used analytically. Although the classifier used here has been validated for single-channel EEG-based scoring [18], EEG-only classification may be less sensitive than combined EEG/EMG scoring to low-movement wakefulness, REM atonia-dependent features, and ambiguous transitional states.

Finally, the findings are specific to C57BL/6J mice under standard laboratory conditions. Replication across sub-strains and more genetically diverse populations will be needed to assess the generality of these phenotypes [9,56].

### 4.4. Hypotheses for Future Work and Translational Relevance

#### Translational Relevance

Comparisons to human sleep in aging are most defensible at the level of organizational principles rather than specific quantitative parameters. In both species, aging is associated with greater sleep–wake instability, increased bias of NREM termination toward wakefulness, alterations in slow-wave-related spectral features, and concentration of vulnerability within specific circadian windows. The dark-phase reduction in theta-dominant wakefulness in aged mice parallels the reduced ability of older humans to sustain consolidated, alert wakefulness during the biological day, reflected in greater daytime sleepiness and increased napping propensity [1,51]. The shift from theta-dominant to non-theta-dominant wakefulness may also be relevant, as human studies have linked greater time in drowsy or disengaged wakefulness to neurodegenerative risk and prodromal cognitive decline [59,60], although direct equivalence between rodent TDW/nTDW and these human wake phenotypes remains unestablished.

The out-block phenomenon may also have translational relevance, though this remains speculative. In aged mice, out-blocks appear to reflect incomplete assembly of full ultradian sleep cycles; whether an analogous process contributes to interrupted sleep consolidation in older humans is unknown. If validated, out-block frequency could become a useful preclinical endpoint for interventions targeting sleep consolidation in aging. More broadly, defining these organizational principles in mice provides a scalable platform for mechanistic and interventional studies, including manipulation of orexinergic, cholinergic, and GABAergic systems, environmental enrichment, and chronotherapeutic targeting of circadian vulnerability windows, without implying direct equivalence of sleep-cycle duration or circadian phase structure across species.

## 5. Conclusions

The 14-day, multi-domain analysis approach used here detects age- and sex-related features of sleep–wake organization that are often missed or cannot be reliably detected by conventional short-duration, coarse-bin, male-biased designs, and can be extended to other perturbations such as stress, pharmacological challenge, or disease models. By resolving circadian phase-specific sleep–wake dynamics, state-specific fragmentation, ultradian block organization, transition kinetics, and spectral architecture while accounting for intra-individual variability across 14 days of continuous recording, this approach improves detection of subtle but biologically meaningful phenotypes in mouse sleep–aging research.

The principal findings, the dark-phase reduction in TDW, the TDW-to-nTDW shift at the pre-lights-on N-shape trough, altered bout-length structure, imbalance of NREM → wake and NREM → REM transitions, and the emergence of out-block episodes, show that aging is not simply an increase in sleep fragmentation, but a multi-dimensional reorganization of sleep–wake architecture across epochal, ultradian, and circadian scales. These results align with emerging human studies showing that age-related sleep disruption is phase-specific, state-dependent, and structurally multi-dimensional rather than a single scalar deficit. These findings are based on C57BL/6J mice at two discrete ages (4 and 24 months) in a cross-sectional design; developmental trajectories cannot be inferred from two time points, and sex-related and interaction findings should be regarded as hypothesis-generating pending replication in larger cohorts.

## Data Availability

The original contributions presented in this study are included in the article/Supplementary Materials; further inquiries can be directed to the corresponding authors.

## Supplementary Materials

The following supporting information can be downloaded at: https://www.mdpi.com/article/10.3390/cells15121075/s1, Figure S1: TDW classification robustness to θr threshold. Figure S2: Residuals of the 24 h vigilance-state LMMs are approximately Gaussian with modest per-mouse autocorrelation in TDW. Figure S3: LMM fits for 24 h vigilance-state profiles converge stably across optimizers and show well-conditioned variance components. Figure S4: Two-harmonic cosinor fits (24 h + 12 h) capture the bimodal TDW profile and the unimodal NREM profile with R^2^ = 0.57–0.85 across groups. Figure S5: Age effects with large effect sizes cluster in dark-phase wake and in female-specific NREM and REM contrasts. Figure S6: Cascade-gated hourly EMM contrasts localize where within each phase the age effect peaks; 17 of 120 cells reach Holm-adjusted significance. Figure S7: Sex contrasts are small and inconsistent across vigilance states; only light-phase NREM in young animals shows a Holm-surviving difference. Figure S8: Sex effect sizes cluster near |g| = 0.9–1.4 in dark-phase wake states but have confidence intervals that cross zero after correction. Figure S9: Age-related effect sizes concentrate in the early-to-mid dark phase for TDW and mirror-image for NREM, with the largest effects at ZT12–ZT18. Figure S10: Full set of 32 phase-level between-group contrasts with bootstrap 95% CIs; four contrasts survive Holm–Bonferroni correction within state. Figure S11: Aging reduces TDW fraction at the N-shape onset (ZT16, Tier 1 confirmatory) and at the pre-lights-on trough (Tier 2 descriptive); other N-shape landmarks are not Holm-surviving. Figure S12: Global ultradian NREM metrics are largely preserved across age × sex groups; only weighted circular variance of peak timings shows a nominal age effect. Figure S13: Aging deepens early-dark NREM troughs; peak amplitude is higher in males; oscillation range is preserved across groups. Figure S14: State composition at N-shape transitions: peaks are NREM-dominated, the trough is wake-dominated, and trough wake composition shifts from TDW toward nTDW with aging. Figure S15: TDW fraction at the five pre-specified fixed ZT reference points. Figure S16: Primary N-shape landmarks are stable across preprocessing variants; the trough TDW-fraction age effect is robust to leave-one-out, day-slope, and bimodality sensitivity analyses. Figure S17: Phase-level bout duration and episode count confirm the dark-phase TDW shortening, nTDW lengthening, and light-phase NREM episode increase patterns of the hourly analysis (Figure 4). Figure S18: TDW episode-count distributions are bi-exponential, with aging collapsing both the short-bout and long-bout components during the dark phase. Figure S19: NREM and REM episode-count distributions show age-related increase in NREM episode number and largely preserved REM episode architecture. Figure S20: nTDW episode-count distributions lengthen with age during the dark phase, consistent with a shift from consolidated TDW toward drowsier nTDW wake. Figure S21: Phase-resolved NREM exit rates show an age-related increase in NREM → wake and a decrease in NREM → REM, with sex differences in light-phase NREM → wake rates converging in old animals. Figure S22: REM gating probability is reduced in aged males during the dark phase but preserved in aged females. Figure S23: Aging raises intra-block wake fraction at the expense of NREM; young animals retain higher intra-block REM. Figure S24: Isolated short sleep fragments (out-blocks) accumulate with aging, cluster at <500 s, and are most frequent in old females. Figure S25: Out-block episodes accumulate disproportionately in aged females across most of the 24 h cycle, with peak density in the dark phase. Figure S26: Intra-block NREM → wake transition rate is elevated in aged mice, with the largest age gap in dark-phase old males. Figure S27: Intra-block NREM → REM transition rate is reduced in aged mice, most prominently during the dark phase. Figure S28: Valid ultradian blocks are shorter and less consolidated in aged mice during the dark phase; the distribution shifts toward shorter blocks with aging. Figure S29: Frequency-resolved t-statistic maps confirm that the CBPT-defined clusters coincide with the local maxima of effect-size density. Figure S30: Age effects on absolute PSD reach |Cohen’s d| > 0.8 in wake-state theta bands and in NREM sigma, with the crossed group contrast (OF vs. YM) showing the largest phenotypic separation. Figure S31: Relative PSD reveals that age effects remain frequency-selective in wake states (FDR-significant in ~90% of bins in TDW-dark and nTDW-dark) but are more restricted in NREM, where much of the broadband age effect in absolute PSD reflects global scaling. Figure S32: Hourly time-course of normalized EEG spectral power during TDW shows the largest age-related redistribution at dark-phase onset (ZT12) in the sigma/high-alpha range (10–13 Hz). Figure S33: Hourly time-course of normalized EEG spectral power during nTDW; early-dark 1–2 Hz attenuation is the dominant age effect. Figure S34: Hourly time-course of normalized EEG spectral power during NREM; age-related changes concentrate in late-dark delta (3–4 Hz) and early-light low frequencies (1–2 Hz). Figure S35: Hourly time-course of normalized EEG spectral power during REM shows broad but low-amplitude age- and sex-related effects, with higher REM data sparsity than other states. Figure S36: Partial-η^2^ magnitudes confirm that the age effect is concentrated in early-dark TDW sigma/high-alpha (η^2^p up to 0.70) and in nTDW 1–2 Hz at ZT13 (η^2^p up to 0.65). Figure S37: Convergence map across normalizations identifies four core age effect loci in TDW dark phase (ZT12, 10–13 Hz) and one in nTDW dark phase (ZT13, 1–2 Hz) that survive all three reference frames. Figure S38: Cross-normalization comparison of the top-20 age effect cells; TDW 10–13 Hz at ZT12 and nTDW 1–2 Hz at ZT13 appear in all three rankings. Figure S39: Top 20 age effect cells concentrate in the dark phase of wake states across all three normalizations, with REM contributing more cells under ZT8–11 anchoring. Figure S40: Frequency distribution of top 20 age effect cells across the 19 spectral bands shows that sigma/high-alpha (10–13 Hz) and low-frequency (1–3 Hz) loci dominate, regardless of normalization choice. Figure S41: Convergent age effect loci—TDW 10–13 Hz at ZT12 and nTDW 1–2 Hz at ZT13—are detectable across all three normalization frameworks. Figure S42: FOOOF spectral parameterization shows age-related reductions in theta center frequency across TDW, nTDW, and NREM, with robust age × sex interactions in aperiodic offset during wake. Figure S43: Representative example of the empirical evaluation used to select the wake-bridging threshold. Figure S44: Bimodality of single-state block durations supports the 500 s out-block threshold. Figure S45: Schematic illustration of the three normalization reference frames used in the hourly spectral-distribution analysis. Table S1: Robustness of Tier 2 LMM hour-containing interaction tests to time-factor parameterization. Table S2: Variance components and convergence diagnostics from the random-intercept LMM on 24 h vigilance-state profiles. Table S3: Nakagawa’s marginal and conditional R^2^ for the random-intercept LMM on 24 h vigilance-state profiles. Table S4: Per-mouse autocorrelation diagnostics of LMM residuals on 24 h vigilance-state profiles. Table S5: Phase-level between-group and within-group paired contrasts on vigilance-state percentages, with Holm–Bonferroni correction. Table S6: Two-harmonic cosinor parameters fitted to 24 h vigilance-state profiles per group. Table S7: Sensitivity analysis: significant phase-level age contrasts after excluding five mice with within-mouse CV > 0.80. Table S8: Bootstrap difference-smooth clusters from Phase-1 GAM analysis of 24 h NREM profiles. Table S9: Global ultradian metrics (24 h). Table S10: TDW fraction at five pre-specified fixed ZT reference points: per-group means (Panel A), age main effect (Panel B), and sex/interaction effects (Panel C). Table S11: Tier 2 descriptive test—factorial age × sex ANOVA on TDW fraction at the data-driven trough (Section 3.2.3). Table S12: Exploratory N-shape landmarks (Tier 3). † Trough ZT values are reported as circular mean ± circular SD (in ZT clock hours on the 24 h cycle); hypothesis tests for trough ZT were performed on the linear representation “hours after ZT20” (values in [0, 6] h, where 0 corresponds to ZT20 and 6 corresponds to ZT2). Table S13: Binomial GLM on wake microcomposition at the N-shape trough. Table S14: ICC values for primary landmarks across preprocessing variants. Table S15: Day-level validation: mixed-effects models on daily TDW fraction. Table S16: Bi-exponential mixture decomposition of TDW bout-length distributions (Section 3.3.5). Table S17: NREM → wake NB GLMM fixed-effect estimates. Table S18: NREM → wake planned contrasts (EMMs, rate ratios). Table S19: NREM → REM NB GLMM fixed-effect estimates. Table S20: Model comparison for NREM-exit transition models (Section 2.9). Table S21: Per-mouse cosinor parameters for ultradian block architecture (Section 3.5). Table S22: Age × sex ANOVA on per-mouse cosinor parameters of ultradian block architecture; significant effects only. Table S23: Poisson GEE rate ratios for ultradian-block fragmentation, adjusted for total sleep duration (Section 2.10, Analysis 2). Table S24: Out-block hurdle model (Analysis 4). Table S25: Summary of significant CBPT clusters on absolute PSD. * *p* < 0.05, ** *p* < 0.01. Table S26: Cells appearing in the top 20 by η^2^p (age effect) in two or more normalization frameworks. Table S27: The 20 h × band × state cells with the largest partial η^2^p for the age effect. Table S28: Scalar band-power linear-model results (Section 3.6.3); only FDR-significant effects shown (q_FDR < 0.05). Table S29: FOOOF spectral-parameterization linear-model results (Section 3.6.3); only FDR-significant effects shown (q_FDR < 0.05). Table S30: Sensitivity of the age effect on out-block count to block-identification threshold choice. Table S31: Variance structure and stability summary for Tier 1 sleep metrics. Table S32: Design recommendations for translational sleep–aging studies in C57BL/6J mice. Supplementary Methods S1: CLR-transformed compositional analysis. Supplementary Methods S2: Threshold rationale and sensitivity analyses for ultradian-block identification. Supplementary Methods S3: Statistical analysis of 24 h vigilance-state profiles, full specifications [61]. Supplementary Methods S4: Statistical analysis of NREM temporal architecture, full phase specifications. Supplementary Methods S5: Statistical analysis of episode architecture and bout-length distributions, full specifications. Supplementary Methods S6: Statistical analysis of EEG spectral data, full tiered architecture. Supplementary Note S1: Dataset validation, variance structure, and design implications [62,63].

## Abbreviations

The following abbreviations are used in this manuscript:

ACF	autocorrelation function
AIC	Akaike information criterion
ANOVA	analysis of variance
AP	absolute precision
BLUP	best linear unbiased predictor
BW	bandwidth
CBPT	cluster-based permutation testing
CF	center frequency
CI	confidence interval
CLR	centered log-ratio
CV	coefficient of variation
EEG	electroencephalogram
EMG	electromyogram
EMM	estimated marginal mean
FDR	false discovery rate
FOOOF	fitting oscillations and one over f
FWER	family-wise error rate
GAM	generalized additive model
GEE	generalized estimating equations
GLM	generalized linear model
GLMM	generalized linear mixed-effects model
IACUC	Institutional Animal Care and Use Committee
ICC	intraclass correlation coefficient
LMM	linear mixed-effects model
LRT	likelihood-ratio test
ML	maximum likelihood
NB	negative binomial
NREM	non-rapid eye movement (sleep)
nTDW	non-theta-dominated wakefulness
OF	old female
OM	old male
OR	odds ratio
PSD	power spectral density
PVN	paraventricular nucleus
PW	peak power
REM	rapid eye movement (sleep)
RR	rate ratio
SD	standard deviation
SEM	standard error of the mean
SPZ	sub-paraventricular zone
TDW	theta-dominated wakefulness
VA	Veterans Affairs
WMZ	wake-maintenance zone
YF	young female
YM	young male
ZINB	zero-inflated negative binomial
ZT	Zeitgeber Time
