# Supplementary material for: A Hierarchical and Multiscale Framework for Characterizing Mouse Sleep–Wake Dynamics from 14-Day Continuous EEG: Validation of Age- and Sex-Dependent Remodeling
Source: Cells. 2026 Jun 13;15(12):1075. doi: 10.3390/cells15121075 (PMC13296934; doi:10.3390/cells15121075)
Supplement: Supplementary file 1 [file cells-15-01075-s001.zip › Supplementary Figures.pdf]

# SUPPLEMENTARY FIGURES

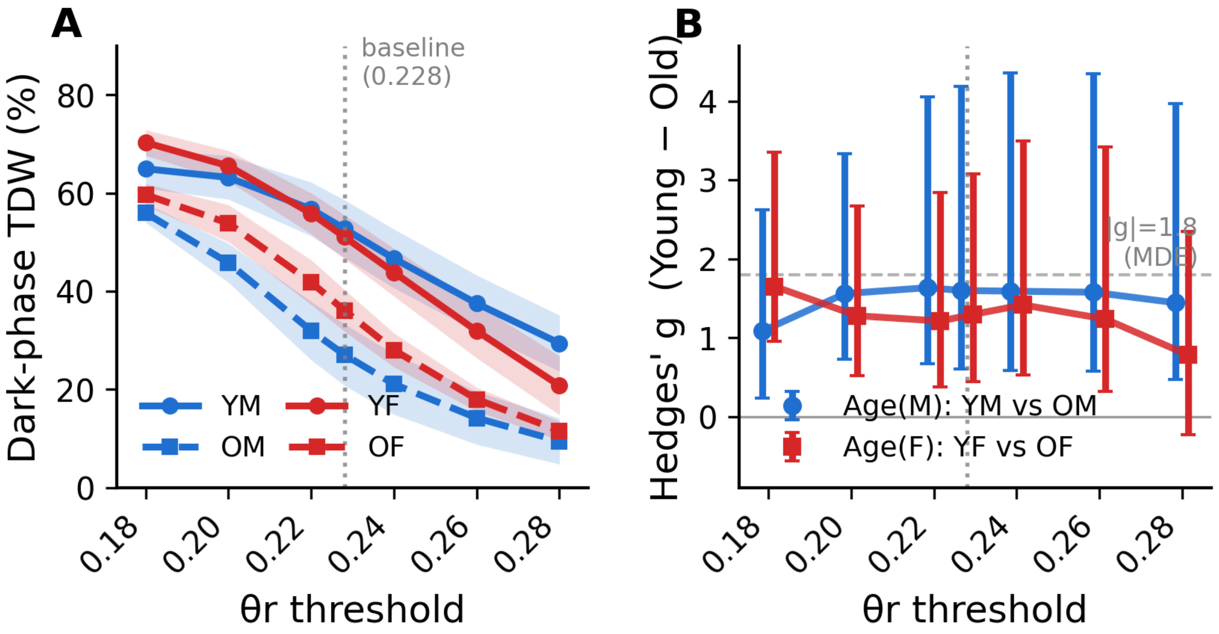


**Supplementary Figure S1. TDW classification robustness to θr threshold.** Sensitivity analysis of the TDW/nTDW classification boundary across θr ∈ {0.18, 0.20, 0.22, 0.228, 0.24, 0.26, 0.28} on the 3-day spectral subset (n = 6 per group). **(A)** Group-level dark-phase TDW% as a function of θr threshold. Young males (YM, solid blue), young females (YF, solid red), old males (OM, dashed blue), old females (OF, dashed red). Shaded bands indicate ±1 SEM. Dotted vertical line marks the manuscript's baseline threshold (θr = 0.228). All four curves decline monotonically with threshold and remain approximately parallel, preserving the group rank order (YF ≈ YM > OF > OM) across the entire sweep. **(B)** Sex-stratified Hedges' g for the Young − Old contrast as a function of θr, with 95% bootstrap CIs. Dashed horizontal line at |g| = 1.80 indicates the minimum detectable effect size at 80% power for n = 6 per cell. The Age effect direction (Young > Old) is preserved at every threshold and in both sexes; effect size remains stable across the sweep with a mild peak near θr ≈ 0.24.

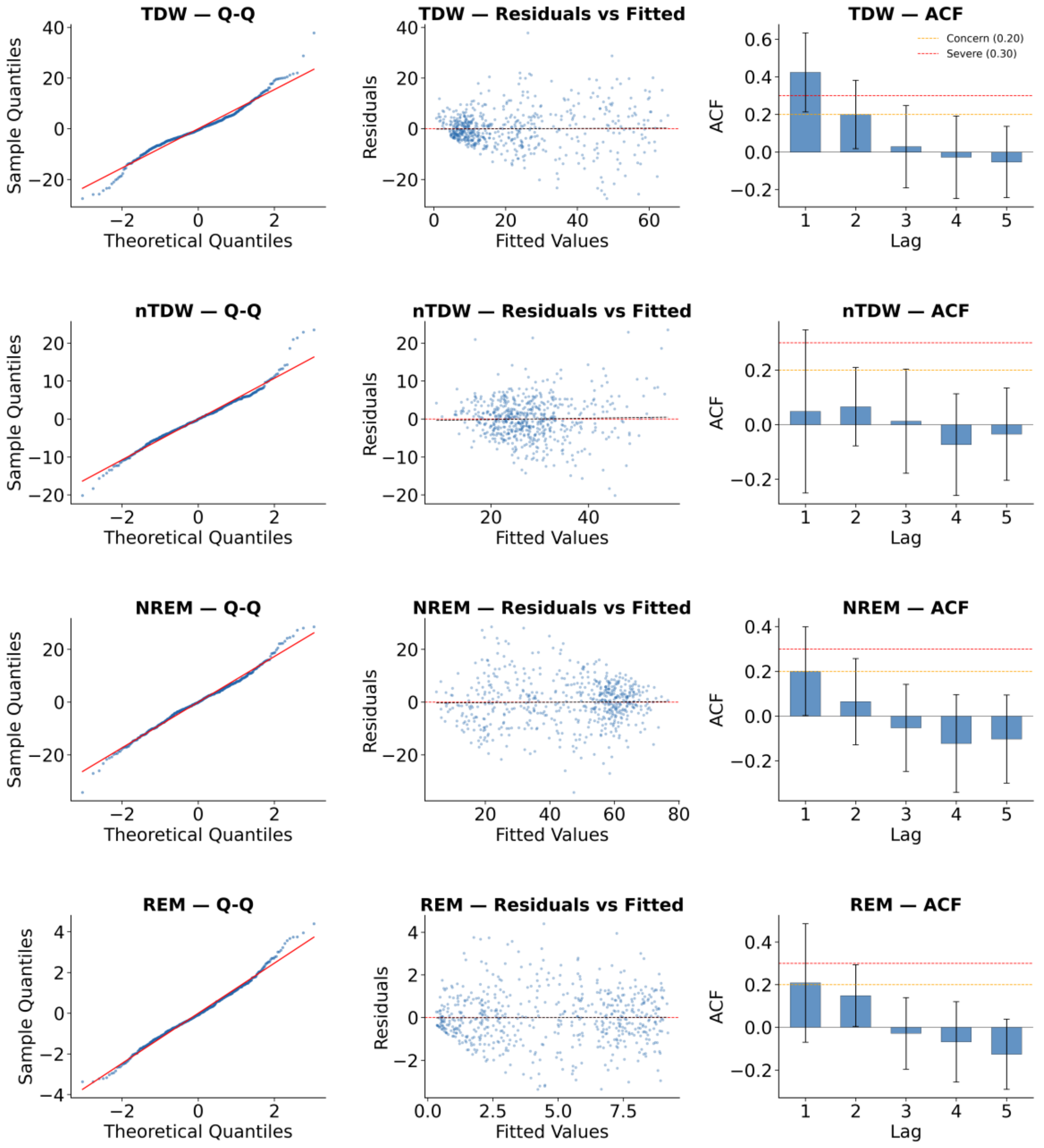


**Supplementary Figure S2. Residuals of the 24-hour vigilance-state LMMs are approximately Gaussian with modest per-mouse autocorrelation in TDW.** Per-state diagnostics of the Gaussian LMMs described in Section 2.6 (model: state% ~ Age × Sex × Hour + (1 | Mouse_ID)). (A) Q–Q plots of Pearson residuals against theoretical Gaussian quantiles for TDW, nTDW, NREM, and REM; dashed reference line = identity. (B) Residuals vs. fitted values, overlaid with a LOESS smoother (span = 0.5) to check mean–variance drift. (C) Per-mouse autocorrelation functions (lag 1–12 hours) displayed as spaghetti plots with the group median in bold; horizontal dashed line = 95% white-noise envelope. Summary statistics of ACF(1) and Bartlett's effective N are reported in Supplementary Table S3. n = 6/group.

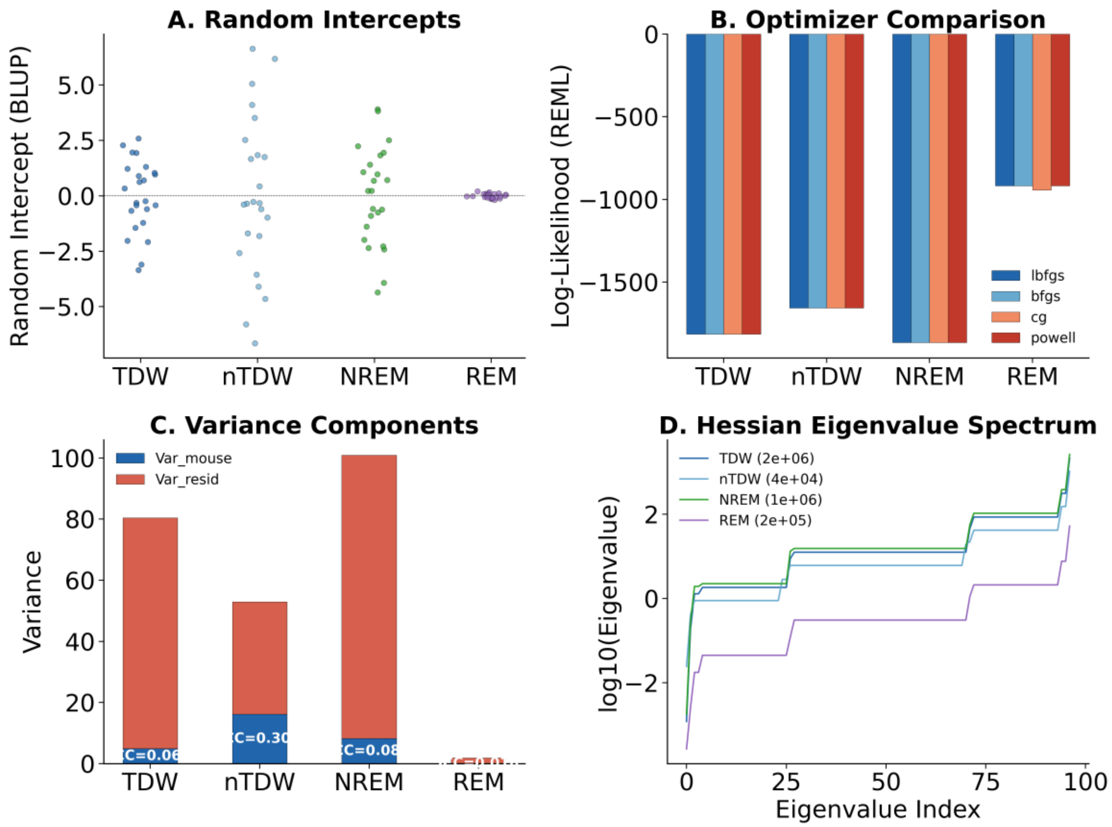


**Supplementary Figure S3. LMM fits for 24-hour vigilance-state profiles converge stably across optimizers and show well-conditioned variance components.** Numerical convergence diagnostics for the random-intercept LMM (Section 2.6). (A) Best linear unbiased predictors (BLUPs) of the Mouse_ID random intercept plotted against mouse ID; histogram overlay tests approximate normality. (B) Log-likelihood values from four optimizers (L-BFGS, BFGS, conjugate gradient, Powell) at the REML solution, displayed separately per state to confirm convergence to equivalent likelihood values. (C) Variance components ($\sigma^2$_between, $\sigma^2$_within) with 95% profile-likelihood CIs per state. (D) Eigenvalue spectrum of the Hessian; ratios of max/min eigenvalue > $10^6$ would flag ill-conditioning. All panels: TDW, nTDW, NREM, REM separately. n = 6/group.

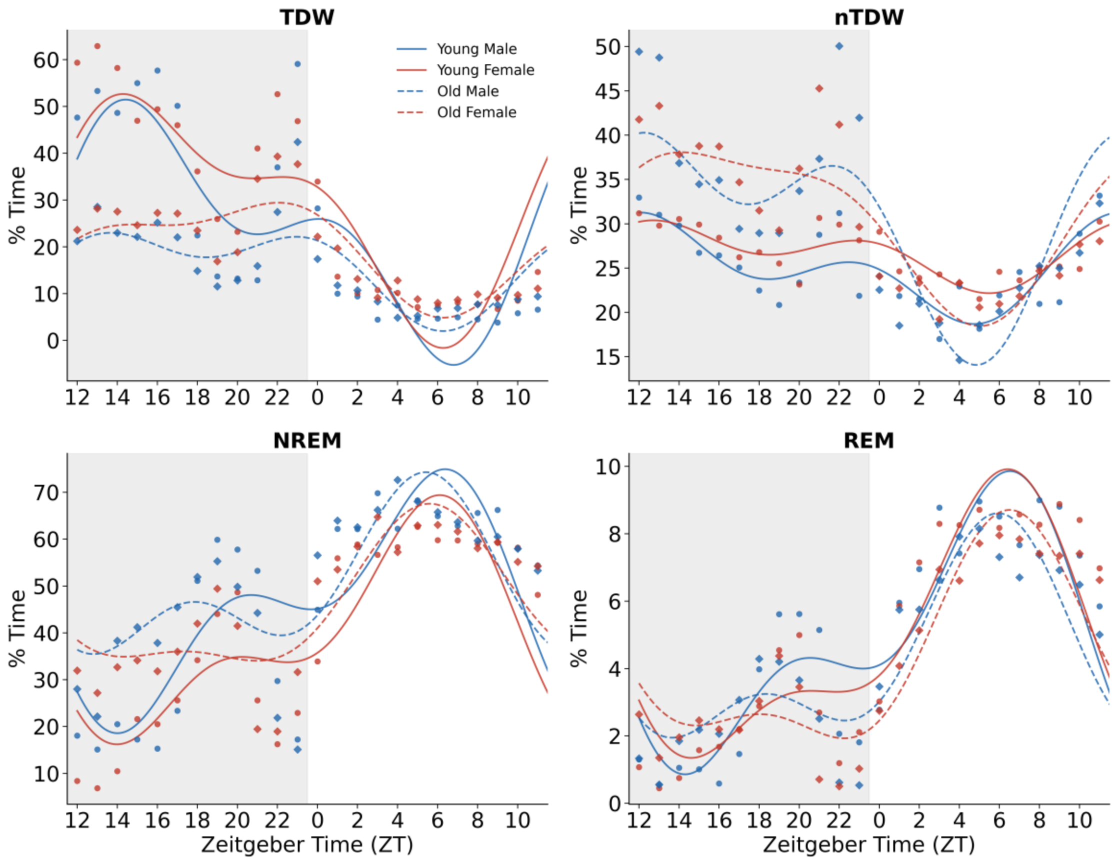


**Supplementary Figure S4. Two-harmonic cosinor fits (24-h + 12-h) capture the bimodal TDW profile and the unimodal NREM profile with $R^2$ = 0.57–0.85 across groups.** Group-averaged 24-hour vigilance-state profiles (15-min bins) overlaid with the fitted two-harmonic cosinor model, $Y(t) = M + A_1 \cdot \cos(2\pi(t - \varphi_1)/24) + A_2 \cdot \cos(2\pi(t - \varphi_2)/12)$, for each state × group combination. Solid lines: observed group mean; dashed lines: cosinor fit. Gray shading = dark phase (ZT12–ZT0). Single-harmonic fits are shown as thin dotted lines for comparison. Fitted parameters (mesor, amplitude, acrophase) are in Supplementary Table S4. Group coding as in Figure 3. n = 6/group.

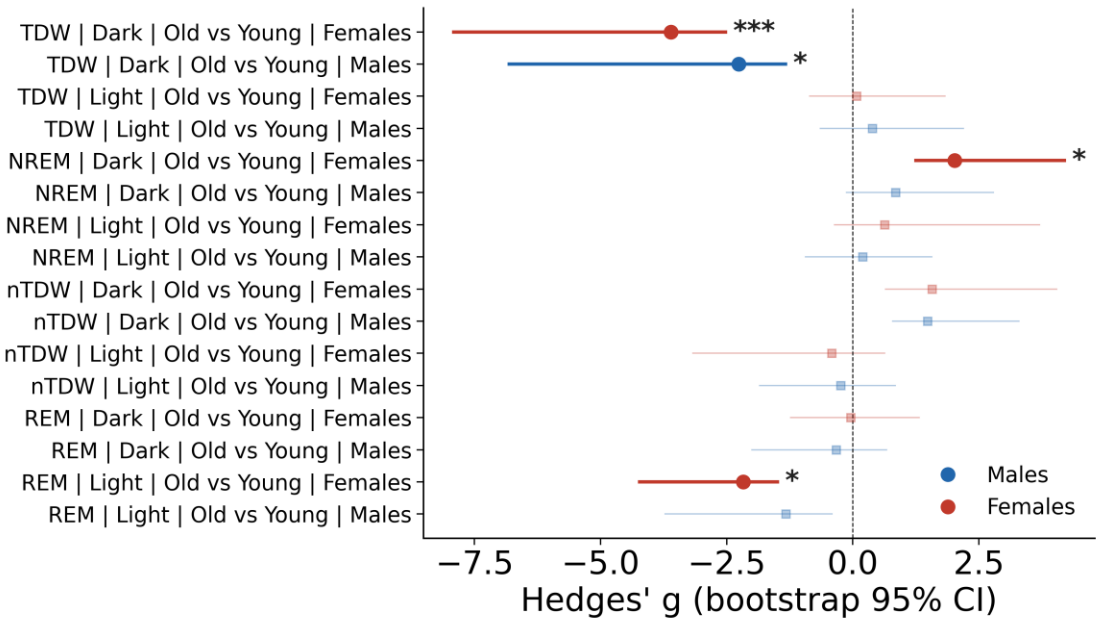


**Supplementary Figure S5. Age effects with large effect sizes cluster in dark-phase wake and in female-specific NREM and REM contrasts.** Forest plot of Hedges' g (small-sample-corrected standardized mean difference) with bootstrap 95% CIs (10,000 resamples) for all 16 between-group age contrasts: 4 vigilance states (TDW, nTDW, NREM, REM) × 2 circadian phases (dark, light) × 2 sex strata (male, female). Filled symbols: contrasts surviving Holm–Bonferroni correction within each state × phase family of 4 (dark-phase TDW both sexes; dark-phase NREM females; light-phase REM females). Open symbols: non-surviving contrasts. Dashed vertical lines: minimum detectable |d| = 1.80 at 80% power under the n = 6 design (Supplementary Appendix S1). Companion effect-size visualization to the Tier-1 phase-level inference in Figure 2. Full numerical contrasts in Supplementary Table S7. n = 6/group.

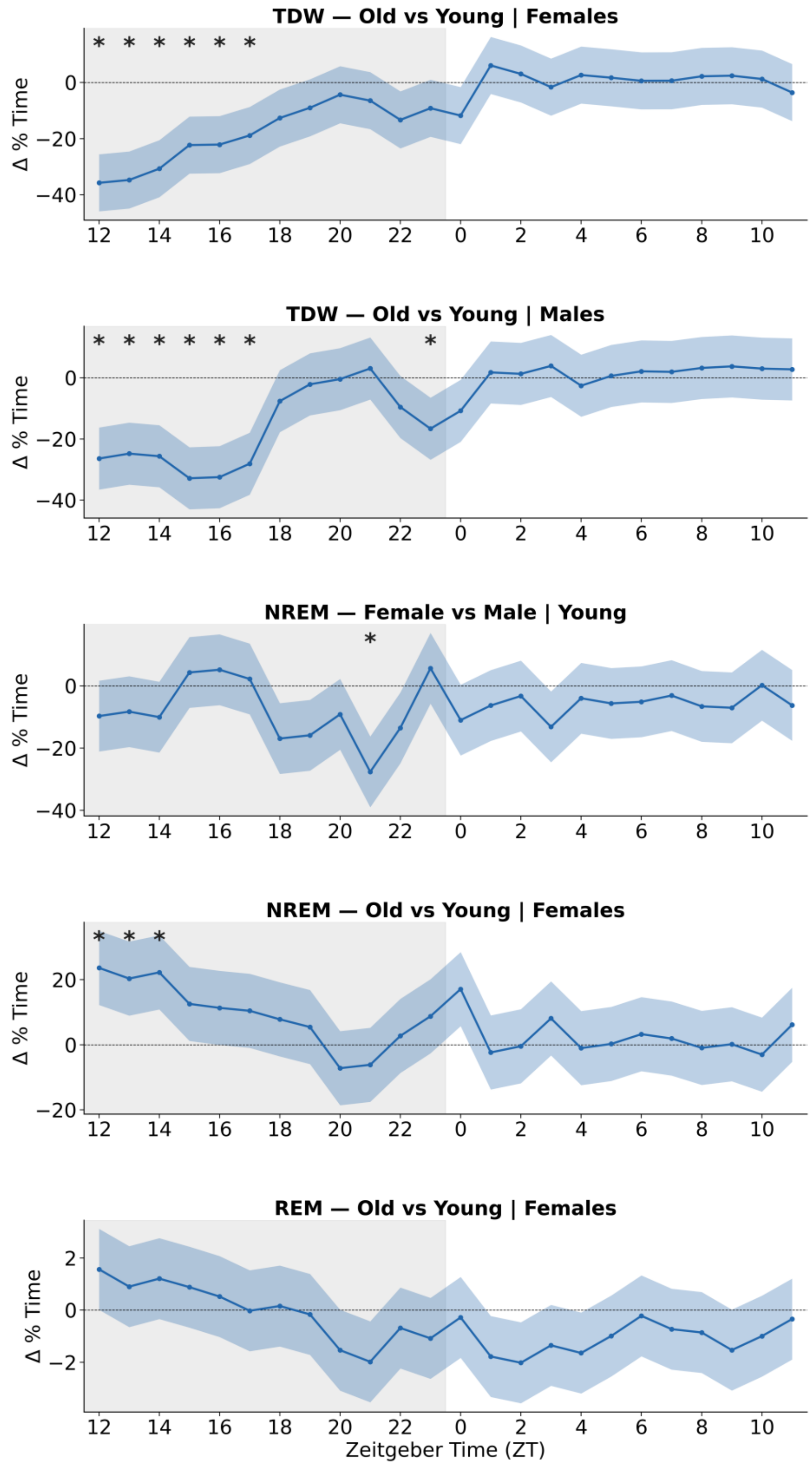
TDW — Old vs Young | Females
TDW — Old vs Young | Males
NREM — Female vs Male | Young
NREM — Old vs Young | Females
REM — Old vs Young | Females
Δ % Time
Zeitgeber Time (ZT)

**Supplementary Figure S6. Cascade-gated hourly EMM contrasts localize where within each phase the age effect peaks; 17 of 120 cells reach Holm-adjusted significance.** Tier-3 exploratory decomposition of the phase-level age effect (Section 2.6, Tier 3). For each state × ZT-hour cell where the parent phase-level Tier-1 contrast was significant, the hourly EMM contrast (Old vs. Young, pooled across sex) is plotted as mean ± 95% CI. Traces show the age-contrast time course across 24 h; cells passing the within-phase Holm-adjusted threshold (p_Holm < 0.05) are marked with an asterisk above the bin. Group coding as in Figure 3. These contrasts are descriptive localizations of when within the phase effects peak, not independent confirmatory tests. n = 6/group.

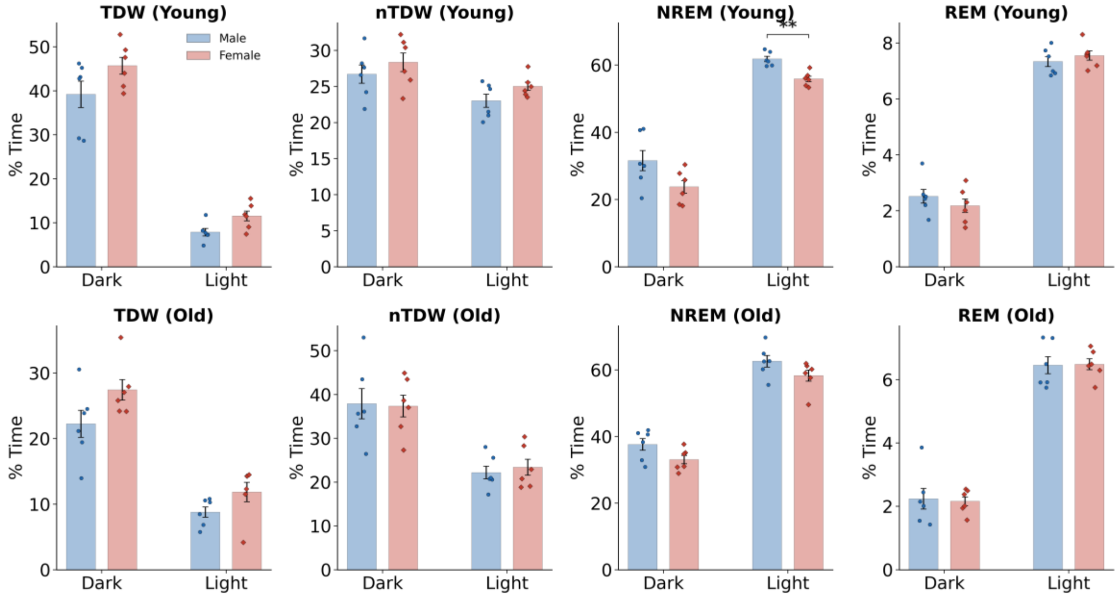


**Supplementary Figure S7. Sex contrasts are small and inconsistent across vigilance states; only light-phase NREM in young animals shows a Holm-surviving difference.** Phase-averaged (dark ZT12–ZT0, light ZT0–ZT12) vigilance-state percentages shown as per-mouse dots with group mean ± SEM, separately for TDW, nTDW, NREM, and REM. Groups: males (blue bars), females (red bars), with young-age stratum in the top row and old-age stratum in the bottom row; dots = individual animals. Horizontal brackets indicate between-sex contrasts within each age (YM vs. YF; OM vs. OF) with Holm-corrected p-values from independent-samples t-tests (df = 10); asterisks: *p_Holm < 0.05, **p_Holm < 0.01. Full contrast table in Supplementary Table S6. n = 6/group.

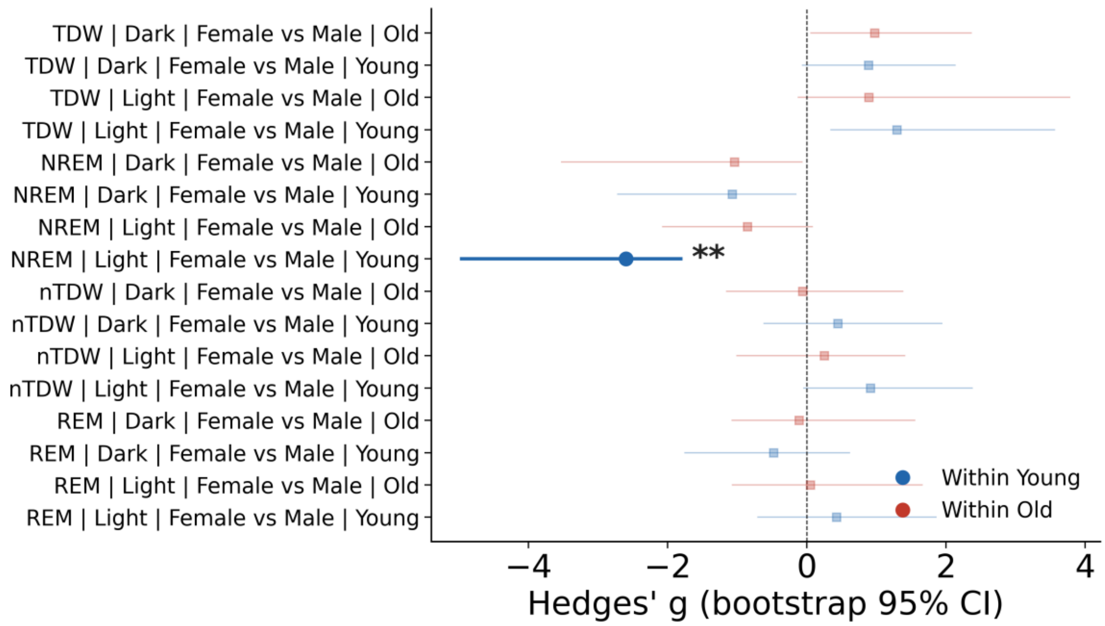


**Supplementary Figure S8. Sex effect sizes cluster near |g| = 0.9–1.4 in dark-phase wake states but have confidence intervals that cross zero after correction.** Forest plot of Hedges' g (small-sample-bias-corrected) for all 16 sex contrasts (4 states × 2 phases × 2 age strata; YM vs. YF and OM vs. OF), with bootstrap 95% CIs (10,000 resamples). Vertical dashed line at g = 0 (no difference); vertical dotted line at |g| = 1.44 marks the minimum detectable paired effect at 80% power under the present design (Section 2.6). Filled markers = Holm-surviving contrasts within state; open markers = non-surviving. Contrasts are listed in Supplementary Table S6. n = 6/group.

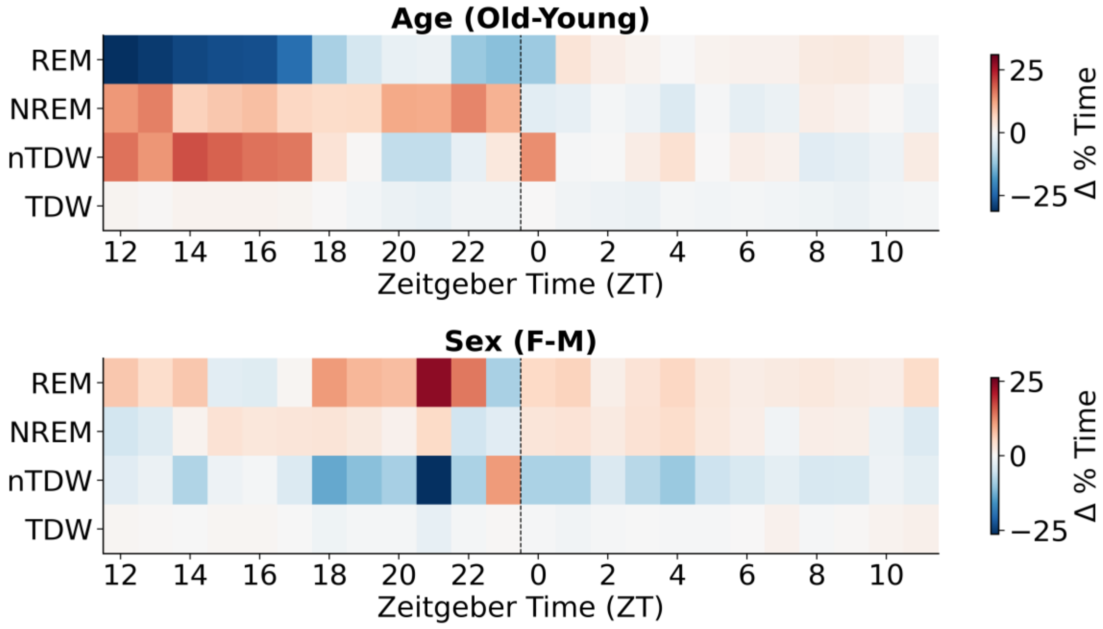


**Supplementary Figure S9. Age-related effect sizes concentrate in the early-to-mid dark phase for TDW and mirror-image for NREM, with the largest effects at ZT12–ZT18.** Top panel: Age (Old − Young) effect; bottom panel: Sex (Female − Male) effect. Heatmap of the age-related difference in state percentage (Δ%, Old − Young) for each ZT hour × vigilance state (pooled across sex). Color scale: red = Old > Young (positive Δ%), blue = Old < Young (negative Δ%); color intensity proportional to |Δ%|. The vertical dashed line demarcates the dark/light phase boundary (ZT0). These hourly effect sizes are descriptive; formal inference is reported at the phase level (Section 3.1) and at the hourly level under cascade-gated Holm correction (Supplementary Figure S6). n = 6/group.

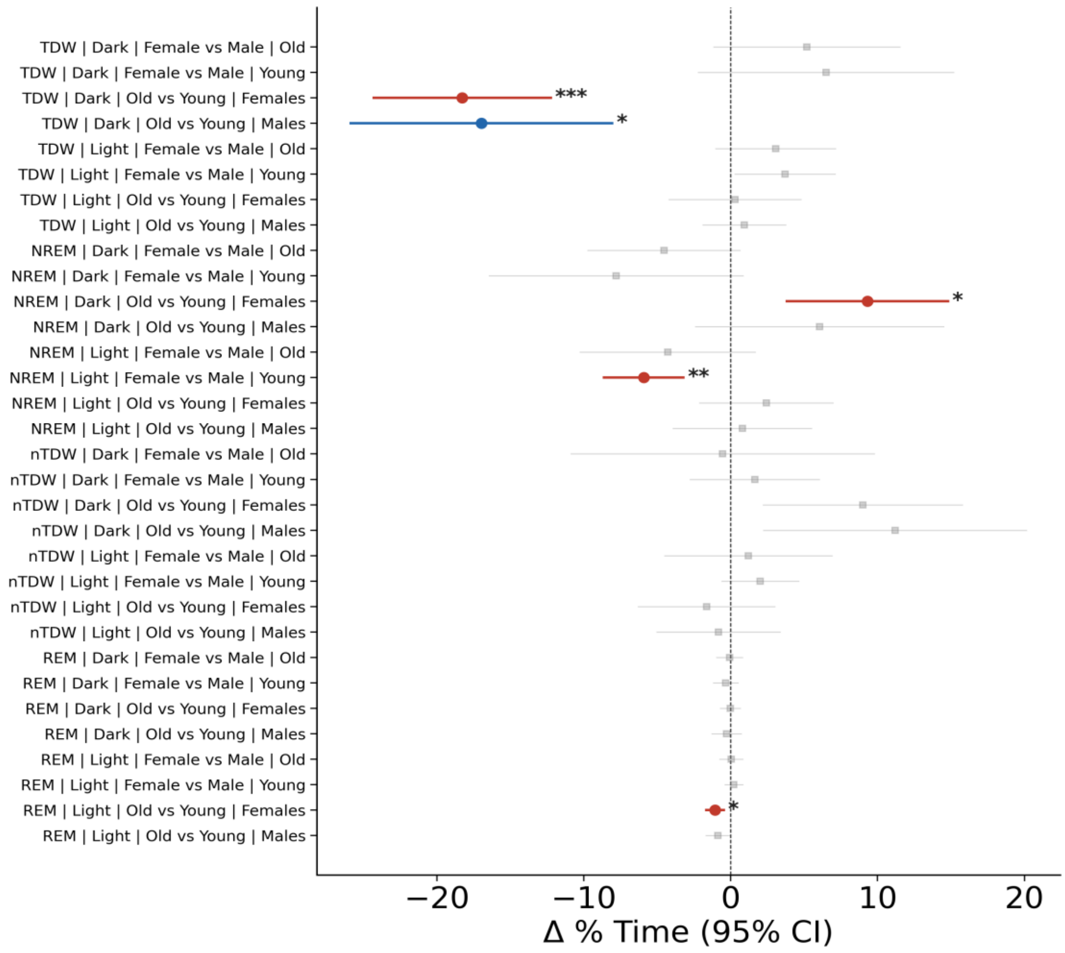


**Supplementary Figure S10. Full set of 32 phase-level between-group contrasts with bootstrap 95% CIs; four contrasts survive Holm–Bonferroni correction within state.** Forest plot of Δ% (mean between-group difference in vigilance-state percentage) for all 32 contrasts (4 states × 2 phases × 4 pair types: YM vs. OM, YF vs. OF, YM vs. YF, OM vs. OF). Error bars: bootstrap 95% CI (10,000 resamples). Filled markers: $p_{Holm} < 0.05$ within state (dark-phase TDW in both sexes, dark-phase NREM in females, light-phase REM in females); open markers: non-surviving. Vertical dashed line at Δ% = 0. Full statistics in Supplementary Table S6. n = 6/group.

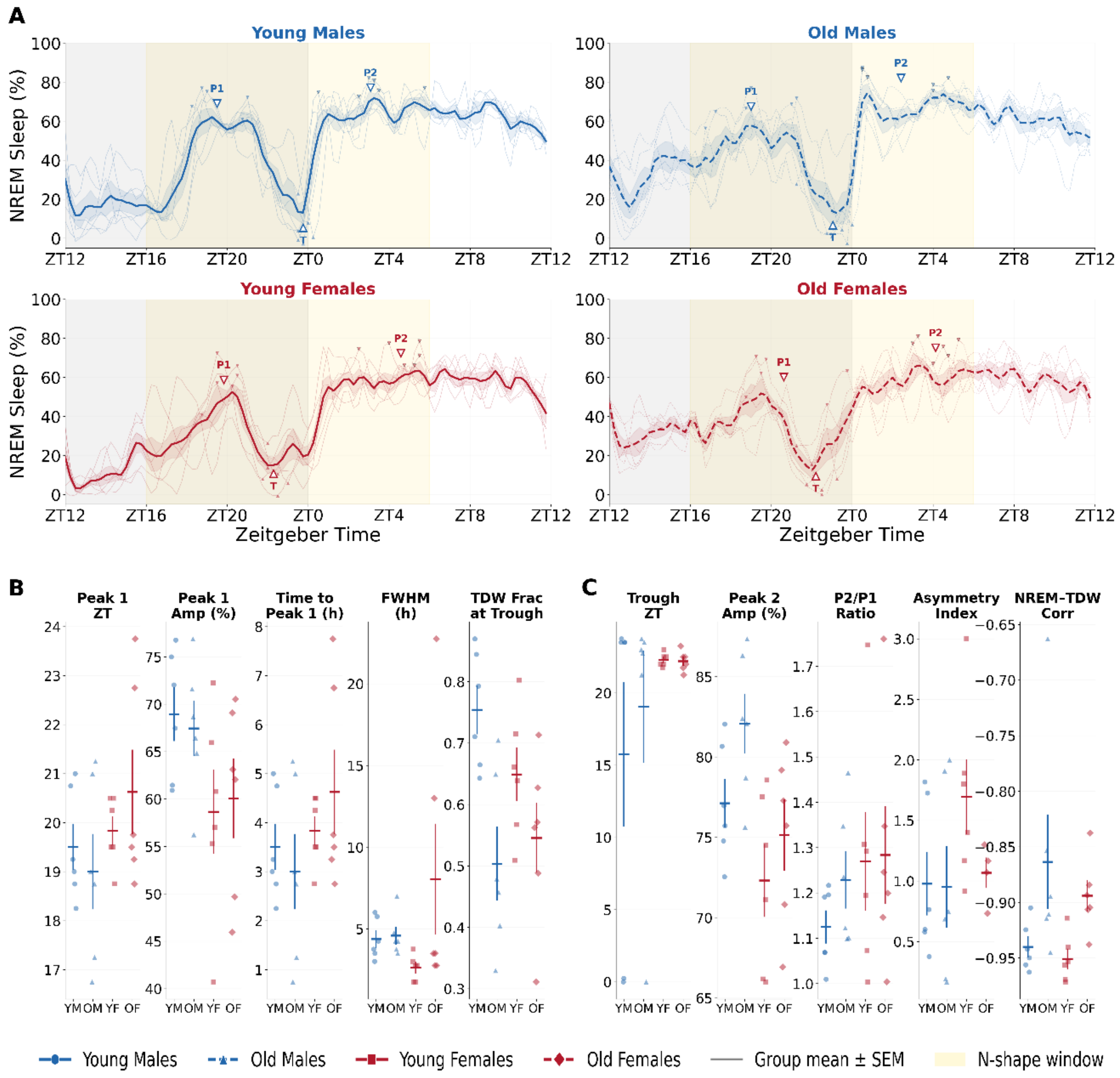


**Supplementary Figure S11. Aging reduces TDW fraction at the N-shape onset (ZT16, Tier-1 confirmatory) and at the pre-lights-on trough (Tier-2 descriptive); other N-shape landmarks are not Holm-surviving.** N-shape landmark analysis within ZT16–ZT6 (yellow shading; late-dark rise → pre-lights-on dip → early-light surge). (A) Individual NREM profiles with detected landmarks (Peak 1 ▽, trough △, Peak 2 ▽ black edge); bold lines = group means ± SEM. Group coding as in Figure 3. (B) Tier-2 landmark-based metrics (Holm-corrected): Peak 1 ZT, Peak 1 amplitude, time-to-Peak 1, FWHM, TDW fraction at the data-driven trough. Only the last survives Holm ($p = 0.002$, $d = 1.43$). (C) Tier-3 exploratory landmarks (nominal p). Bars: group means ± SEM. The Tier-1 primary endpoint — TDW fraction at the fixed pre-specified ZT16 bin — is reported in Section 3.2.3 ($F(1,20) = 26.94$, $p < 0.0001$, $g = 2.05$) and not reproduced as a landmark panel here. $n = 6$/group.

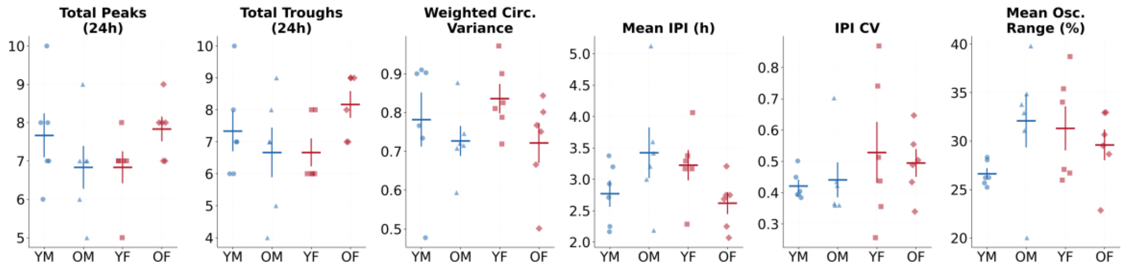


**Supplementary Figure S12. Global ultradian NREM metrics are largely preserved across Age × Sex groups; only weighted circular variance of peak timings shows a nominal age effect.** Six panels showing (A) total NREM peaks per 24 h, (B) total NREM troughs per 24 h, (C) weighted circular variance of peak timings, (D) mean inter-peak interval (IPI) in hours, (E) IPI coefficient of variation, and (F) mean peak-to-trough oscillation range (pp) for each group. Dots: per-mouse values; horizontal bars: group mean ± SEM. Group coding as in Figure 3 (YM solid blue, OM dashed blue, YF solid red, OF dashed red). Between-group age and sex effects assessed by Mann–Whitney U; all p-values nominal (Supplementary Table S8). n = 6/group.

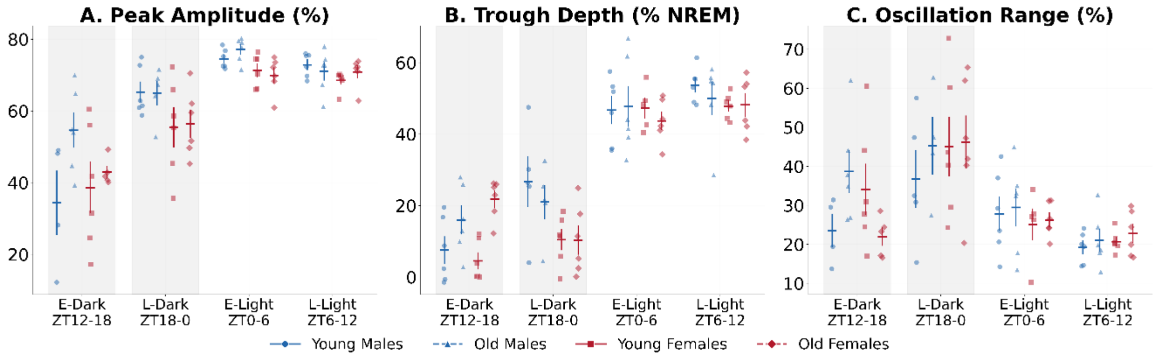


**Supplementary Figure S13. Aging deepens early-dark NREM troughs; peak amplitude is higher in males; oscillation range is preserved across groups.** Ultradian metrics from 24-h NREM profiles binned into four 6-h circadian phases (ZT12–18, ZT18–0, ZT0–6, ZT6–12). (A) Peak amplitude (% NREM). (B) Trough depth (% NREM). (C) Oscillation range (peak − trough, pp). Dots: animals; bars: group means ± SEM. Group coding as in Figure 3. Tier-3 exploratory within-phase Age × Sex ANOVAs, nominal p (Supplementary Table S11). Phase-binned TDW-fraction at troughs shown in Supplementary Figure S11. n = 6/group.

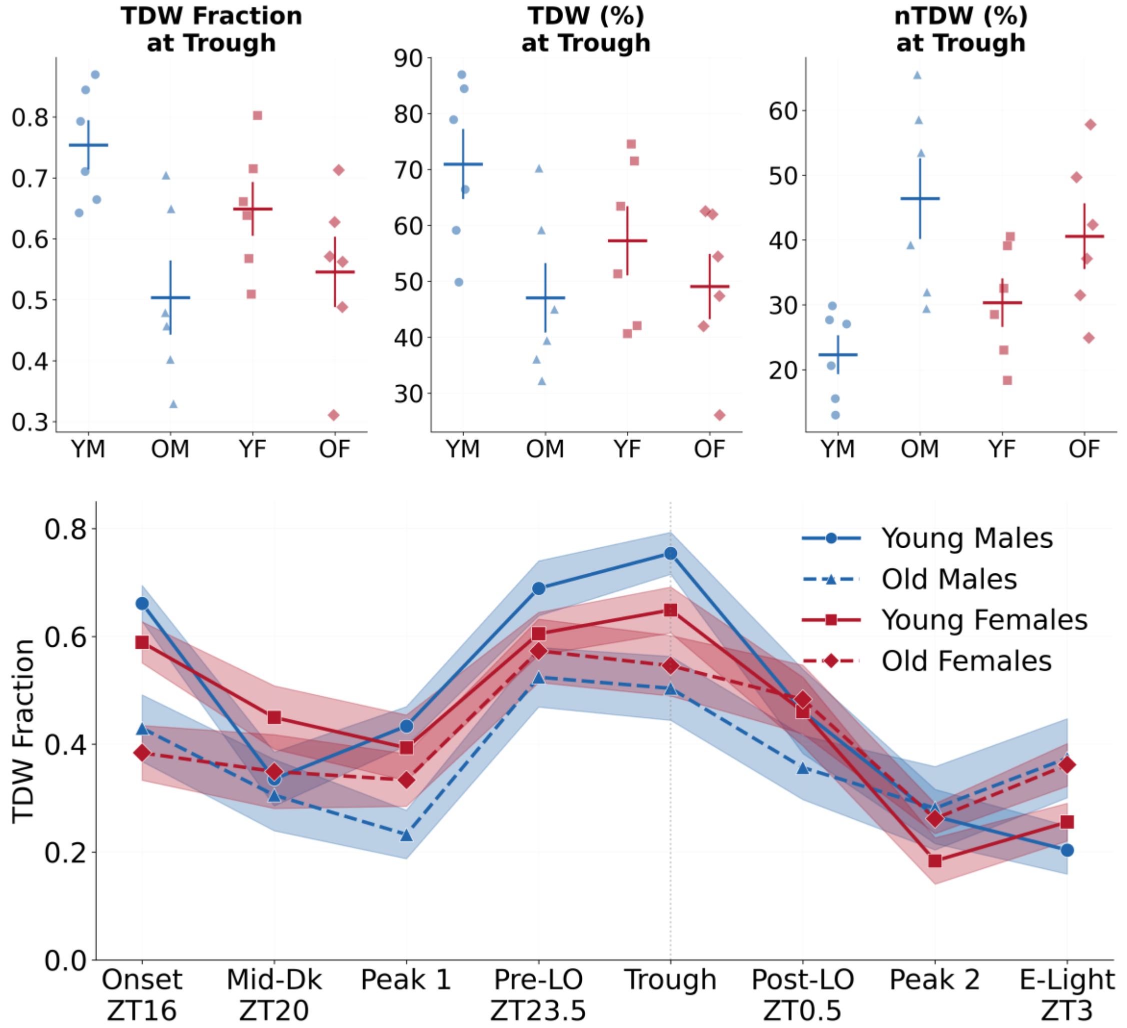


**Supplementary Figure S14. State composition at N-shape transitions: peaks are NREM-dominated, the trough is wake-dominated, and trough wake composition shifts from TDW toward nTDW with aging.** Phase-4 analyses within the ZT16–ZT6 window (Section 3.2.4). (A) Stacked bars showing NREM, REM, nTDW, and TDW percentages at Peak 1, trough, and Peak 2 for each group (group means across n = 6 mice/group). (B) NREM trajectory across eight N-shape timepoints (onset, mid-rise, Peak 1, mid-descent, trough, mid-ascent, Peak 2, post-Peak 2); lines = group means ± SEM. (C) Wake composition at the trough: TDW fraction, absolute TDW (%), absolute nTDW (%); bars = group means ± SEM, dots = per-mouse values. (D) TDW fraction trajectory across the N-shape (same eight timepoints as B). Group coding as in Figure 3. Binomial GLM on wake microcomposition at the trough in Supplementary Table S13; full contrast statistics in Supplementary Tables S11–S13. n = 6/group.

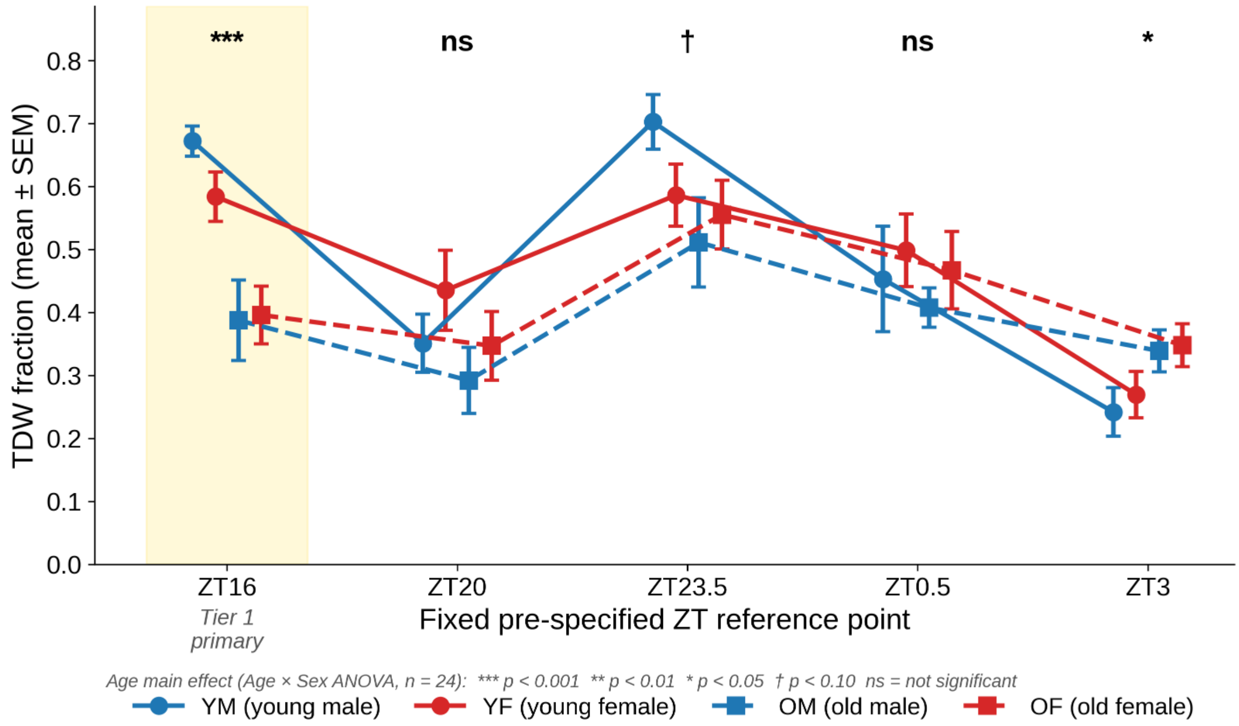


**Supplementary Figure S15. TDW fraction at the five pre-specified fixed ZT reference points.** Per-group means ± SEM of TDW fraction at each of the five fixed ZT time points pre-specified in Phase 4 (ZT16, ZT20, ZT23.5, ZT0.5, ZT3; see Methods §2.7). Group coding: YM solid blue, YF solid red, OM dashed blue, OF dashed red. Gold shading marks ZT16, designated as the Tier 1 primary confirmatory endpoint. Significance markers above each ZT point indicate the Age main effect from a factorial Age × Sex ANOVA ($n = 24$). The Age effect is strongest at ZT16 ($F(1,20) = 26.94$, $p < 0.0001$, Hedges' $g = 2.05$), borderline at ZT23.5 ($F = 4.02$, $p = 0.059$), absent at ZT20 and ZT0.5, and significant in the reversed direction (Old > Young) at ZT3 ($F = 6.01$, $p = 0.024$, Cohen's $d = -1.04$). This trajectory converges with the Tier 2 data-driven trough analysis reported in §3.2.3 ($F = 12.58$, $p = 0.002$, $d = 1.43$). Significance code: *** $p < 0.001$, ** $p < 0.01$, * $p < 0.05$, † $p < 0.10$, ns = not significant. $n = 6$ per group.

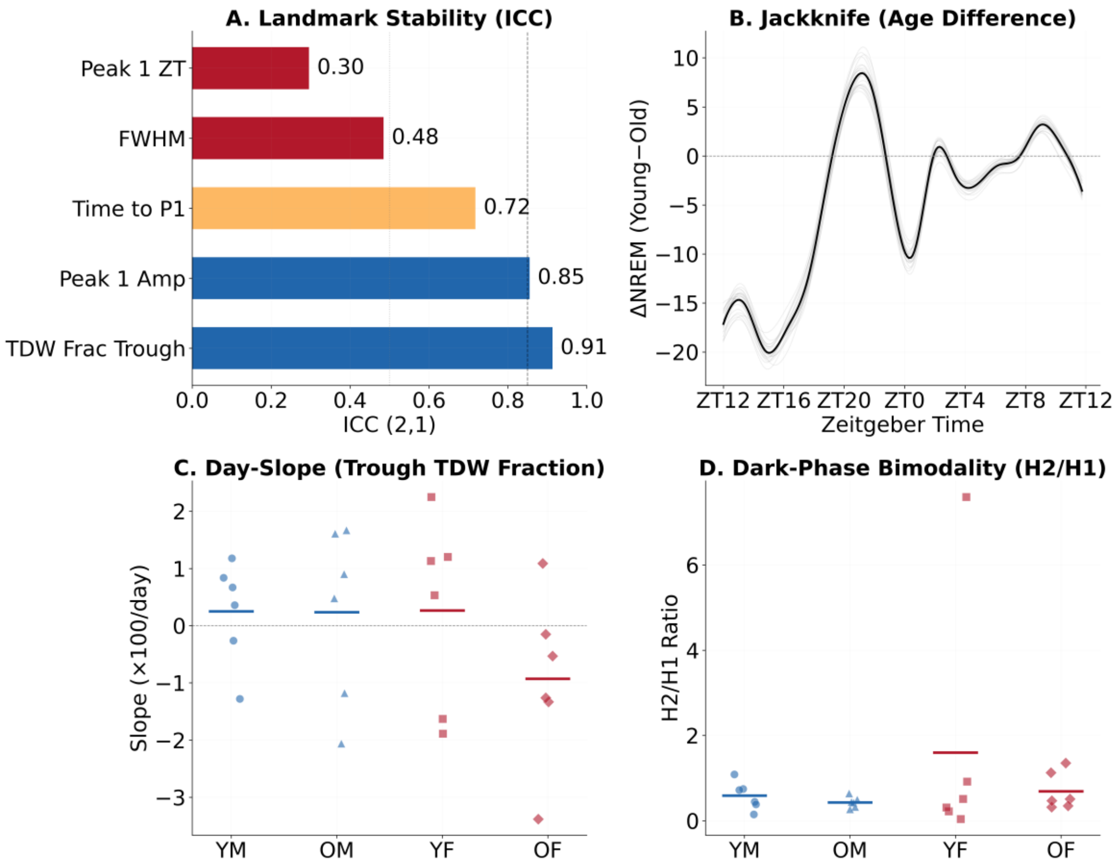


**Supplementary Figure S16. Primary N-shape landmarks are stable across preprocessing variants; the trough TDW-fraction age effect is robust to leave-one-out, day-slope, and bimodality sensitivity analyses.** Phase-5 sensitivity analyses (Section 3.2.5). (A) ICC(2,1) values for primary landmarks across nine preprocessing combinations (Savitzky–Golay window {3, 5, 7 bins} × peak prominence threshold {8%, 10%, 12%}); dashed horizontal line: ICC = 0.85 (good-stability threshold). Color code: blue = ICC ≥ 0.85 (good/excellent), orange = 0.50–0.85 (moderate), red = < 0.50 (poor). (B) Leave-one-out jackknife of the Phase-1 GAM age-difference smooth; lines show the 24 jackknife replicates (one per excluded mouse) with the full-cohort mean in bold; shaded band: envelope of LOO replicates. (C) Per-mouse linear day-slope of trough TDW fraction (units: ΔTDW-fraction per day); dots = per-mouse slopes; horizontal dashed line at zero slope. (D) Two-harmonic dark-phase bimodality index (H2/H1 = second-harmonic amplitude ÷ first-harmonic amplitude); values > 0.3 indicate bimodal structure. Group coding as in Figure 3. n = 6/group.

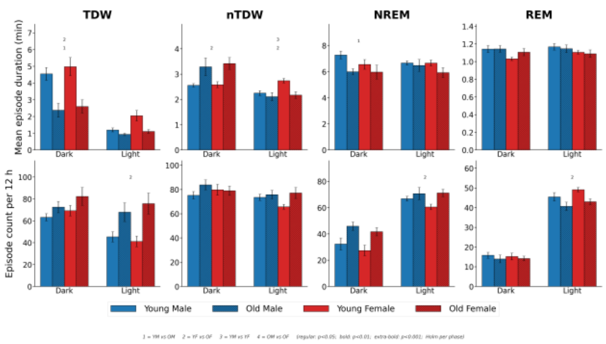


**Supplementary Figure S17. Phase-level bout duration and episode count confirm the dark-phase TDW shortening, nTDW lengthening, and light-phase NREM episode increase patterns of the hourly analysis (Figure 4).** Phase-averaged (dark ZT12–ZT0, light ZT0–ZT12) episode architecture per group. (A–D) Mean bout duration (minutes) for TDW, nTDW, NREM, and REM, respectively. (E–H) Episode count per 12-h phase for TDW, nTDW, NREM, and REM, respectively. Bars: group mean ± SEM; dots: per-mouse values. Group coding as in Figure 3 (YM solid blue, OM dashed blue, YF solid red, OF dashed red). Brackets with asterisks: independent-samples t-tests (df = 10) with Holm–Bonferroni correction within each state; *p_Holm < 0.05, **p_Holm < 0.01, ***p_Holm < 0.001. n = 6/group.

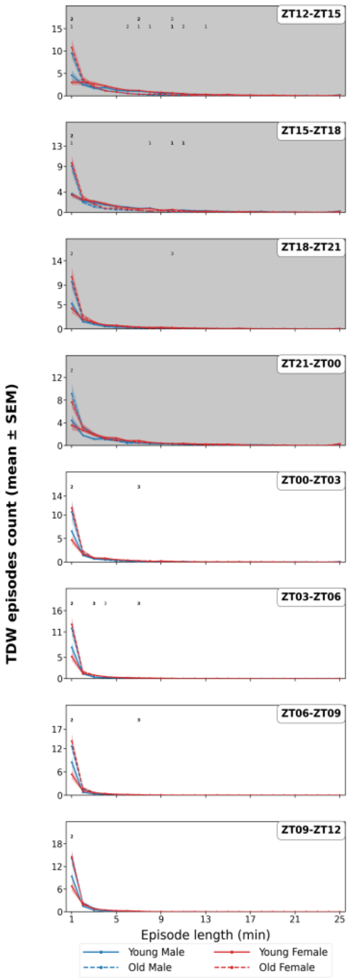


*1 = YM vs OM     2 = YF vs OF     3 = YM vs YF     4 = OM vs OF*

*regular font: q<0.05     bold: q<0.01     extra-bold: q<0.001     (BH-FDR within 8-bin family)*

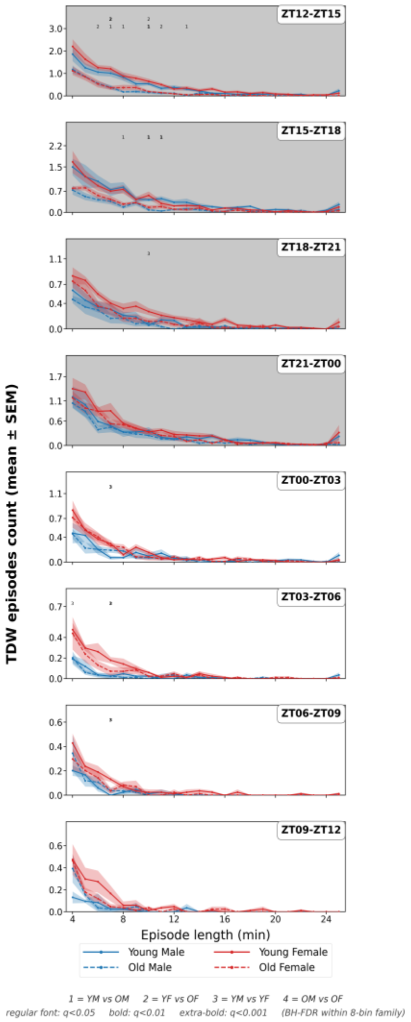


**Supplementary Figure S18. TDW episode-count distributions are bi-exponential, with aging collapsing both the short-bout and long-bout components during the dark phase.** Per-mouse distributions of TDW episode duration (1-min bins), pooled across 14 recording days. (A) Full range, 1–25 min, log-scale y-axis. (B) Zoomed view of the long-bout tail, 4–25 min, linear y-axis, to resolve the second exponential component. Solid lines: group means; shaded ribbons: ±SEM across mice. Group coding as in Figure 3. Fitted bi-exponential mixture parameters (fast component rate, slow component rate, mixing fraction) are reported in Supplementary Table S14; fits are shown for dark and light phases separately (Section 3.3.5). n = 6/group.

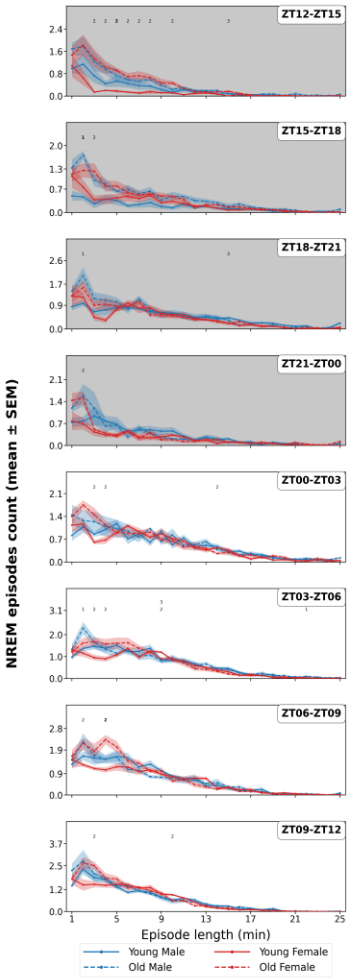


*1 = YM vs OM    2 = YF vs OF    3 = YM vs YF    4 = OM vs OF*
*regular font: q<0.05    bold: q<0.01    extra-bold: q<0.001    (BH-FDR within 8-bin family)*

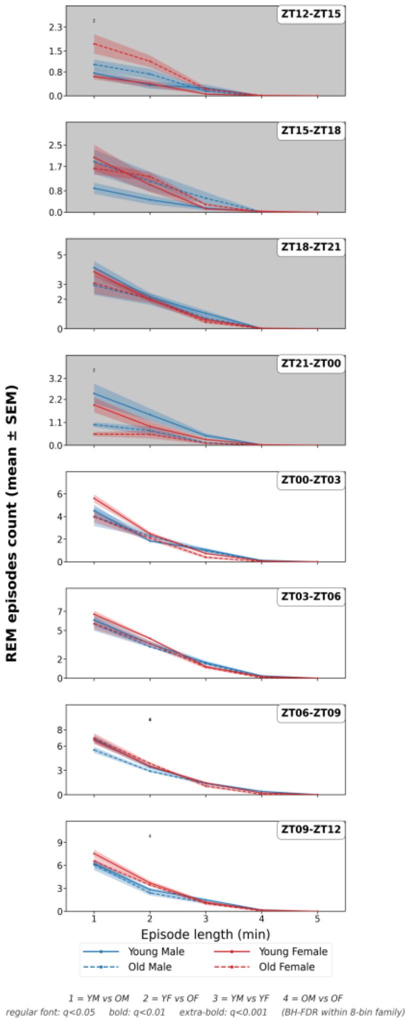


**Supplementary Figure S19. NREM and REM episode-count distributions show age-related increase in NREM episode number and largely preserved REM episode architecture.** Per-mouse distributions of (A) NREM and (B) REM episode duration (1-min bins for NREM, 30-s bins for REM), pooled across 14 recording days. Solid lines: group means; shaded ribbons: ±SEM. Group coding as in Figure 3. Phase-level contrasts in Supplementary Figure S17. n = 6/group.

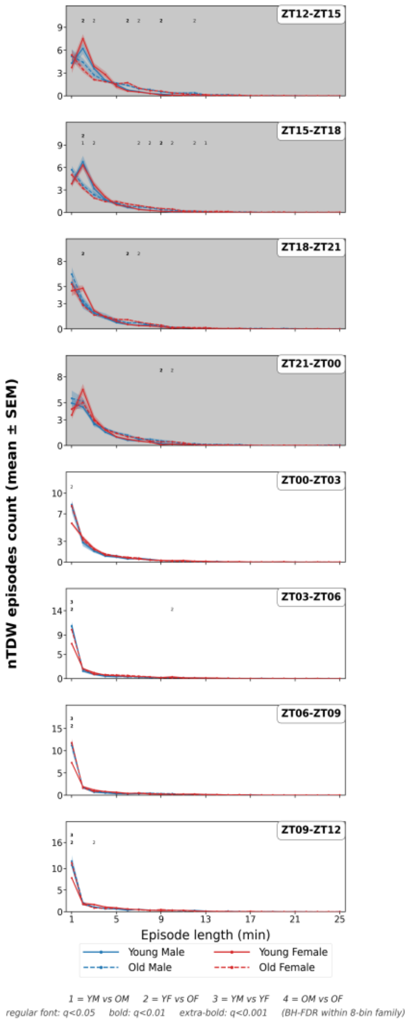
ZT12-ZT15
ZT15-ZT18
ZT18-ZT21
ZT21-ZT00
ZT00-ZT03
ZT03-ZT06
ZT06-ZT09
ZT09-ZT12
nTDW episodes count (mean ± SEM)
Episode length (min)
Young Male
Old Male
Young Female
Old Female
1 = YM vs OM 2 = YF vs OF 3 = YM vs YF 4 = OM vs OF
regular font: q<0.05 bold: q<0.01 extra-bold: q<0.001 (BH-FDR within 8-bin family)

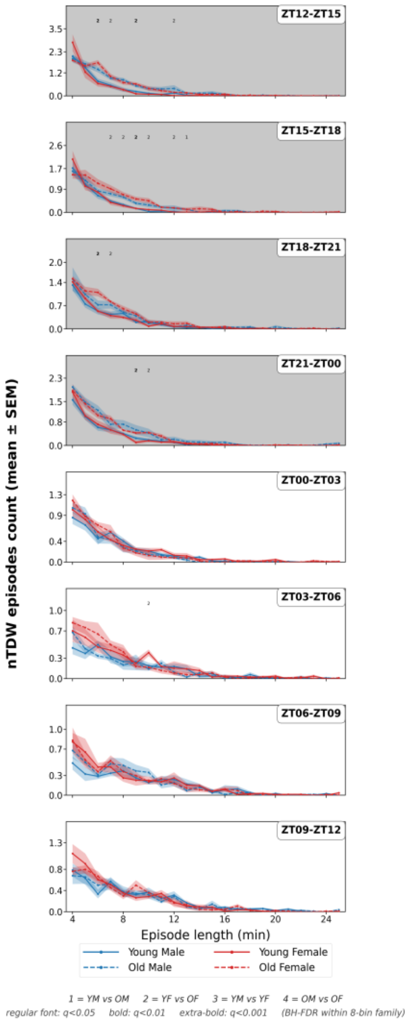


**Supplementary Figure S20. nTDW episode-count distributions lengthen with age during the dark phase, consistent with a shift from consolidated TDW toward drowsier nTDW wake.** Per-mouse distributions of nTDW episode duration (1-min bins), pooled across 14 recording days. (A) Full range, 1–25 min, log-scale y-axis. (B) Zoomed view of the long-bout tail, 4–25 min, linear y-axis. Solid lines: group means; shaded ribbons: ±SEM across mice. Group coding as in Figure 3. Phase-level contrasts in Supplementary Figure S17 and the corresponding contrast table (Section 3.3.2). n = 6/group.

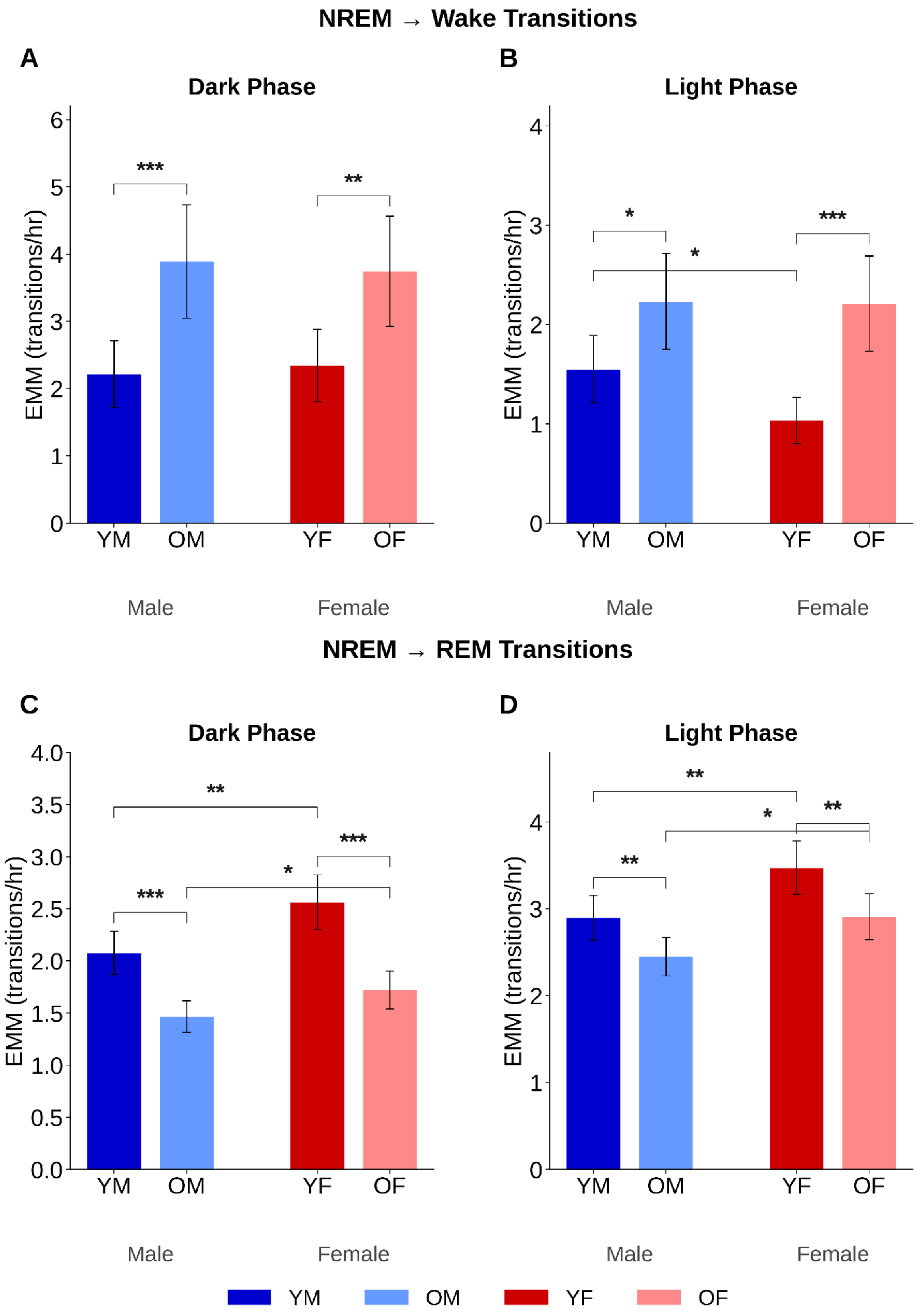
NREM → Wake Transitions
A
Dark Phase
B
Light Phase
EMM (transitions/hr)
YM
OM
YF
OF
Male
Female
NREM → REM Transitions
C
Dark Phase
D
Light Phase
YM
OM
YF
OF

Supplementary Figure S21. Phase-resolved NREM exit rates show an age-related increase in NREM→Wake and a decrease in NREM→REM, with sex differences in light-phase NREM→Wake rates converging in old animals. EMMs with 95% CIs from the Figure 5 NB GLMMs (log(NREM epochs) offset). (A, B) NREM→Wake in dark and light phases. (C, D) NREM→REM in dark and light phases. Group coding: bar-fill shade analog of the Figure 3 solid/dashed line convention — YM dark blue, OM light blue, YF dark red, OF light pink (darker fill = young, lighter fill = old, within sex). Brackets: Holm-adjusted age and sex contrasts (stars as in Figure 2). n = 6/group.

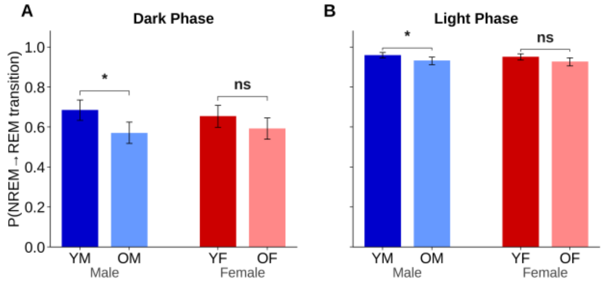


**Supplementary Figure S22. REM gating probability is reduced in aged males during the dark phase but preserved in aged females.** Model-predicted hourly probability of ≥ 1 NREM→REM transition per hour, from a binomial GLMM with fixed effects Age × Sex × Phase and log(NREM-epoch count) as an offset (Section 2.9). (A) Dark phase (ZT12–ZT0). (B) Light phase (ZT0–ZT12). Bars: predicted probability with 95% CI. Horizontal brackets: Holm-adjusted age contrasts within phase × sex. Phase main effect dominates ($\chi^2(1) = 181.5$, $p < 0.0001$). Aging reduces dark-phase gating in males (Old/Young OR = 0.61, p_Holm = 0.011*) but not females (OR = 0.77, p_Holm = 0.113, ns). Group coding as in Figure 3. n = 6/group.

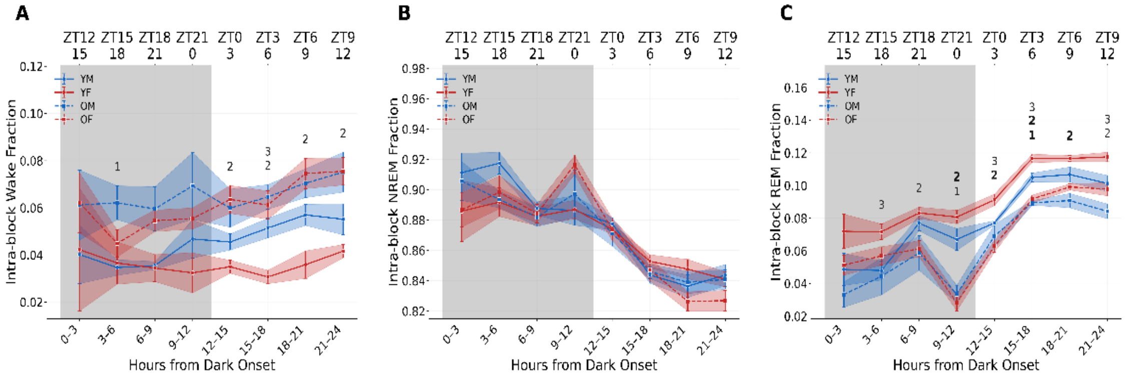


**Supplementary Figure S23. Aging raises intra-block wake fraction at the expense of NREM; young animals retain higher intra-block REM.** Per 3-h circadian bin, fraction of epochs within each ultradian block in (A) wake, (B) NREM, or (C) REM. Conventions as in Figure 6. Fractions modeled by Beta regression GLMMs. (A) Age × Sex × Bin $p < 0.001$ (largest within-bin contrast: OF vs YF light phase, $d = -4.12$). (B) Sex main effect $p = 0.007$. (C) Young > old for light-phase REM fraction. n = 6/group.

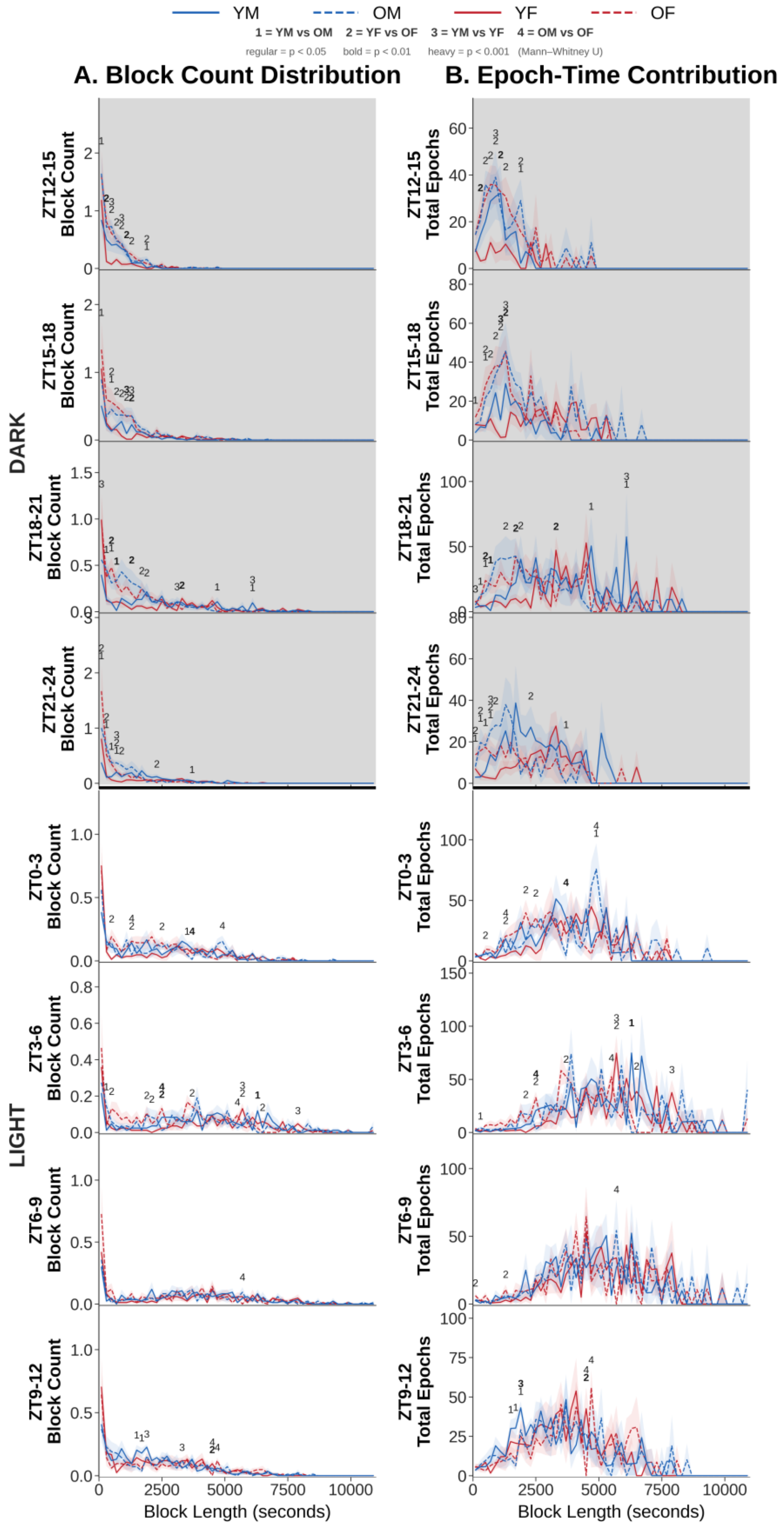

YM
OM
YF
OF
1 = YM vs OM 2 = YF vs OF 3 = YM vs YF 4 = OM vs OF
regular = p < 0.05 bold = p < 0.01 heavy = p < 0.001 (Mann–Whitney U)
A. Block Count Distribution
B. Epoch-Time Contribution
DARK
LIGHT
ZT12-15 Block Count
ZT15-18 Block Count
ZT18-21 Block Count
ZT21-24 Block Count
ZT0-3 Block Count
ZT3-6 Block Count
ZT6-9 Block Count
ZT9-12 Block Count
ZT12-15 Total Epochs
ZT15-18 Total Epochs
ZT18-21 Total Epochs
ZT21-24 Total Epochs
ZT0-3 Total Epochs
ZT3-6 Total Epochs
ZT6-9 Total Epochs
ZT9-12 Total Epochs
Block Length (seconds)
Block Length (seconds)

Supplementary Figure S24. Isolated short sleep fragments (out-blocks) accumulate with aging, cluster at <500 s, and are most frequent in old females. Block-length distribution including out-blocks — isolated single-state fragments that fail to form ultradian blocks. (A) Block count and (B) contribution to total vigilance-state time, per 200-s duration bin, across eight 3-h circadian rows (dark onset top, late light bottom; gray = dark-phase). Group coding as in Figure 3 (YM solid blue, OM dashed blue, YF solid red, OF dashed red). Numbers above curves: pairwise Mann–Whitney U (1/2/3 = $p < 0.05/0.01/0.001$). Out-block totals: OF 987, OM 780, YF 626, YM 435; Age $p = 0.003$. n = 6/group.

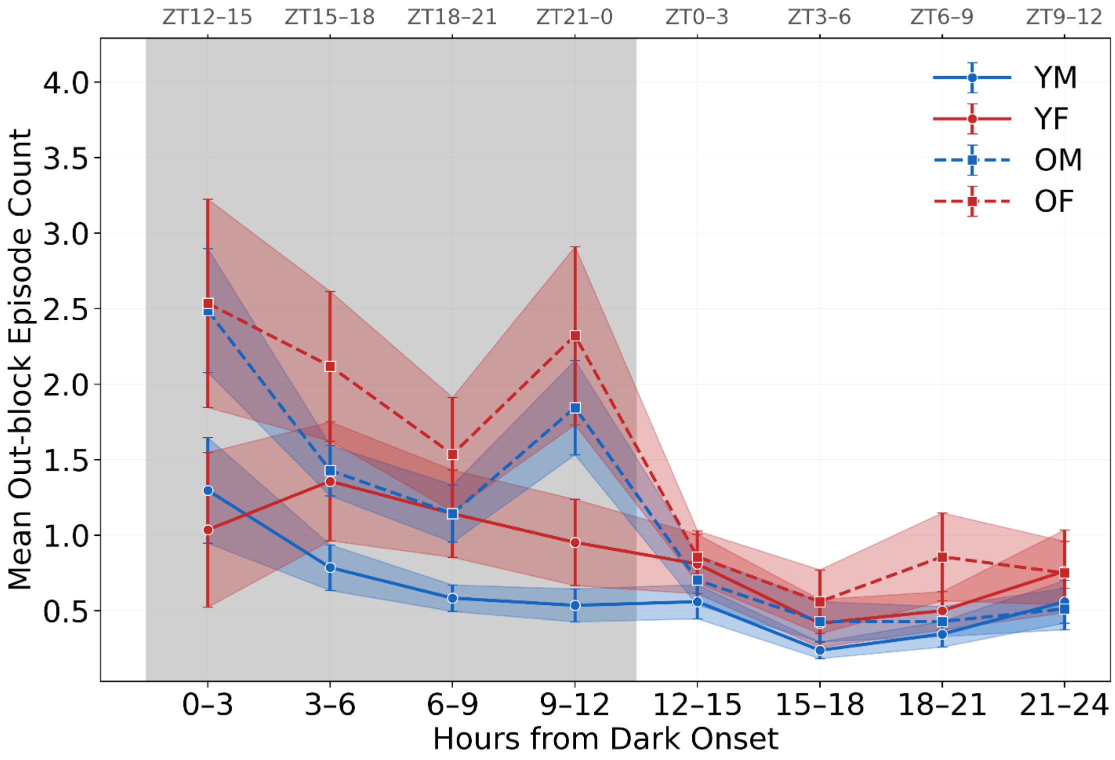


**Supplementary Figure S25. Out-block episodes accumulate disproportionately in aged females across most of the 24-h cycle, with peak density in the dark phase.** Hourly count of out-block episodes (isolated single-state sleep fragments < 500 s separated by > 180 s from neighboring sleep; Section 2.10) averaged across 14 recording days per mouse. Lines: group means ± SEM. Gray shading: dark phase (ZT12–ZT0). Group coding as in Figure 3. Age × Bin interaction $p < 0.001$ (negative binomial GLMM). Out-block totals: OF 987, OM 780, YF 626, YM 435. Hurdle-model statistics in Supplementary Table S22. n = 6/group.

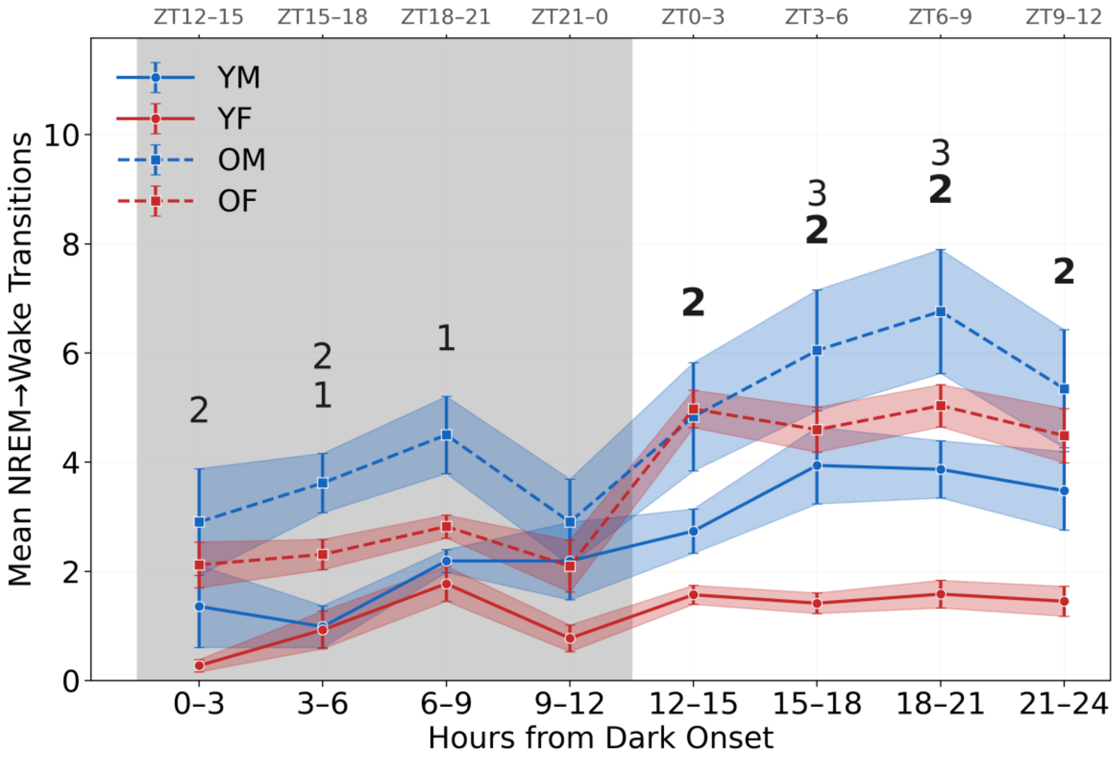


**Supplementary Figure S26. Intra-block NREM→Wake transition rate is elevated in aged mice, with the largest age gap in dark-phase old males.** Hourly count of NREM→Wake transitions occurring inside valid ultradian blocks (transitions at block boundaries excluded; Section 2.10), normalized per NREM-epoch at risk. Lines: group means ± SEM. Gray shading: dark phase. Group coding as in Figure 3. Negative binomial GLMM with log(NREM-epochs) offset: Age × Phase interaction $\chi^2(1) = 13.0$, $p = 0.0003$ (Supplementary Table S16). n = 6/group.

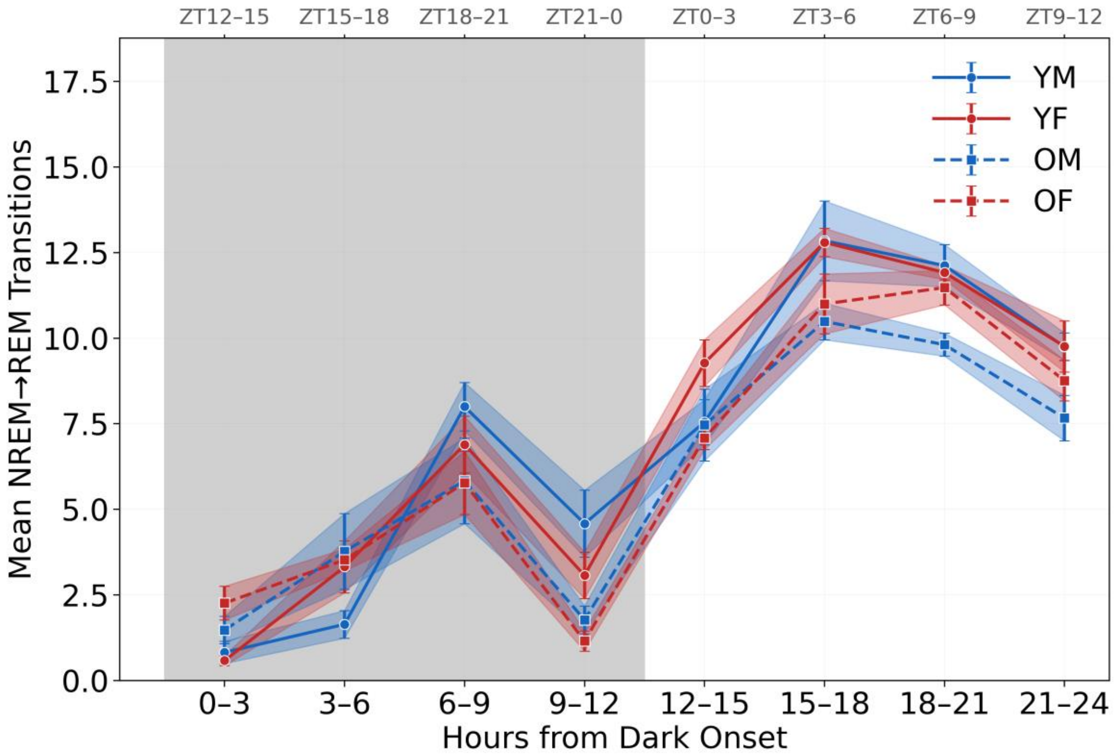


**Supplementary Figure S27. Intra-block NREM→REM transition rate is reduced in aged mice, most prominently during the dark phase.** Hourly count of NREM→REM transitions occurring inside valid ultradian blocks (Section 2.10), normalized per NREM-epoch at risk. Lines: group means ± SEM. Gray shading: dark phase. Group coding as in Figure 3. Negative binomial GLMM with log(NREM-epochs) offset: Age × Phase interaction $\chi^2(1) = 14.2$, $p = 0.0002$ (Supplementary Table S18). n = 6/group.

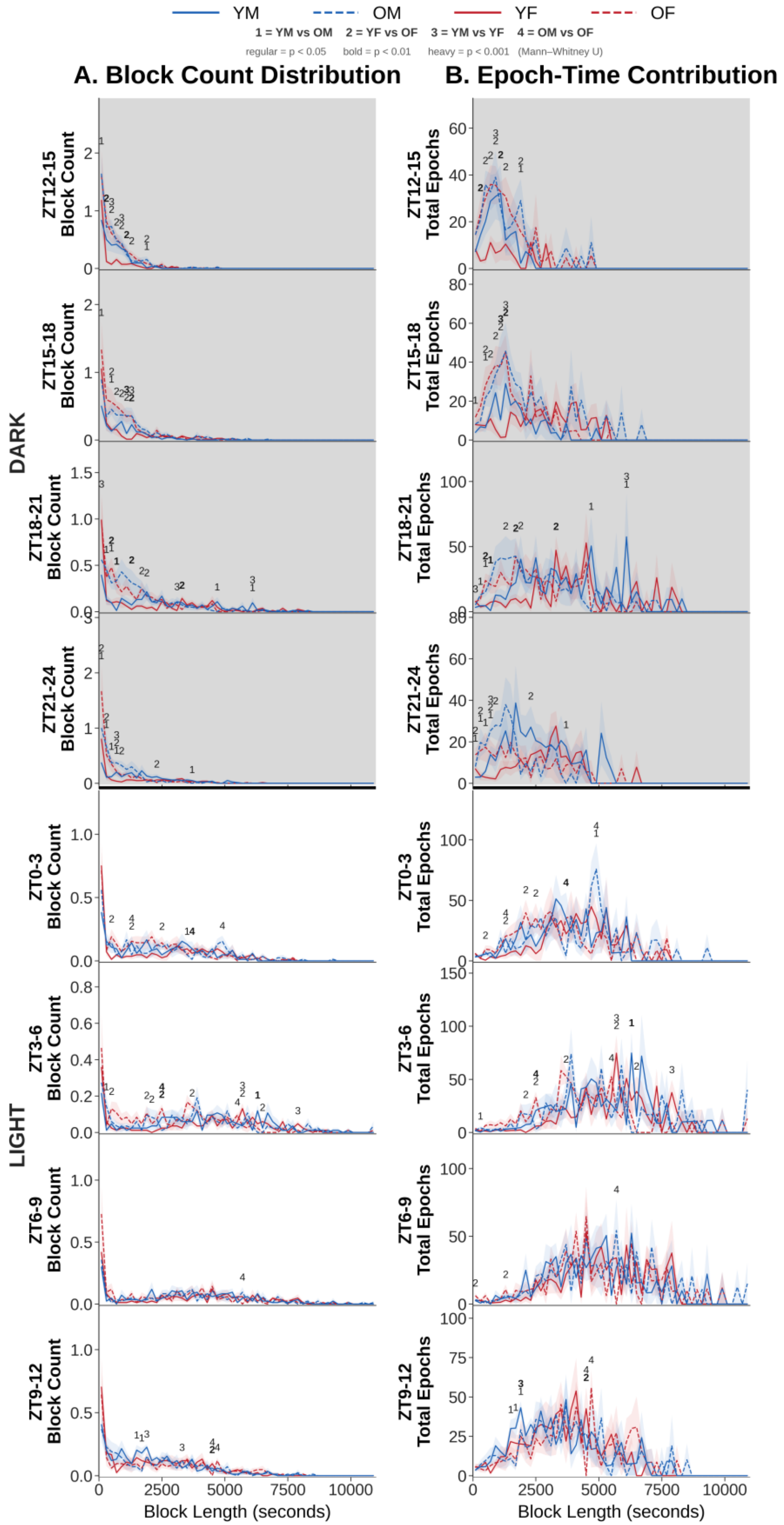
YM
OM
YF
OF
1 = YM vs OM 2 = YF vs OF 3 = YM vs YF 4 = OM vs OF
regular = p < 0.05 bold = p < 0.01 heavy = p < 0.001 (Mann–Whitney U)
A. Block Count Distribution
B. Epoch-Time Contribution
DARK
LIGHT
ZT12-15 Block Count
ZT15-18 Block Count
ZT18-21 Block Count
ZT21-24 Block Count
ZT0-3 Block Count
ZT3-6 Block Count
ZT6-9 Block Count
ZT9-12 Block Count
ZT12-15 Total Epochs
ZT15-18 Total Epochs
ZT18-21 Total Epochs
ZT21-24 Total Epochs
ZT0-3 Total Epochs
ZT3-6 Total Epochs
ZT6-9 Total Epochs
ZT9-12 Total Epochs
Block Length (seconds)
Block Length (seconds)

**Supplementary Figure S28. Valid ultradian blocks are shorter and less consolidated in aged mice during the dark phase; the distribution shifts toward shorter blocks with aging.** Distribution of valid ultradian block durations (isolated out-blocks excluded; Section 2.10), binned at 200-s resolution. (A) Block count per duration bin, across eight 3-h circadian rows (dark onset top, late light bottom; gray shading = dark-phase rows). (B) Contribution of each duration bin to total block-time. Group coding as in Figure 3 (YM solid blue, OM dashed blue, YF solid red, OF dashed red). Numbers above curves: pairwise Mann–Whitney U (1/2/3 = p < 0.05/0.01/0.001). Total valid blocks: 5,333. n = 6/group.

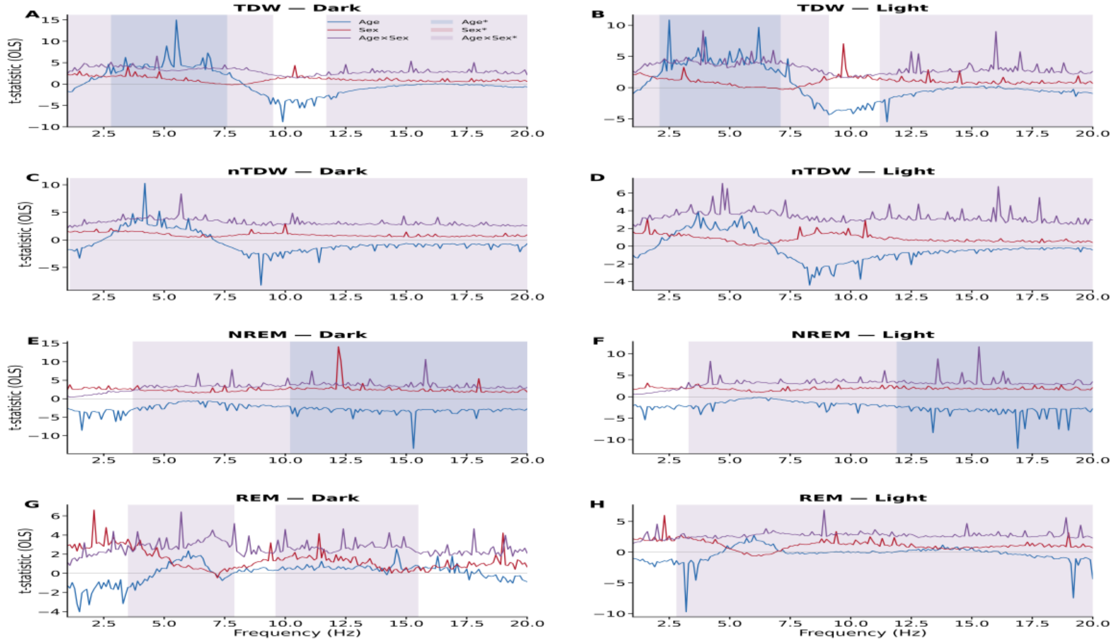


**Supplementary Figure S29. Frequency-resolved t-statistic maps confirm that the CBPT-defined clusters coincide with the local maxima of effect-size density.** Continuous t-statistic profiles from bin-wise linear models on absolute PSD (1–20 Hz at 0.1-Hz resolution) for Age (top row, red), Sex (middle row, blue), and Age × Sex (bottom row, purple) effects, across all state × phase panels (TDW, nTDW, NREM, REM × dark, light). Horizontal bars at the top of each panel mark CBPT-significant clusters (p_FWER < 0.05, 5,000 permutations). Gray shaded regions indicate the cluster-forming threshold zone (−2.086 < t < 2.086, corresponding to two-tailed α = 0.05 with df = 20). Cluster statistics in Supplementary Table S23. n = 6/group.

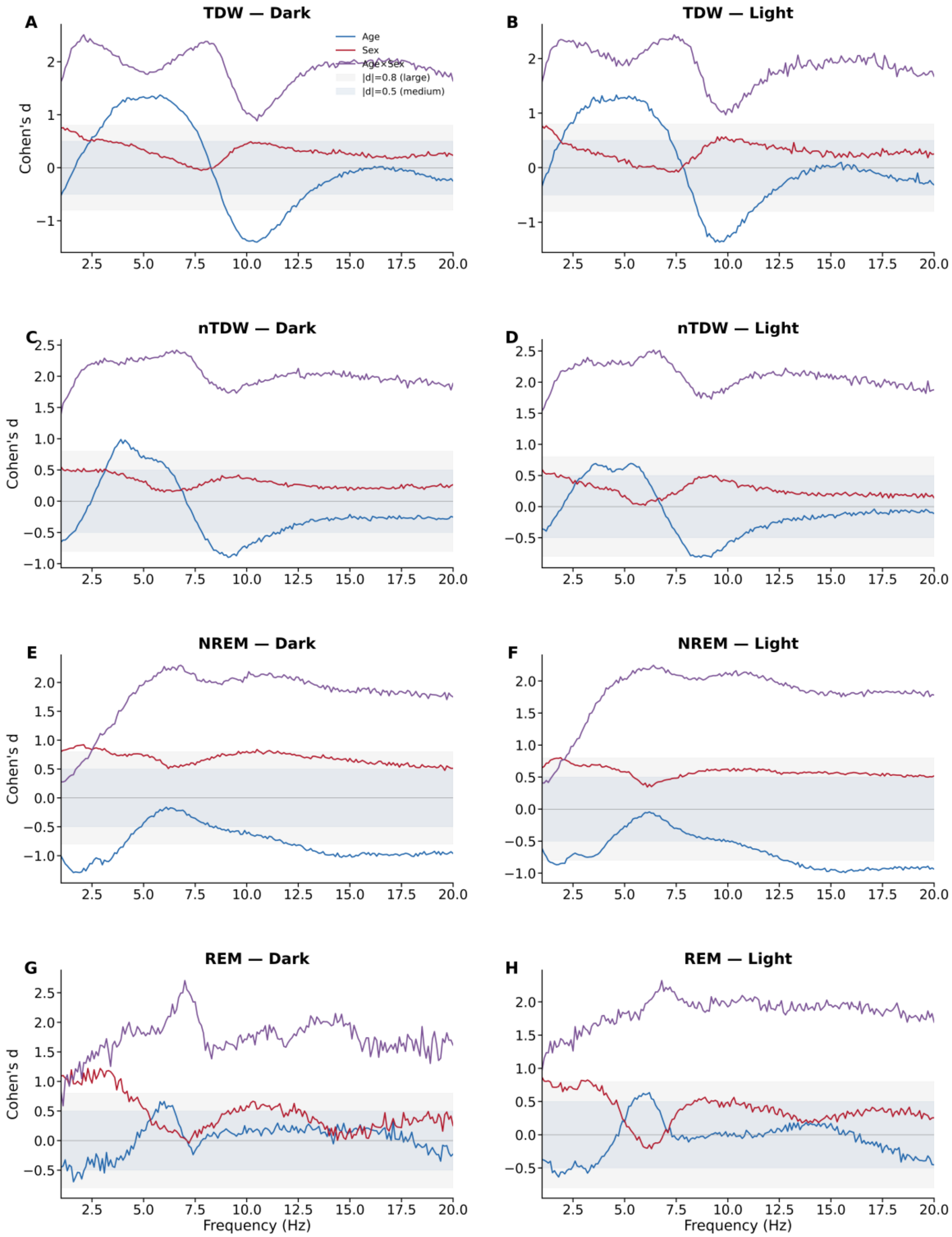


**Supplementary Figure S30. Age effects on absolute PSD reach |Cohen's d| > 0.8 in wake-state theta bands and in NREM sigma, with the crossed group contrast (OF vs. YM) showing the largest phenotypic separation.** Frequency-resolved Cohen's d for Age (old vs. young pooled across sex; red), Sex (female vs. male pooled across age; blue), and the crossed group contrast (OF vs. YM; purple) across all state × phase panels. The crossed contrast conflates main effects with the interaction and is shown for descriptive

phenotype-mapping only; it should not be interpreted as an interaction effect size. Dashed horizontal lines mark |d| = 0.8 (conventional large-effect threshold). Shaded vertical regions denote frequency ranges of CBPT-significant clusters from the primary analysis (Supplementary Table S23). n = 6/group.

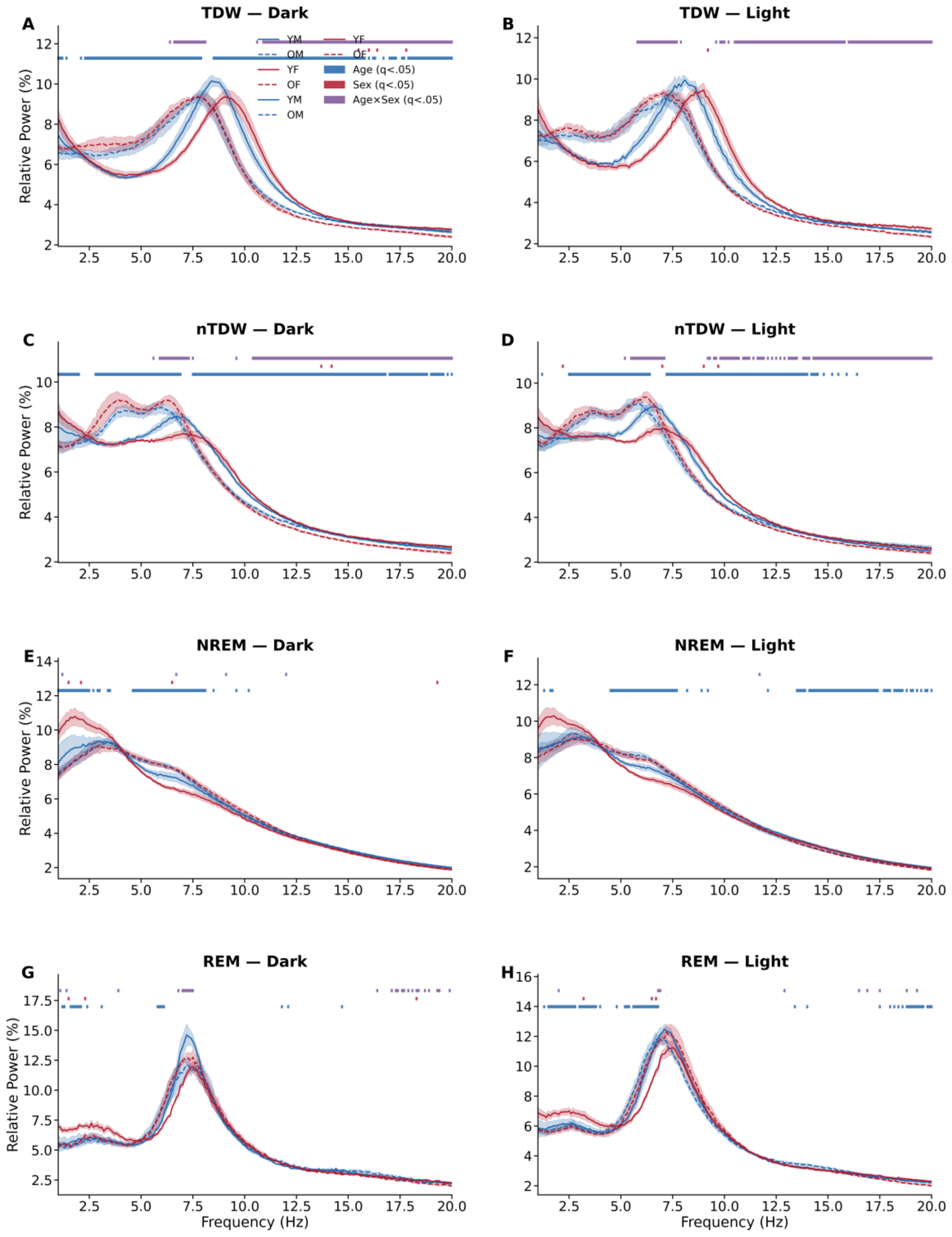


**Supplementary Figure S31. Relative PSD reveals that age effects remain frequency-selective in wake states (FDR-significant in ~90% of bins in TDW-dark and nTDW-**

**dark) but are more restricted in NREM, where much of the broadband age effect in absolute PSD reflects global scaling.** Group-averaged relative PSD (each frequency bin expressed as a proportion of the total 1–20 Hz power) for the four experimental groups across all state × phase panels (companion to the absolute-PSD primary analysis in Figure 7). Colored horizontal bars below each panel indicate frequency ranges with BH-FDR-significant effects from bin-wise linear models ($q < 0.05$): red = Age, blue = Sex, purple = Age × Sex. Group coding as in Figure 7. n = 6/group.

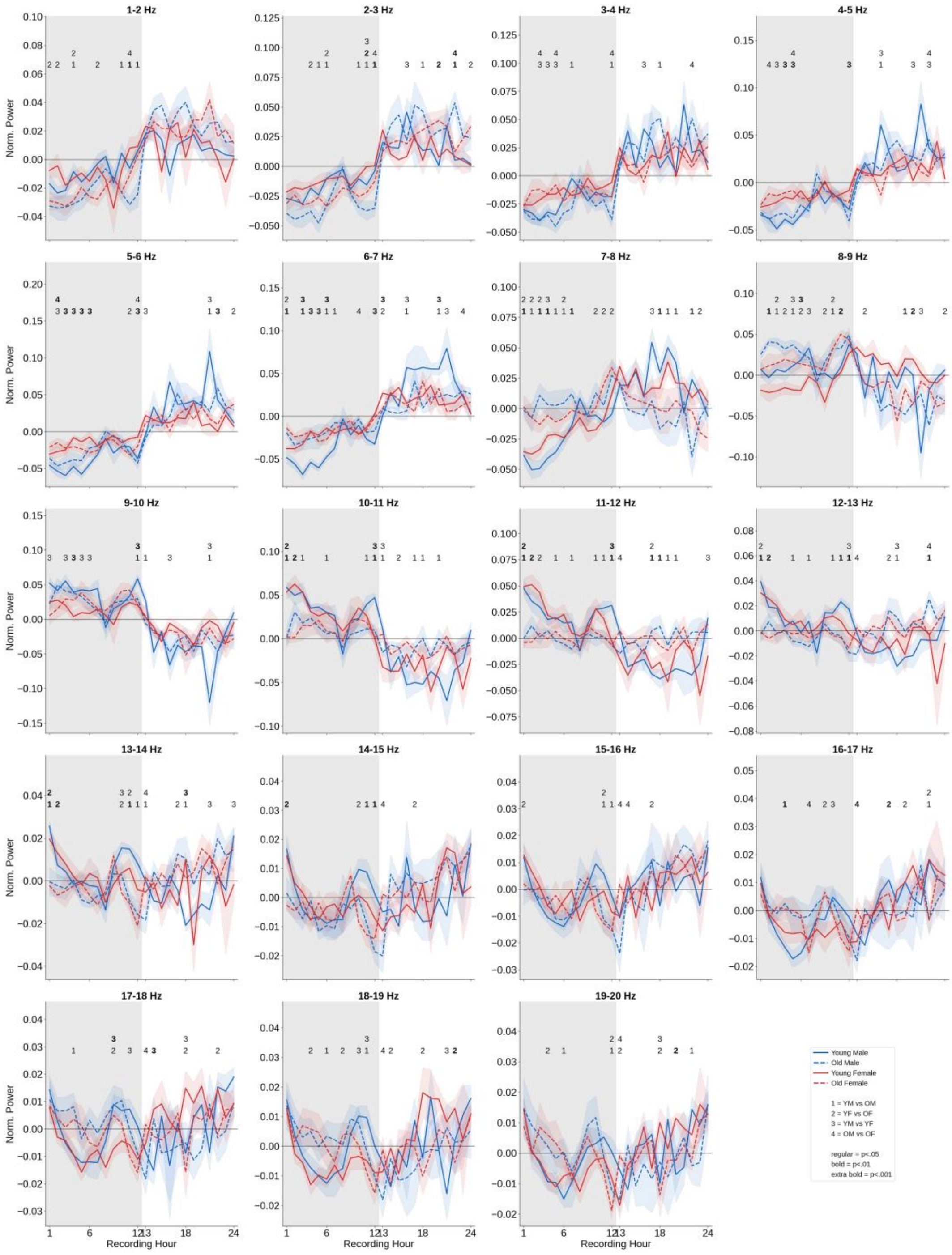


**Supplementary Figure S32. Hourly time-course of normalized EEG spectral power during TDW shows the largest age-related redistribution at dark-phase onset (ZT12) in the sigma/high-alpha range (10–13 Hz).** *Values are expressed as $log_{10}$(hourly power / 24-hour mean power) for each of 19 1-Hz bands across 24 ZT hours (456 cells per state). Lines: group means ± SEM (n = 6/group). YM solid blue; OM dashed blue; YF solid red; OF dashed red. Gray shading: dark phase (hours 1–12, ZT12–ZT0). Pairwise contrasts (Welch's t-test, uncorrected): 1 = YM vs. OM, 2 = YF vs. OF, 3 = YM vs. YF, 4 = OM vs. OF; significance*

*encoded by font weight — regular = p < 0.05, bold = p < 0.01, extra-bold = p < 0.001. FDR-corrected q-values for ANOVA cells in Supplementary Tables S24–S26. n = 6/group.*

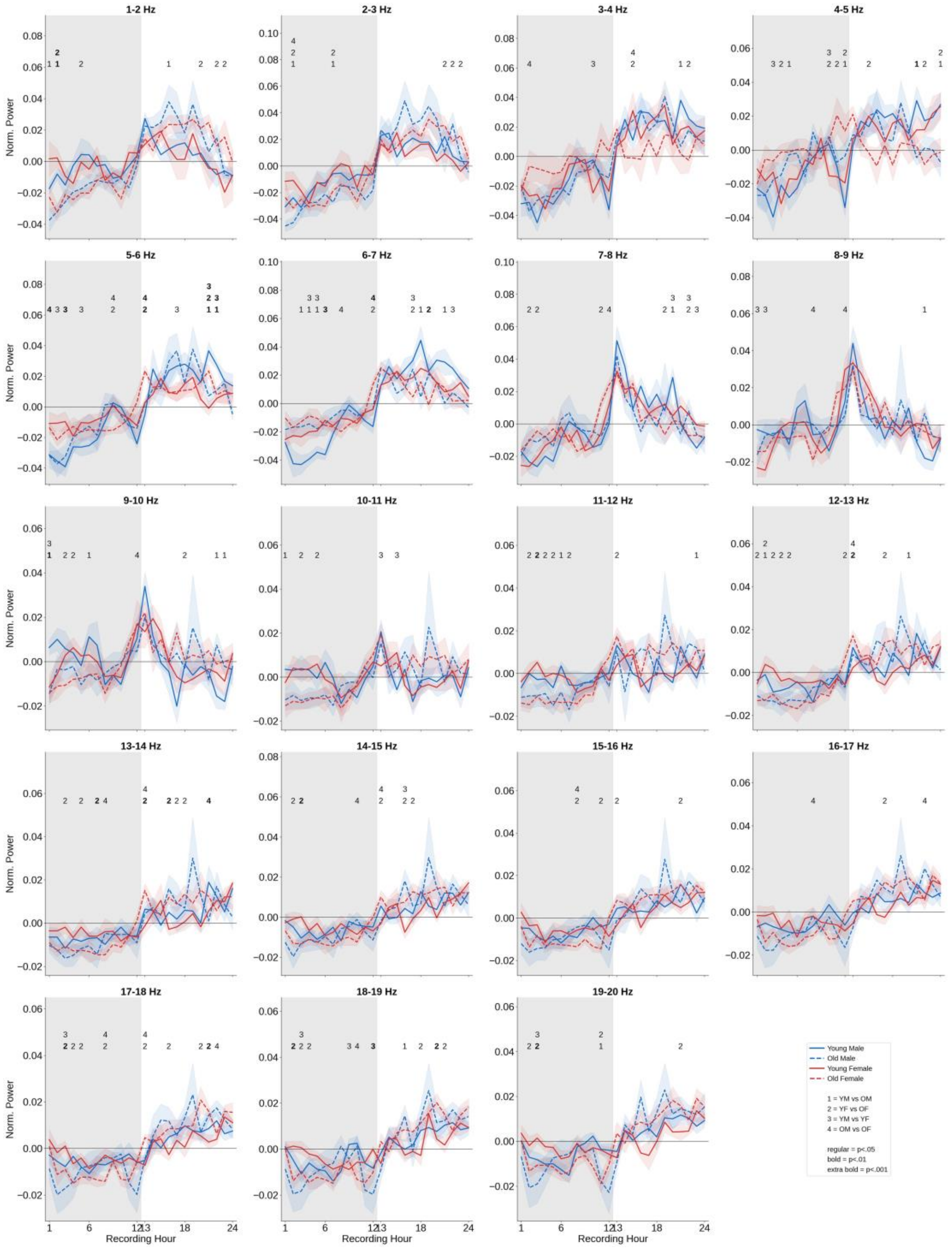


**Supplementary Figure S33. Hourly time-course of normalized EEG spectral power during nTDW; early-dark 1–2 Hz attenuation is the dominant age effect.** *Values are expressed as* $log_{10}$*(hourly power / 24-hour mean power) for each of 19 1-Hz bands across 24 ZT hours. Lines: group means ± SEM. YM solid blue; OM dashed blue; YF solid red; OF dashed red. Gray shading: dark phase (hours 1–12, ZT12–ZT0). Pairwise contrasts (Welch's t-test,*

*uncorrected): 1 = YM vs. OM, 2 = YF vs. OF, 3 = YM vs. YF, 4 = OM vs. OF; significance encoded by font weight as in Supplementary Figure S32. FDR-corrected q-values for ANOVA cells in Supplementary Tables S24–S26. n = 6/group.*

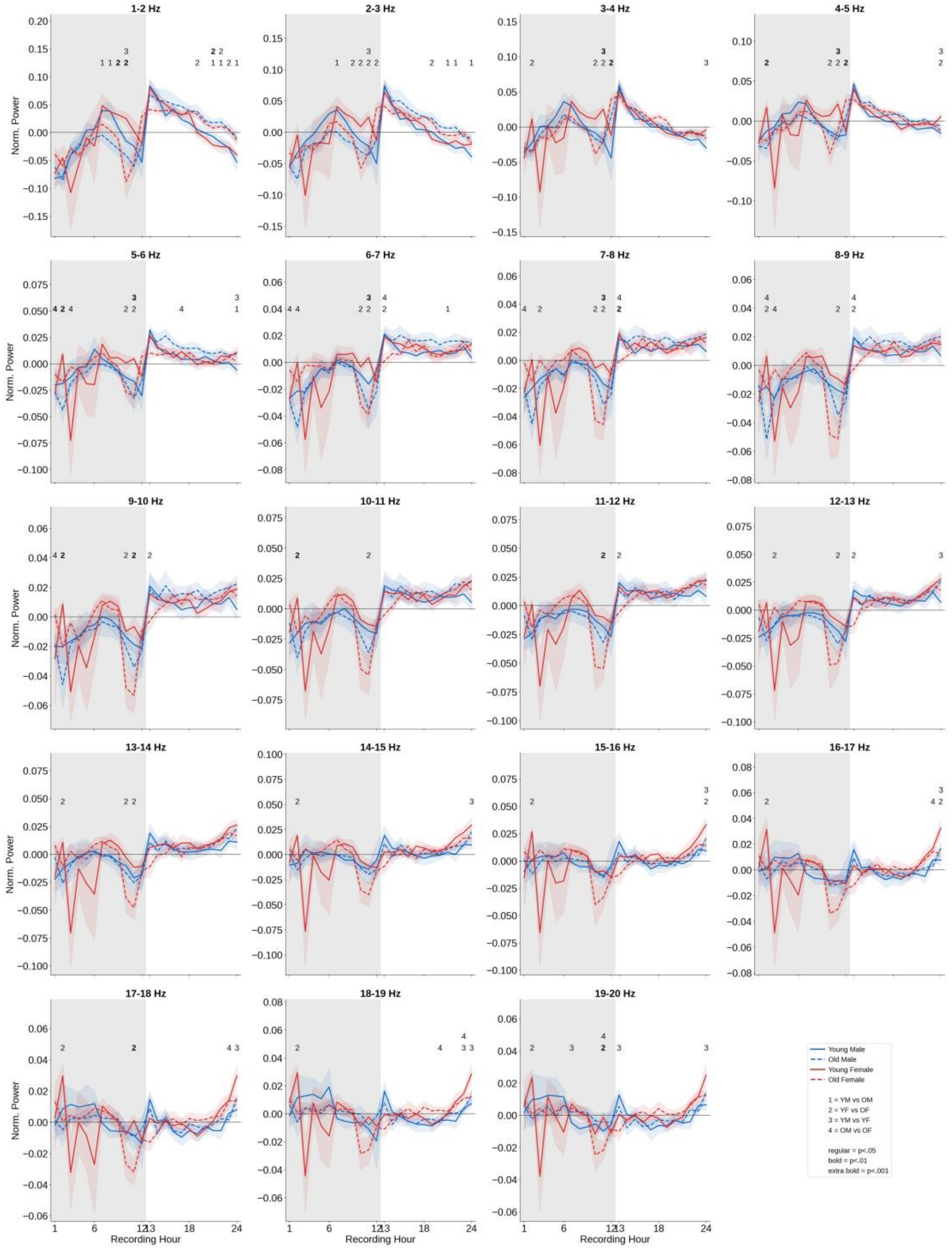


**Supplementary Figure S34. Hourly time-course of normalized EEG spectral power during NREM; age-related changes concentrate in late-dark delta (3–4 Hz) and early-light low frequencies (1–2 Hz).** *Values are expressed as $log_{10}$(hourly power / 24-hour mean power) for each of 19 1-Hz bands across 24 ZT hours. Lines: group means ± SEM. Group*

*coding and annotation conventions as in Supplementary Figure S32. FDR-corrected q-values for ANOVA cells in Supplementary Tables S24–S26. n = 6/group.*

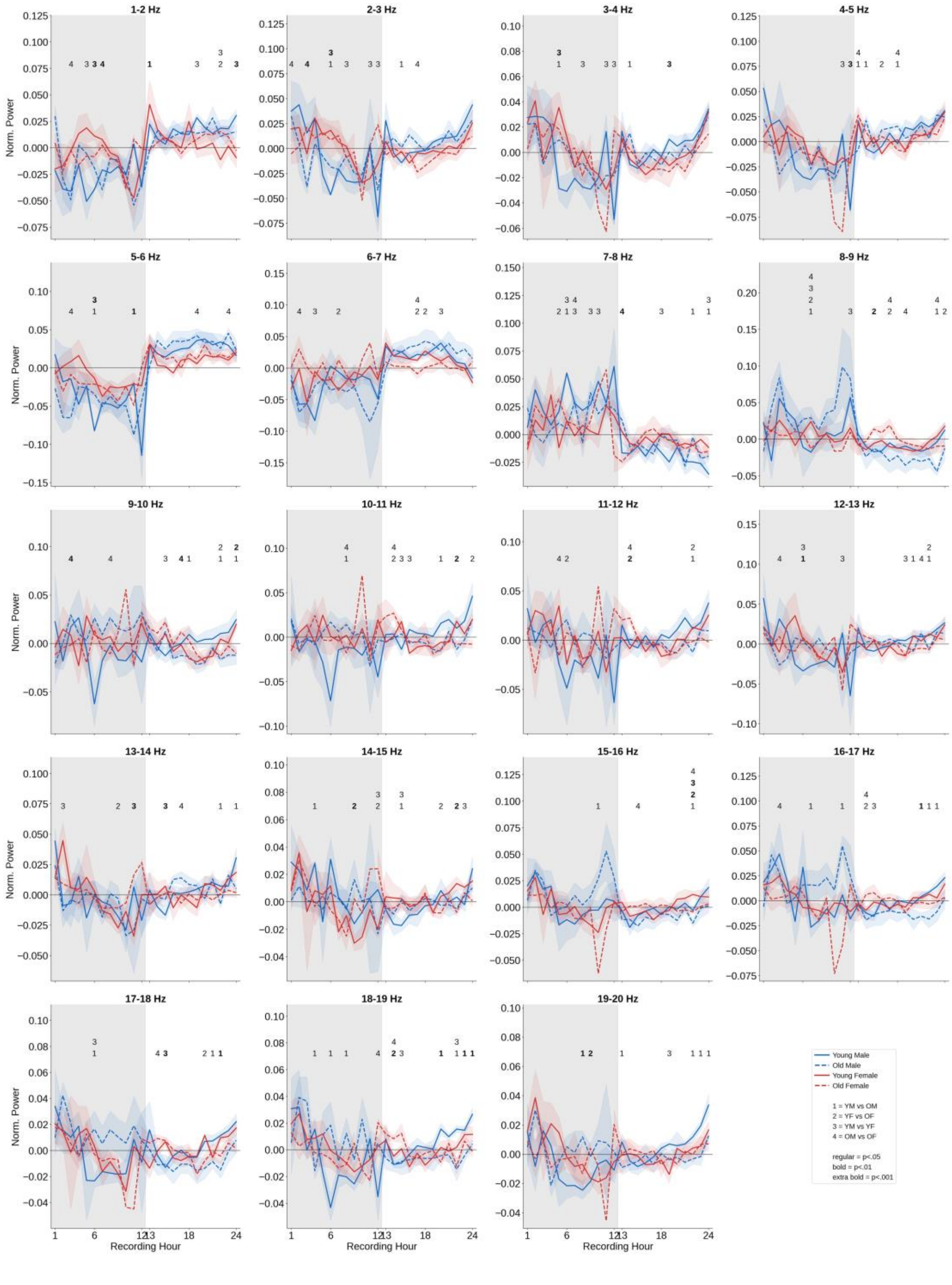


**Supplementary Figure S35. Hourly time-course of normalized EEG spectral power during REM shows broad but low-amplitude age- and sex-related effects, with higher REM data sparsity than other states.** *Values are expressed as $log_{10}$(hourly power / 24-hour mean power) for each of 19 1-Hz bands across 24 ZT hours. Lines: group means ± SEM. Group coding and annotation conventions as in Supplementary Figure S32. REM cell sparsity: 15.7%*

*of cells contained fewer than 12 clean epochs (compared with 1.1% for NREM; Section 2.12). Interpret isolated effects with caution. n = 6/group.*

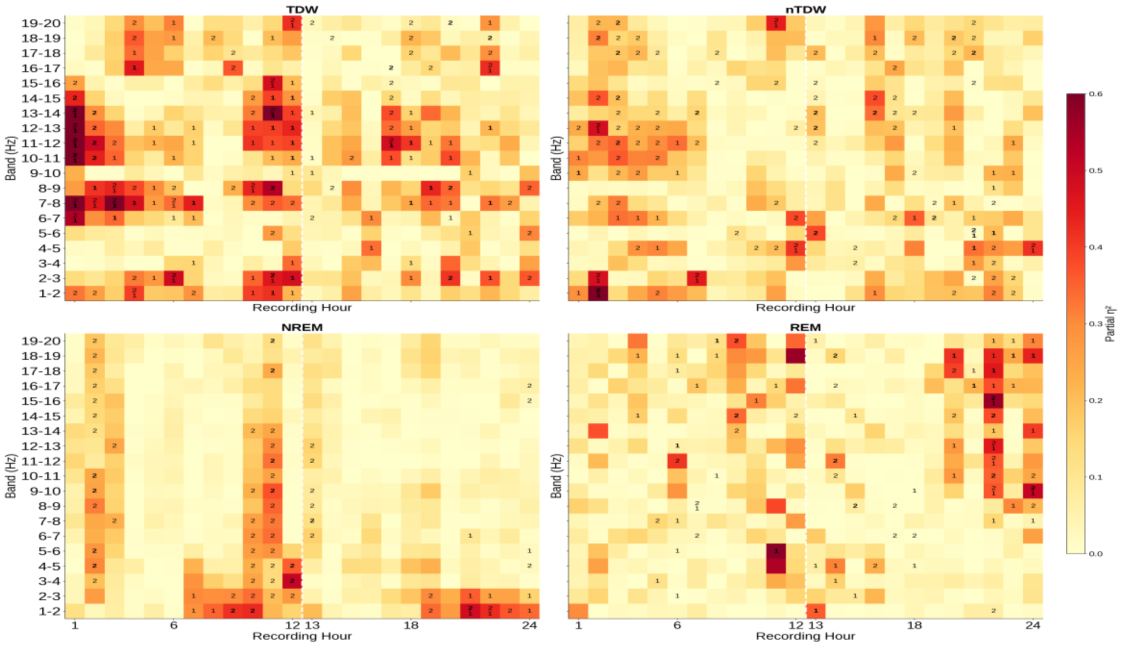


**Supplementary Figure S36. Partial-$\eta^2$ magnitudes confirm that the Age effect is concentrated in early-dark TDW sigma/high-alpha ($\eta^2$p up to 0.70) and in nTDW 1–2 Hz at ZT13 ($\eta^2$p up to 0.65).** *Heatmap of partial $\eta^2$ for the Age main effect from the 2 × 2 Age × Sex ANOVA on normalized $log_{10}$ spectral power (24-hour normalization; Section 2.11.2). Layout identical to the F-statistic heatmap in Figure 8 (4 state panels × 19 bands × 24 hours). Color scale 0–0.6; values > 0.6 are rendered at ceiling. The 20 cells with largest $\eta^2$p are listed in Supplementary Table S24. n = 6/group.*

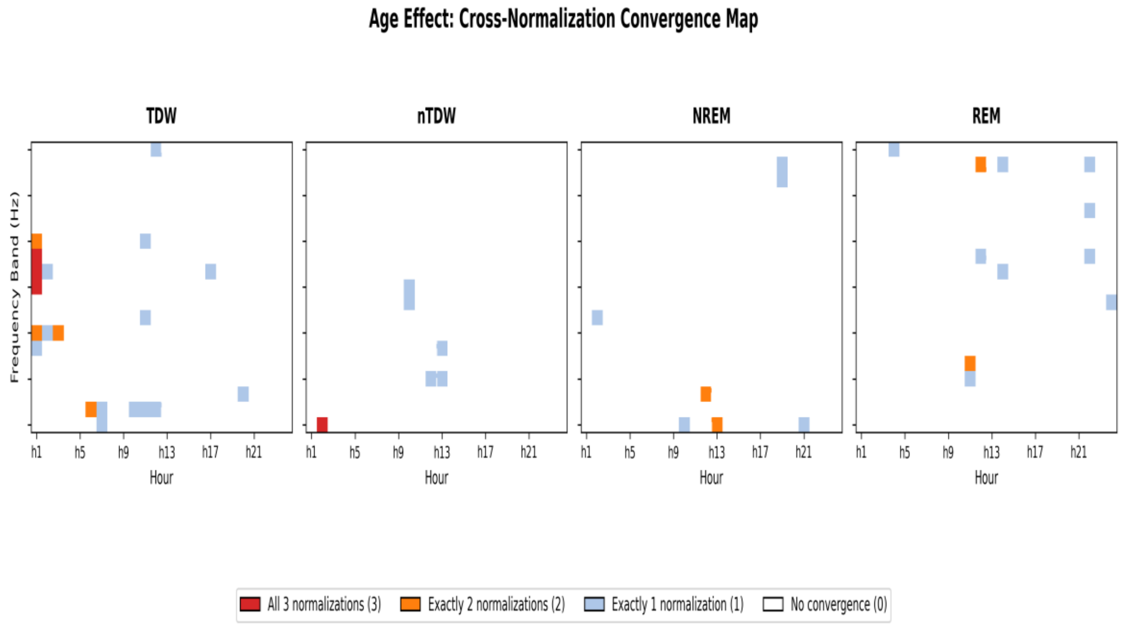


**Supplementary Figure S37. Convergence map across normalizations identifies four core age-effect loci in TDW dark phase (ZT12, 10–13 Hz) and one in nTDW dark phase (ZT13, 1–2 Hz) that survive all three reference frames.** *Each panel shows one vigilance state on a 19-band × 24-hour grid (bands on y, hours on x; ZT12 = hour 1 = dark onset). Cells color-coded by convergence status: red = in the top-20 (by Age partial $\eta^2$) under all three normalizations; orange = top-20 under two of three; light blue = top-20 under one; white = not in any top-20. Convergence counts and cell identities are listed in Supplementary Table S27. n = 6/group.*

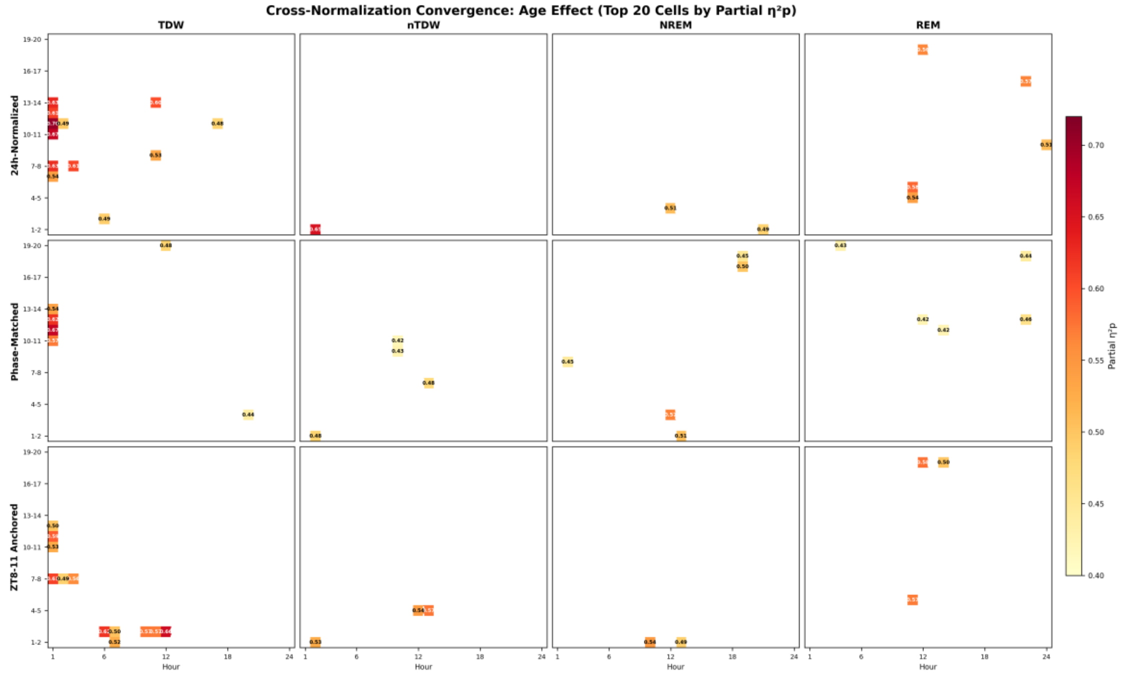


**Supplementary Figure S38. Cross-normalization comparison of the top-20 Age-effect cells; TDW 10–13 Hz at ZT12 and nTDW 1–2 Hz at ZT13 appear in all three rankings.** *Heatmap membership matrix: only cells that fall in the top 20 by partial $\eta^2$ under at least one of the three normalizations (24-h, phase-matched, ZT8–11; Section 2.11.2) are colored. Rows: normalization. Columns: vigilance state. Cells not in any top-20 are white. Convergent loci are listed in Supplementary Table S27. n = 6/group.*

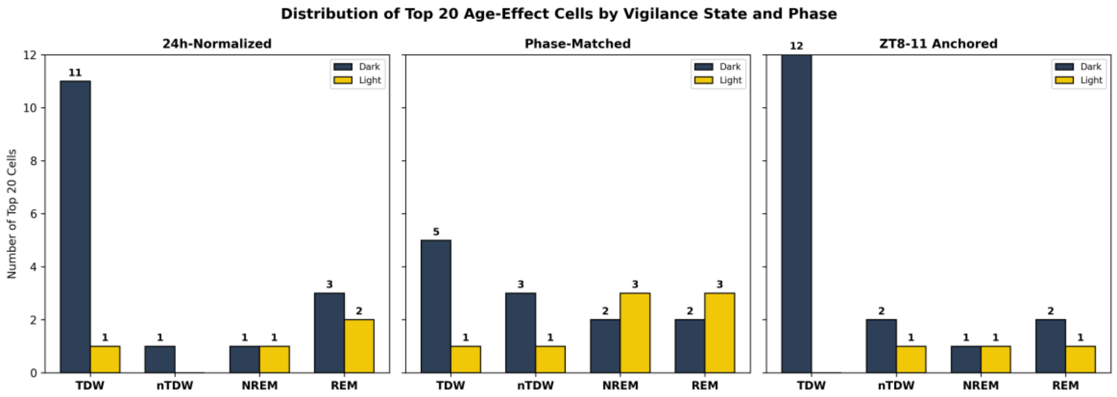


**Supplementary Figure S39. Top-20 Age-effect cells concentrate in the dark phase of wake states across all three normalizations, with REM contributing more cells under ZT8–11 anchoring.** *Stacked bar chart showing the count of top-20 Age-effect cells (by partial $\eta^2 p$) grouped by vigilance state (TDW / nTDW / NREM / REM) and circadian phase (dark / light), separately for each of the three normalizations (24-h, phase-matched, ZT8–11; Section 2.11.2). See Supplementary Table S27 for the full cell listings. n = 6/group.*

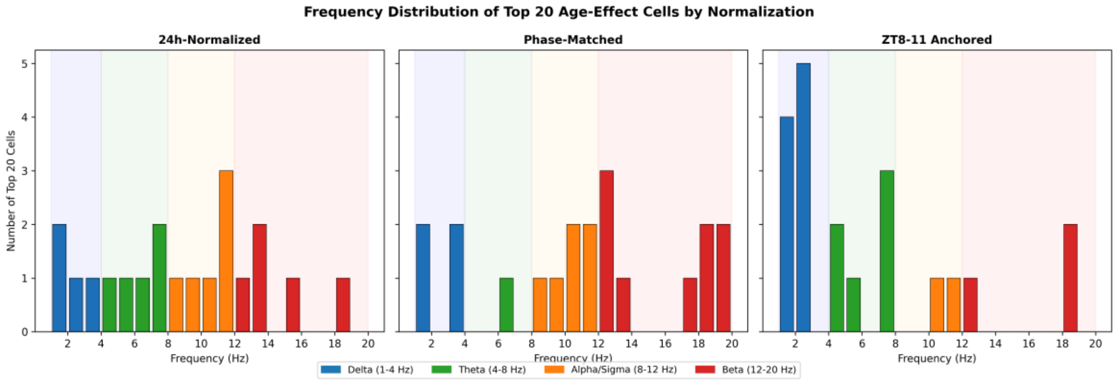


**Supplementary Figure S40. Frequency distribution of top-20 Age-effect cells across the 19 spectral bands shows that sigma/high-alpha (10–13 Hz) and low-frequency (1–3 Hz) loci dominate, regardless of normalization choice.** *Histograms of the 20 cells with largest partial* $\eta^2$ *for the Age effect, binned by their 1-Hz frequency band, shown separately for each of the three normalizations (24-h, phase-matched, ZT8–11). x-axis: 1-Hz band center (1.5–19.5 Hz); y-axis: cell count (0–20). Corresponding full cell listings in Supplementary Table S27. n = 6/group.*

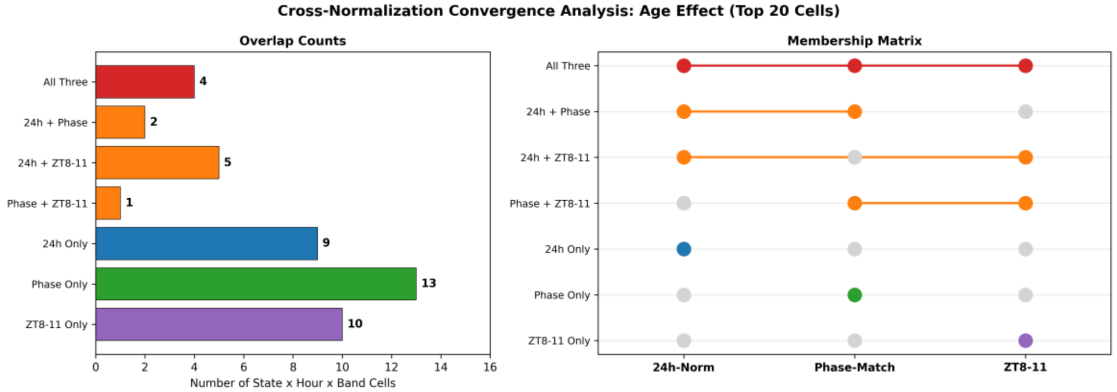


Supplementary Figure S41. Convergent age-effect loci — TDW 10–13 Hz at ZT12 and nTDW 1–2 Hz at ZT13 — are detectable across all three normalization frameworks. Cross-framework convergence of the top 20 State × Band × Hour cells ranked by partial $\eta^2$ for the Age effect under three normalizations: 24-h mean, phase-matched (dark→dark, light→light), and ZT8–11 anchored (all hours → late-rest-phase mean). Left: count of cells appearing in 1, 2, or all 3 top-20 rankings. Right: membership matrix across frameworks. Four cells converge across all three and also fall within CBPT Age × Sex clusters from Figure 7. Detailed listing: Supplementary Table S30. n = 6/group.

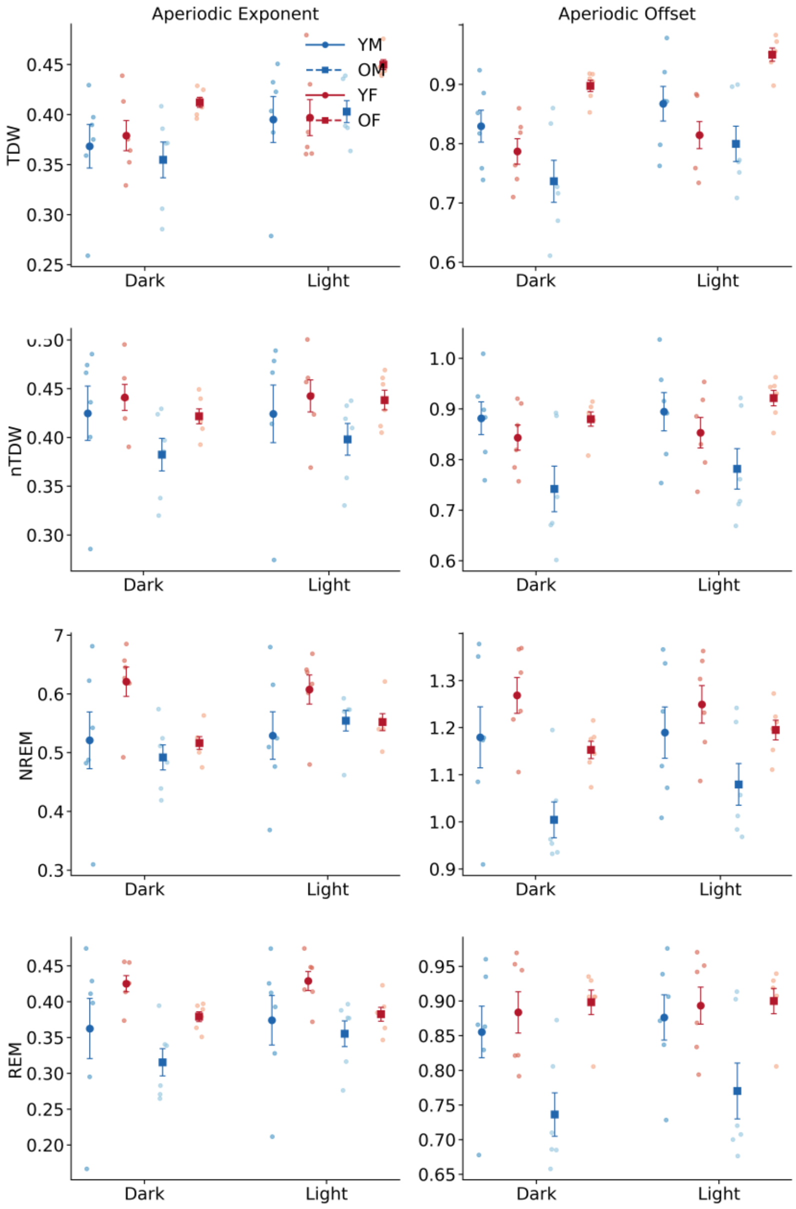

Aperiodic Exponent
Aperiodic Offset
YM
OM
YF
OF
TDW
nTDW
NREM
REM
Dark
Light

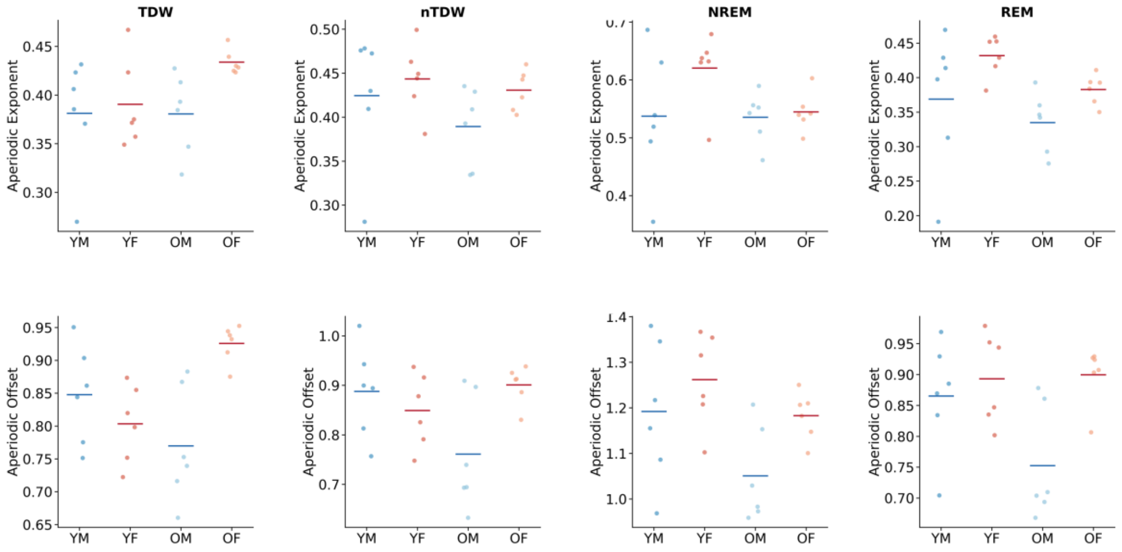


**Supplementary Figure S42. FOOOF spectral parameterization shows age-related reductions in theta center frequency across TDW, nTDW, and NREM, with robust Age × Sex interactions in aperiodic offset during wake.** FOOOF (specparam v2.0.0rc6) decomposition of absolute PSD into aperiodic and periodic components over 1–20 Hz (fixed aperiodic mode, no knee; Section 2.11.3). Mean model $R^2$ across all mouse × state × phase fits = 0.977 ± 0.019. (A) Aperiodic parameters: exponent and offset, by group × state (averaged across circadian phases). (B) Periodic theta-peak parameters: center frequency (5–9 Hz), peak power, and bandwidth, by group × state. Error bars: ±SEM. Asterisks indicate FDR-significant effects from linear models (Age, Sex, Age × Sex): *q < 0.05, **q < 0.01, ***q < 0.001. Full parameter table in Supplementary Table S29. n = 6/group.

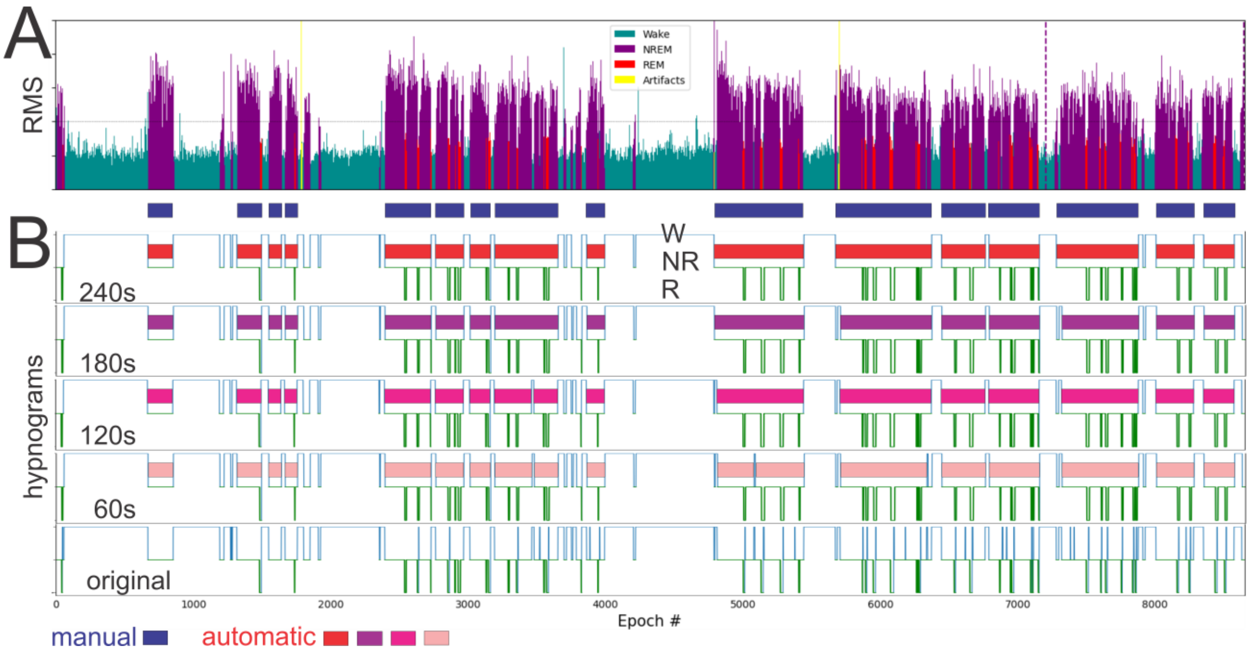

**Supplementary Figure S43. Representative example of the empirical evaluation used to select the wake-bridging threshold.** (A) Root-mean-square (RMS) of delta-band (0.5–4 Hz) EEG activity across a 24-h recording from one representative mouse, color-coded by the vigilance state scored for each epoch: wake (aquamarine), NREM (purple), REM (red), and artifact epochs (yellow). (B) Ultradian-block extraction at four wake-bridging thresholds (240, 180, 120, and 60 s) compared to the underlying hypnogram (bottom row, "original"). For each threshold, the hypnogram is shown as a wake/NREM/REM trace with REM episodes marked in green; horizontal bars above each trace represent the automated clusters (blocks) generated at that wake-bridging threshold, with colors indicating the threshold (240 s = dark red, 180 s = purple, 120 s = magenta, 60 s = salmon). The blue bars above the "W NR R" column indicate blocks identified manually from the hypnogram, which served as the reference for empirical calibration. Shorter wake-bridging thresholds (60 s) fragmented NREM–REM clusters excessively, whereas longer thresholds (240 s) merged adjacent clusters into extended blocks; the 180 s threshold (≈3 min) produced the closest match to the manual reference. This illustrative comparison, performed across mice and across wake-bridging thresholds ranging from 30 s to 10 min in 30-s increments, provided the empirical basis for selecting the 3-min threshold used throughout the analysis (see Methods §2.10).

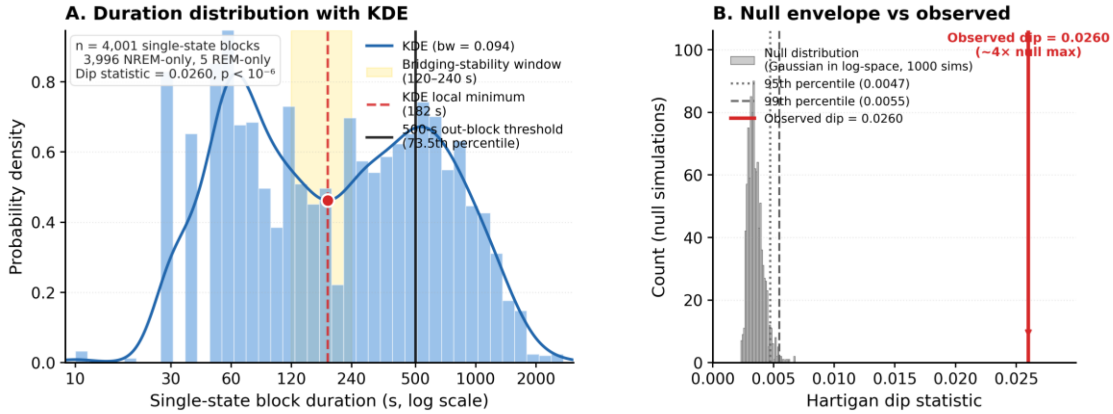


**Supplementary Figure S44. Bimodality of single-state block durations supports the 500-s out-block threshold.** Distribution of single-state block durations (NREM-only or REM-only blocks as defined in Methods §2.10; 3-min wake-bridging threshold) pooled across the full cohort of 24 mice × 14 recording days, yielding n = 4,001 blocks (3,996 NREM-only, 5 REM-only). (A) Histogram of $\log_{10}$-transformed durations (40 equal-log bins, light blue) overlaid with a Gaussian kernel density estimate (KDE; Scott's rule bandwidth = 0.094 on the $\log_{10}$ scale; dark blue curve). The KDE exhibits two clear modes — a brief-intrusion mode centered near 60 s and a consolidated single-state-block mode centered near 700 s — separated by a local density minimum at 182 s (red dashed line and marker), which falls inside the independently derived 120–240 s bridging-stability window (yellow shading; see Supplementary Figure S43). The 500-s out-block duration threshold (solid black line) sits at the 73.5th percentile of the distribution, to the right of the density minimum. Hartigan's dip test on $\log_{10}$(duration) rejects unimodality (dip statistic D =

0.0260, $p < 10^{-6}$). Inset text block summarises sample composition and the dip-test result. (B) Null envelope for the dip statistic under the unimodal null hypothesis. Light-gray histogram shows the distribution of D computed on 1,000 parametric bootstrap replicates, each drawn from a Gaussian in log-space with the same mean ($\mu = \log_{10}$ duration sample mean) and standard deviation as the observed data and matched sample size (n = 4,001). The 95th and 99th percentiles of this null distribution (0.0047, dotted; 0.0055, dashed) and the null maximum (0.0068) are all well below the observed D = 0.0260 (solid red line), which lies at approximately 4× the null maximum. Axes: Panel A x-axis, log-spaced ticks at natural second values (10, 30, 60, 120, 240, 500, 1000, 2000); Panel A y-axis, probability density; Panel B x-axis, Hartigan dip statistic on the same scale as observed and null; Panel B y-axis, count of null simulations. The large separation between observed D and the null envelope confirms that the bimodal signature in Panel A reflects structural bimodality in single-state block durations rather than KDE-kernel artifact, justifying the two-component interpretation used in Methods §2.10 to partition transient intrusions from consolidated blocks and supporting the 500-s cutoff that places the out-block threshold on the right flank of the brief-intrusion mode at the 73.5th duration percentile.

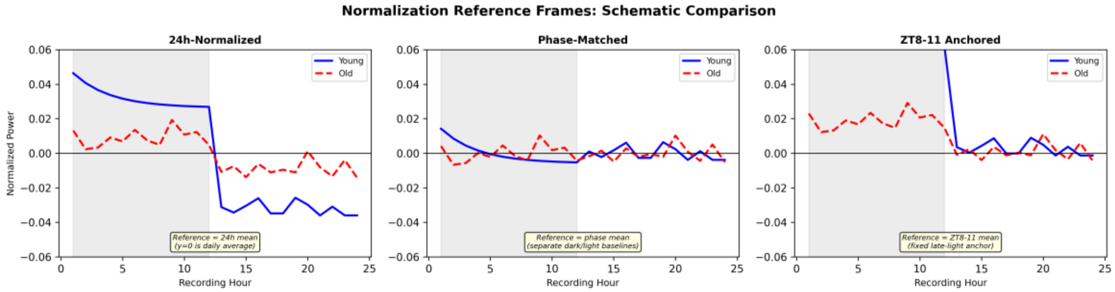


**Supplementary Figure S45. Schematic illustration of the three normalization reference frames used in the hourly spectral-distribution analysis.** *Each normalization defines a distinct zero-reference and therefore emphasizes a different component of the age- and sex-related spectral phenotype: (A) 24-hour normalization, $\log_{10}$(hourly power / 24-hour mean), isolates within-day temporal redistribution and zero-centers each mouse's profile. (B) Phase-matched normalization, dark hours referenced to dark-phase mean and light hours to light-phase mean, removes the global dark–light offset to isolate within-phase architecture. (C) ZT8–11 anchored normalization, all hours referenced to the late-rest-phase mean, preserves between-group offsets and does not impose a zero-sum constraint. Full methodological rationale in Section 2.11.2.*
