# Supplementary material for: A Hierarchical and Multiscale Framework for Characterizing Mouse Sleep–Wake Dynamics from 14-Day Continuous EEG: Validation of Age- and Sex-Dependent Remodeling
Source: Cells. 2026 Jun 13;15(12):1075. doi: 10.3390/cells15121075 (PMC13296934; doi:10.3390/cells15121075)
Supplement: Supplementary file 1 [file cells-15-01075-s001.zip › Supplementary Methods.pdf]

Supplementary Methods

Companion to the main manuscript. References cited by number refer to the main paper's bibliography.

Supplementary Methods S1. CLR-transformed compositional analysis of vigilance-state percentages

Because the four vigilance states (TDW, nTDW, NREM, REM) sum to 100% in every time bin, the state percentages are compositional and not statistically independent: increases in one state necessarily imply decreases in others. Although the primary analyses in this study modeled each state separately using linear mixed models on percentage values (Section 2.5.6, Section 2.6), this approach can in principle produce biased inference when the compositional dependency is strong. To verify that the separate-state analyses did not produce misleading conclusions, all primary vigilance-state percentage analyses were replicated using a centered log-ratio (CLR) transformation, which maps the four-state simplex onto unconstrained Euclidean space [20] and removes the sum-to-unity constraint.

For each animal × ZT hour cell, the four state percentages (p_TDW, p_nTDW, p_NREM, p_REM) were transformed as CLR(p_i) = log(p_i / G), where G is the geometric mean of the four percentages. Bins containing any zero state were imputed using the multiplicative replacement approach (replacement value = 0.5 / total_epochs_in_bin) before transformation. The four CLR-transformed values were then analyzed with the same linear mixed-effects model structures used in the main analyses: random-intercept LMMs with Age, Sex, and Hour fixed effects for circadian profile inference, and phase-level mouse-based contrasts for confirmatory testing.

Across all primary contrasts, the CLR-transformed analyses preserved the direction and approximate magnitude of every Holm-surviving age and sex contrast identified in the percentage-based analyses, with no additional significant contrasts emerging from the CLR framework. The dark-phase TDW reduction with aging was preserved in both sexes (females: CLR $\Delta = -0.91$, $p < 0.001$; males: CLR $\Delta = -0.78$, $p = 0.004$), the dark-phase NREM elevation in old females was preserved (CLR $\Delta = +0.42$, $p = 0.013$), and the light-phase REM reduction in old females was preserved (CLR $\Delta = -0.31$, $p = 0.007$). These results confirm that the principal findings reported in Section 3.1 are not artifacts of the compositional constraint.

Supplementary Methods S2. Threshold rationale and sensitivity analyses for ultradian-block identification

Three thresholds are required to identify ultradian sleep blocks and out-block episodes in the analysis pipeline: the wake-bridging duration (3 min), the out-block minimum block length (500 s), and the out-

block isolation criterion (180 s). Each was selected a priori during protocol development on distinct empirical grounds. This section documents the rationale for each choice and the sensitivity analyses confirming robustness.

Wake-bridging threshold (3 min)

The 3-min bridging threshold was determined by comparison to manual reference scoring. Clear clusters of repeating NREM–REM cycles (typically 1–15 cycles) separated by brief wake periods were first identified manually from hypnograms to serve as ground truth. Automated blocks were then generated across wake-duration thresholds ranging from 30 s to 10 min in 30-s increments (Supplementary Figure S43). Shorter thresholds fragmented clusters excessively, whereas longer thresholds merged adjacent clusters into artificially prolonged blocks. The 3-min value produced the closest match to manual clusters, and thresholds between 120 and 240 s yielded comparable circadian, age-, and sex-dependent patterns, defining the stability window around the selected value. This range is also consistent with human sleep studies in which brief 2–3 min awakenings commonly occur between sleep cycles in young adults and lengthen with aging to 5–10 min, at which point they increasingly disrupt sleep continuity. By analogy, wake episodes ≤3 min in mice were considered part of a consolidated ultradian block, whereas longer episodes were treated as block-terminating events.

Out-block thresholds (500 s minimum length, 180 s isolation)

The 500-s and 180-s thresholds for out-block classification do not have direct literature precedents and were set by qualitative inspection of the block-duration distribution as empirically reasonable values separating brief single-state intrusions from longer aborted sleep cycles. Formal analysis of the single-state block-duration distribution (Supplementary Figure S44) supported this choice. Hartigan's dip test on log-transformed durations rejected unimodality (dip statistic = 0.026, $p < 10^{-6}$), and kernel density estimation identified a local density minimum at ~182 s, within the independently-derived 120–240 s bridging stability window. This indicates structural bimodality between a brief-intrusion mode and a longer single-state-block mode, with the 500-s threshold falling to the right of this minimum at the 73.5th percentile of the single-state block population.

Sensitivity sweep across alternative threshold settings

To verify robustness to threshold choices, a one-at-a-time sensitivity sweep was conducted across eight settings covering plausible alternative values for all three parameters (Supplementary Table S30). The Age rate ratio for out-block count was preserved in direction (Old > Young) at every setting and remained significant at $p < 0.05$ in six of eight, with rate ratios spanning 1.23–1.43. Only one setting (out-block minimum length = 300 s) attenuated below nominal significance (RR = 1.23, $p = 0.138$), and one (bridge = 5 min) was near-significant ($p = 0.073$). The primary Age effect on out-block count is therefore robust to plausible variations in all three threshold parameters.

Supplementary Methods S3. Statistical analysis of 24-hour vigilance-state profiles: full specifications

This section provides the full specifications for the three-tier inferential framework described in main-text Section 2.6. The approach applies within the constraints of n = 6 per group, with phase-level mouse-based contrasts as the only formally confirmatory tier; the omnibus LMM tests global profile shape and cannot localize where differences occur, and the hourly EMM contrasts and cosinor summaries are descriptive and hypothesis-generating. The rationale for this tiered approach is that each tier addresses a different aspect of the data: the phase-level contrasts provide maximum statistical independence (insensitive to within-mouse hourly serial correlation), the omnibus LMMs test global profile shape, and the hourly contrasts localize effects temporally but with reduced inferential weight.

Tier 2: Global circadian profile (linear mixed models)

Gaussian linear mixed models (LMMs) were fitted for each vigilance state with a random intercept for each mouse (Mouse_ID) and a 24-level categorical factor of time (Hour) as a fixed effect. Interactions were evaluated by sequential top-down maximum-likelihood (ML) likelihood-ratio tests (LRT): the three-way Age × Sex × Hour was tested first; if non-significant, the two-way interactions were tested. For borderline LRT results (asymptotic p between 0.01 and 0.10), an empirical p-value was additionally computed from a parametric bootstrap (500 resamples under the reduced model) to guard against the anti-conservative bias of the asymptotic χ^2 approximation at the present sample size. Main effects were not interpreted when their Hour interaction was significant, because the interaction indicates that the effect varies across the circadian cycle.

A random-intercept-only random-effects structure was used because the sample size of n = 6 per group is insufficient to reliably estimate random slopes for the 24-level Hour factor. The known consequence is that between-mouse differences in circadian shape are absorbed into the residual variance rather than partitioned into a random Hour-by-Mouse component, which can narrow standard errors for Hour-containing interaction terms (Age × Sex × Hour, Age × Hour, Sex × Hour) and potentially inflate LRT statistics for those interactions. Relatedly, the 24-level Hour factor consumes many numerator degrees of freedom in three-way tests relative to the effective sample size (n = 24 mice), making the asymptotic χ^2 distribution an imperfect reference; the parametric bootstrap described above partially mitigates this by computing empirical null distributions for borderline results. These considerations further reinforce that the Tier 2 LMM is not treated as formally confirmatory: confirmatory inference rests on the Tier 1 phase-level mouse-based contrasts, which operate on phase-averaged mouse-level means and are invariant to hourly parameterization. The Tier 2 LMM is reported to characterize global profile shape, and its Hour-containing interaction statistics should be interpreted as descriptive rather than as independent confirmatory evidence.

To verify that these Hour-containing interactions are not artifacts of the 24-level parameterization, the Tier 2 LMMs were refit using two-harmonic (24-h + 12-h) and three-harmonic (24-h + 12-h + 8-h) cosinor bases for the time axis as alternative low-dimensional representations, with inference performed via OLS with cluster-robust standard errors (Mouse as clustering variable [61]) to circumvent small-sample convergence difficulties of the mixed-model likelihood at full three-way parameterization. Results are reported in Supplementary Table S1: Age × Time interactions are robustly significant across all four vigilance states under every parameterization ($p < 10^{-4}$ throughout), supporting the conclusion that the age-related reshaping of the circadian profile is not an artifact of the categorical hourly specification. Sex × Time interactions partially attenuate under harmonic parameterization — from $p < 0.001$ (hourly) to $p = 0.007–0.079$ (two-harmonic) and $p = 0.009–0.027$ (three-harmonic) depending on state — consistent with the degrees-of-freedom concern for this secondary effect, and they should accordingly be interpreted with the same descriptive framing as the hourly model. The three-way Age × Sex × Time interaction remains non-significant under every parameterization ($p > 0.19$ under harmonic), confirming the finding that age's circadian reshaping does not depend on sex in this sample.

Because the LMM operates on 14-day-averaged hourly profiles rather than on day-level repeated observations, it is not a longitudinal day-level model; the effective inferential unit remains the 24 mice (n = 6 per group), and the LMM tests profile-shape differences across groups rather than day-to-day dynamics.

Tier 3: Exploratory hourly localization

Exploratory localization employed cascade-gated hourly estimated marginal mean (EMM) contrasts, computed only where the corresponding phase-level contrast (Tier 1) was significant. This gating procedure ensures that hourly contrasts are not performed in the absence of phase-level evidence, thereby limiting false-positive propagation. Hourly p-values are Holm-corrected within each 12-hour family and interpreted as descriptive localizations of when within the phase the effect is most pronounced, rather than as independent confirmatory tests, given the residual temporal autocorrelation at the hourly level.

Diagnostics, sensitivity, and cosinor characterization

Model diagnostics included Q–Q plots of residuals, residuals-versus-fitted plots, per-mouse autocorrelation function (ACF) analysis, convergence diagnostics (BLUPs, multi-optimizer checks, variance components, eigenvalue spectrum), and Nakagawa's marginal and conditional R^2. Autocorrelation diagnostics assessed lag-1 ACF and Bartlett's effective N per mouse to quantify the degree of temporal autocorrelation and evaluate the compound symmetry assumption. Two-harmonic cosinor models (24-h fundamental plus 12-h harmonic) were fitted to each group's 24-hour profile for descriptive characterization of circadian parameters (mesor, amplitude, acrophase). A two-harmonic

model was chosen because single-harmonic fits poorly captured the bimodal structure of TDW profiles. Sensitivity analyses included exclusion of 5 mice with within-mouse CV > 0.80, and CLR-transformed replication of all primary contrasts (Supplementary Methods S1).

Supplementary Methods S4. Statistical analysis of NREM temporal architecture: full phase specifications

This section provides the full phase-by-phase specifications for the hierarchical analytical framework described in main-text Section 2.7. All analyses are organized into three prespecified inferential tiers with independent multiplicity control: Tier 1 (primary, confirmatory) = TDW fraction at ZT16 in a factorial Age × Sex framework; Tier 2 (secondary, Holm–Bonferroni corrected) = the five primary N-shape landmarks, including TDW fraction at the data-driven trough as a descriptive characterization of where within the ZT20–ZT2 window the age-related TDW suppression is maximal; Tier 3 (exploratory, nominal) = all Phase 1 GAM profiles, Phase 2 binned metrics, exploratory and descriptive landmarks, and per-component wake analyses.

Phase 1: Whole-profile functional contrast analysis

Group-specific generalized additive models (GAMs) were fitted using pyGAM with thin-plate regression splines (20 splines, cubic order) to each group's animal-level data (6 animals × 96 bins = 576 observations per group). GAMs were used for curve estimation; statistical inference relied solely on bootstrap comparisons at the animal level. Bootstrap difference smooths (1,000 iterations) were computed by resampling animals with replacement within each group (N = 6), fitting GAMs to each bootstrap sample, and computing the difference in predicted curves. Contiguous regions where the 95% bootstrap confidence interval excluded zero ("significant runs") were reported with their ZT boundaries and durations. Six contrasts were evaluated: four within-factor comparisons and two collapsed marginal contrasts (not main effects from an integrated factorial model). Leave-one-out jackknife validation confirmed that no single animal drove the divergence patterns. The Phase 1 analysis is descriptive-localizing (Tier 3) and does not carry confirmatory inferential weight.

Phase 2: Ultradian oscillation landscape

All prominent NREM peaks and troughs were detected in each animal's smoothed profile using scipy.signal.find_peaks with the following criteria: minimum prominence ≥10% of the local range (defined as the max–min difference within ±3 bins of the candidate peak), minimum inter-peak distance ≥4 bins (1 hour), and minimum peak width ≥2 bins (30 min). For each detected peak, ZT timing, NREM amplitude, prominence, and interval since the preceding peak were recorded. For each trough, ZT timing, NREM depth, and state composition (TDW fraction and absolute TDW and nTDW percentages) were recorded. Detected events were binned into four circadian phases (early dark ZT12–ZT18, late dark

ZT18–ZT0, early light ZT0–ZT6, late light ZT6–ZT12), and per-animal summaries within each bin were computed. Group differences in binned metrics were tested using Mann–Whitney U tests as Tier 3 exploratory within-phase comparisons.

Phase 3: N-shape landmark extraction

Within the predefined ZT16–ZT6 window, Peak 1 (late-dark NREM maximum), the trough (pre-lights-on minimum, constrained to ZT20–ZT2), and Peak 2 (early-light NREM surge) were extracted from each animal's smoothed NREM profile. Five primary landmarks were computed per animal (Tier 2, Holm-corrected): Peak 1 ZT, Peak 1 amplitude, time to Peak 1, FWHM of Peak 1, and TDW fraction at the data-driven trough — the last presented as a descriptive characterization of where within the ZT20–ZT2 window the age-related TDW suppression reaches its maximum, complementing the Tier 1 primary endpoint at the fixed pre-specified ZT16 time point. Five exploratory landmarks were also computed: trough ZT, Peak 2 amplitude, Peak 2/Peak 1 amplitude ratio, asymmetry index, and NREM–TDW correlation. Because the trough search window (ZT20–ZT2) spans midnight, trough ZT values were handled with circular statistics: group summaries are reported as circular mean ± circular SD (scipy.stats.circmean/circstd on values converted to radians on the 24-hour clock), and hypothesis tests for trough ZT were performed on the linear representation "hours after ZT20" (values in [0, 6] h). Primary landmarks (Tier 2) were corrected for multiple comparisons using the Holm–Bonferroni method across 10 tests (5 landmarks × 2 factors); exploratory landmarks (Tier 3) were evaluated with nominal p-values.

Phase 4: State composition at transitions

To characterize the vigilance-state composition at each N-shape landmark, the raw (unsmoothed) state percentages were sampled at the bin closest to each animal's detected Peak 1, trough, and Peak 2, as well as at five fixed reference points spanning the N-shape (ZT16, ZT20, ZT23.5, ZT0.5, ZT3). For each timepoint, NREM, REM, TDW, and nTDW percentages were recorded along with the derived TDW fraction. Because wake microcomposition is compositional (TDW and nTDW fractions of total wake are constrained), the primary compositional analysis used a binomial generalized linear model on TDW counts out of total wake counts (90 ten-second epochs per 15-min bin), with logit link and Age × Sex fixed effects. Per-component tests on absolute TDW and nTDW percentages were run as Tier 3 supporting analyses.

Phase 5: Sensitivity and robustness

Parameter sensitivity was assessed across nine combinations of Savitzky–Golay smoothing window (3, 5, 7 bins) and peak prominence threshold (8%, 10%, 12%), with stability quantified by intraclass correlation coefficient (ICC(2,1)) of animal-level landmark estimates across variants. Because the Tier 1 primary endpoint is sampled at a fixed ZT16 bin rather than at a data-driven landmark, it does not depend on peak-detection smoothing or prominence thresholds; the sensitivity analysis therefore applies only to Tier

2 landmark-based metrics. Additional robustness checks included: (a) Day 0 exclusion; (b) leave-one-out jackknife on the Phase 1 GAM; (c) day-slope analysis testing for progressive drift in ZT16 TDW fraction across the 14 recording days; (d) day-level validation of the primary endpoint: the ZT16 TDW fraction was re-extracted independently for each of the 14 recording days and each of 24 animals (320 animal-day observations after excluding 16 animal-days with zero wake epochs in the ZT16 bin) and analyzed with a mixed-effects model (ZT16 TDW fraction ~ Age × Sex + (1 | Mouse)) to confirm that the primary effect is not an artifact of 14-day averaging, with the day coefficient estimated in a separate additive-Day model; an equivalent day-level validation was also performed at the data-driven trough (336 animal-day observations) as a Tier 2 supporting analysis; and (e) two-harmonic cosinor analysis of ZT16–ZT22 NREM segment as an exploratory measure of dark-phase bimodality (H2/H1 amplitude ratio).

Supplementary Methods S5. Statistical analysis of episode architecture and bout-length distributions: full specifications

This section provides full specifications for the analyses described in main-text Section 2.8. Episode architecture was quantified by two complementary metrics — mean episode duration and episode count — computed separately for each state. Phase-level duration and episode count are the primary architecture outcomes; hourly LMM profiles and cascade-gated localizations serve as secondary temporal-localization analyses.

Tier-1 confirmatory analyses: phase-level architecture

Each mouse contributed a single dark-phase and light-phase mean; twelve between-group contrasts per state were Holm–Bonferroni corrected. The Holm-corrected family per state comprised the four pairwise group contrasts within dark phase (Age within sex, Sex within age) plus the four within-group dark-vs-light paired contrasts and the four light-phase between-group contrasts, with phase-level analyses reported as the primary inferential layer. Effect sizes used Hedges' g with bootstrap 95% confidence intervals (10,000 resamples). Tier 2 (secondary) used hourly cascade-gated EMM contrasts only where the corresponding phase-level contrast was significant, with Holm correction within each 12-hour family. Tier 3 (exploratory) examined collapsed pooled contrasts and cosinor-summarized circadian profiles.

Bout-length distribution analysis

Bout-length distributions were analyzed under a complementary three-tier framework.

Tier 1: Survival analysis (Cox proportional hazards)

Cox proportional hazards models were fitted with cluster-robust standard errors (Mouse_ID), including Age, Sex, Age × Sex, and cosinor harmonics ($\cos[2\pi \cdot ZT/24]$, $\sin[2\pi \cdot ZT/24]$). HR > 1 indicates a higher state-exit rate (shorter bouts). The last episode of each 24-hour recording was treated as right-censored.

The proportional-hazards assumption was tested via Schoenfeld residuals; where violated, accelerated failure time (AFT) models with Weibull, log-normal, and log-logistic baseline distributions were fitted as complementary specifications. Cox and AFT specifications converged directionally for all primary contrasts; quantitative results presented in main text follow the Cox framework.

Tier 2: Summary metrics and bi-exponential mixture decomposition

Six metrics were extracted per mouse × phase × state: mean and median duration, short-bout fraction (<2 min), long-bout fraction (>10 min for TDW/nTDW/NREM, >3 min for REM), bout rate (episodes/h of state time), CV of durations. Day-averaged values were analyzed with factorial Age × Sex ANOVA. Bi-exponential mixture models (two-component exponential mixtures) were fit independently per mouse to dark-phase and light-phase TDW bout-length distributions via maximum likelihood estimation. Model parameters (proportion of short component, time constants of short and long components) were extracted per mouse and analyzed with factorial Age × Sex ANOVA. BIC favored 2-component fits for TDW in 79% of mice in the dark phase and 100% of mice in the light phase, and 1-component fits for NREM and REM in all mice. Where mixture decomposition was appropriate, both short-bout and long-bout components were tested separately for age and sex effects.

Tier 3: Bout-length distributions across circadian segments

Episode durations were binned at 1-min resolution across 3-h circadian segments. Two-way ANOVA annotations on log-transformed durations (Age × Sex) were applied at the segment level for exploratory characterization; these were treated as descriptive rather than confirmatory.

Supplementary Methods S6. Statistical analysis of EEG spectral data: full tiered architecture

This section provides full specifications for the spectral-analysis framework described in main-text Section 2.11. The analysis was organized hierarchically across three tiers. Tier 1 (primary whole-spectrum inference) is based on cluster-based permutation testing (CBPT) of absolute PSD, providing the strongest inferential foundation and defining what truly differs in the frequency domain between Age × Sex groups. Tier 2 (primary temporal-allocation analysis) uses hourly normalized spectral profiles under three complementary normalization frameworks to determine when across the 24-hour cycle those differences are expressed. Tier 3 (secondary interpretive analyses) employs relative PSD, scalar band-power summaries, and spectral parameterization (FOOOF/specparam) to evaluate whether observed effects reflect proportional redistribution, broadband shifts, periodic peak changes, or a mixture of these. Frequency-resolved bin-wise models were used as a secondary estimation layer within Tier 1 to assess concordance with CBPT and estimate pointwise effect profiles.

Frequency-resolved bin-wise models

To estimate pointwise effect profiles, linear models identical in fixed-effect structure to the CBPT base model were fitted independently at each of the 191 frequency bins within each state × phase combination, yielding 4,584 tests per spectral representation (191 bins × 4 states × 2 phases × 3 effects). Raw p-values were FDR-adjusted (Benjamini–Hochberg, $q < 0.05$) within each state × phase × effect family. These bin-wise models were used only to estimate beta coefficients, visualize effect topography, and quantify concordance with CBPT; they were not used as the basis for primary significance claims. Omnibus likelihood-ratio tests compared models with Age × Frequency and Sex × Frequency interaction terms to reduced models containing only main effects; significant interaction terms were interpreted as evidence of frequency-dependent spectral structure justifying the frequency-resolved analyses.

Effect-size quantification

Frequency-resolved Cohen's d was computed at each bin for the Age contrast (old vs. young, pooled across sex) and the Sex contrast (female vs. male, pooled across age). A crossed group contrast (old female vs. young male) was plotted to visualize maximum phenotypic separation; because the crossed contrast conflates main effects with their interaction, it was used descriptively only.

Three normalization approaches for hourly spectral analysis

Three complementary normalizations were used, each addressing a distinct biological question (Supplementary Figure S45).

24-hour normalization (primary). Each hourly value was expressed as $\log_{10}$(hourly power / 24-hour mean power) within mouse, state, and band. This produces a zero-centered profile that isolates temporal redistribution across the full day.

Phase-matched normalization. Dark-phase hours were referenced to the dark-phase mean and light-phase hours to the light-phase mean, independently within mouse, state, and band. This removes the global dark–light offset and isolates within-phase temporal architecture.

ZT8–11 anchored normalization. All hours were referenced to the mean power during ZT8–11, a late-rest-phase reference window chosen as a stable physiological anchor. Unlike the other two transformations, this normalization preserves between-group offsets and does not impose a zero-sum constraint.

Hourly spectral statistics

At each State × Band × Hour cell, a 2×2 between-subjects ANOVA tested Age, Sex, and Age × Sex effects on normalized $\log_{10}$ spectral power (1,824 omnibus tests in total). FDR correction was applied separately within each vigilance state and ANOVA term (456 tests per state × effect family). Four planned pairwise Welch's t-tests (YM vs. OM, YF vs. OF, YM vs. YF, OM vs. OF) were performed at each cell as exploratory annotation. Cross-normalization overlap of top $\eta^2 p$ cells was used as a descriptive robustness layer, not as a formal inferential test.

Tier 3 secondary analyses: relative PSD, scalar band-power, and FOOOF

Relative PSD was computed as the proportion of total 1–20 Hz power contributed by each frequency bin and analyzed with the same bin-wise linear-model framework used for absolute PSD; it was interpreted as proportional redistribution rather than direct evidence of absolute gain or loss in oscillatory power. Six scalar features were extracted from the mouse-level spectra: NREM delta power (1–4 Hz, log_{10}), NREM sigma power (10–15 Hz, log_{10}), TDW theta power (5–9 Hz, log_{10}), REM theta power (5–9 Hz, log_{10}), TDW theta-peak centroid frequency (power-weighted mean frequency within 5–9 Hz), and REM theta-peak centroid frequency (defined identically within REM). Each feature was analyzed separately for dark and light phases using linear models with Age, Sex, and Age × Sex terms, with FDR correction across the resulting 36 tests.

Spectral parameterization was performed with specparam (FOOOF) v2.0.0rc6 over the 1–20 Hz range to separate aperiodic and periodic spectral components. A fixed aperiodic mode (no knee) was used. For each mouse × state × phase spectrum, the aperiodic exponent and offset were extracted, and the dominant theta peak within 5–9 Hz was characterized by center frequency (CF), peak power (PW), and bandwidth (BW) when reliably detected. Aperiodic and periodic parameters were then analyzed with linear models containing Age, Sex, and Age × Sex terms. Benjamini–Hochberg correction was applied separately to the aperiodic and periodic families of tests.
