# Supplementary material for: A Hierarchical and Multiscale Framework for Characterizing Mouse Sleep–Wake Dynamics from 14-Day Continuous EEG: Validation of Age- and Sex-Dependent Remodeling
Source: Cells. 2026 Jun 13;15(12):1075. doi: 10.3390/cells15121075 (PMC13296934; doi:10.3390/cells15121075)
Supplement: Supplementary file 1 [file cells-15-01075-s001.zip › Supplementary Note.pdf]

# Supplementary Note S1

## Dataset validation, variance structure, and design implications

*Companion to the main manuscript. References cited by number refer to the main paper's bibliography.*

## Overview

This Supplementary Note reports the dataset-validation, variance-decomposition, and study-design analyses that accompanied the principal biological findings of the main manuscript. It serves three purposes: to confirm that the 14-day longitudinal sleep–wake dataset is statistically adequate for the inferential claims advanced in the main text; to quantify the relative contributions of between-animal and within-animal variability to inferential precision; and to provide practical sample-size and recording-duration guidance for future translational sleep–aging studies. The analyses are presented as design guidance and validation evidence, not as primary biological findings.

## S1.1 Methods

### S1.1.1 Variance decomposition

Variance decomposition used random-intercept linear mixed models fit separately for each Tier-1 metric, 6-hour circadian segment, and Age × Sex group: $Y_{ij} = \mu + \beta \cdot Day_j + u_i + \varepsilon_{ij}$, with $u_i \sim N(0, \sigma^2_{between})$ and $\varepsilon_{ij} \sim N(0, \sigma^2_{within})$. Day was included as a fixed effect to prevent residual drift from inflating within-animal variance. The intraclass correlation coefficient was computed as $ICC = \sigma^2_{between} / (\sigma^2_{between} + \sigma^2_{within})$, with 95% confidence intervals derived via the F-distribution method [62]. Compound symmetry was adopted as a parsimonious working covariance structure, supported by the lag-1 autocorrelation diagnostics summarized in S1.2.1. Five Tier-1 metrics were pre-specified at the study level: NREM percentage, REM percentage, NREM episode duration, the log-transformed fragmentation index (log(1 + brief awakenings ≤ 30 s per hour of total sleep)), and NREM delta power. Four are analyzed here; NREM delta power requires spectral decomposition and is reported in Section 3.6 of the main paper.

### S1.1.2 Stability and effect-size estimation

Stability was assessed by block bootstrap at three fixed block sizes ($b$ = 2, 3, 4 days), with 500 bootstrap samples drawn for each target recording duration ($k$ = 2 to 13 days). Convergence was evaluated against metric-specific absolute-precision thresholds set at approximately half the typical age-related difference (NREM% ≤ 3 percentage points, REM% ≤ 0.5 percentage points, NREM episode duration ≤ 10 s, log-fragmentation ≤ 0.15 log units). Thresholds were set on the basis of biological effect magnitudes rather

than as arbitrary fractions. Effect-size estimation used Cohen's d derived from per-animal 14-day means, with pooled standard deviations computed across the full Age comparison (n = 12 Young versus n = 12 Old).

#### S1.1.3 Power and allocation

Power calculations are reported as prospective design guidance, not as retrospective evidence grading. Power was computed analytically for main effects of Age and Sex using the noncentral t-distribution framework with group-specific variance components and Welch's degrees of freedom. The Age × Sex interaction was bounded analytically: minimum detectable $d \approx 1.5$–$2.0$ at 80% power with $n = 6$ per cell. Optimal allocation was derived by computing iso-precision contours in the (n_animals, n_days) plane using the relation $SE = \sqrt{(\sigma^2\_between/n + \sigma^2\_within/(n \times k))}$, with computations propagated through the upper and lower bounds of the ICC 95% confidence interval. Marginal SE reduction from one additional animal versus one additional recording day was evaluated at three baseline configurations. Procedural details for data quality assessment, drift detection, and autocorrelation testing are reported in Supplementary Methods S1 (main supplementary materials).

## S1.2 Results

#### S1.2.1 Data quality and temporal structure

The complete dataset comprised 2,903,040 epochs (24 mice × 14 recording days × 8,640 epochs per day) distributed evenly across the four Age × Sex groups. After artifact rejection, 98.9% of epochs remained for analysis (per-group artifact burden 1.0–1.4%, no group showing outlier rejection rates). Sparse-bin occurrence was concentrated primarily in REM sleep (10.4% of REM bins below the ≥ 12 clean-epoch threshold versus 0–1.7% for other states). No metric × segment combination met the pre-specified criterion for a consistent acclimation effect (directional shift in the same direction across ≥ 3 of 4 groups); only 5 of 144 contrasts reached nominal $p < 0.05$, without consistent metric- or group-specific patterns. Secular drift was minimal: grand-mean day-wise slopes for NREM% and REM% were −0.047 and −0.012 units per day, respectively. Lag-1 autocorrelation was not systematic for NREM%, REM%, or NREM episode duration (all $p > 0.27$); only the log-fragmentation index showed a small but detectable negative lag-1 correlation ($r = -0.069$, $p = 0.009$), too small to meaningfully distort the variance-partitioning results. All 14 recording days were retained for subsequent analyses.

#### S1.2.2 Variance decomposition

Variance reliability was strongly metric-dependent rather than uniform across the sleep–wake phenotype (Table S31). NREM percentage yielded the highest mean ICC (0.47), indicating that a substantial fraction of its total variance resided at the between-animal level. REM percentage (mean ICC 0.32) and NREM episode duration (mean ICC 0.36) occupied an intermediate range. The log-transformed fragmentation

index showed consistently low ICCs (mean 0.20), reflecting dominance of within-animal variance. Group-level ICCs were also asymmetric: old mice showed higher mean ICCs than young mice (OM 0.45, OF 0.39 versus YM 0.28, YF 0.22), driven by lower within-animal variance in aged animals. Young females exhibited the highest within-animal variance across several metrics, making this subgroup the least statistically efficient for convergence of individual-level estimates. Possible reproductive-cycle contributions cannot be evaluated in this dataset and remain hypothetical. Heteroscedasticity across groups was substantial ($\sigma^2$_within differed by up to 4.5-fold for the same metric × segment combination), and group-specific variance components were carried forward into all power and allocation computations.

### S1.2.3 Effect-size landscape and prospective power

The effect-size landscape was highly segment-specific, reinforcing the broader logic of the main paper that circadian phase is a major determinant of effect detectability. Large age effects clustered in selected segments: the largest observed Cohen's d was 2.36 for NREM% in the Dark 1 segment (ZT12–18), with additional large age effects for the log-fragmentation index in Dark 1 (d = 1.50) and REM% across segments (d = 1.2–1.4). NREM episode duration showed near-null age effects across all four segments. The absence of a uniformly large whole-day effect therefore does not imply biological absence; it can reflect aggregation of phase-restricted differences with intervals of weak group separation. Under the present variance structure, only the very largest effects are well powered at n = 6 per group with 14 recording days. The Age effect on Dark 1 NREM% achieves approximately 95% power; other large effects (d = 1.2–1.5) reach only 0.45–0.56 power. Doubling recording length from 4 to 14 days yields only 2–5 percentage points of additional power for most comparisons, because between-animal variance dominates once within-animal noise has been averaged over the first several days. We therefore do not classify main-text findings into post-hoc power tiers; such a classification would be statistically coupled to realized p-values and uninformative [63].

**Table S31. Variance structure and stability summary for Tier-1 sleep metrics.**

*Mean intraclass correlation coefficient (ICC) summarizes the share of total variance attributable to between-animal differences. |Cohen's d| and minimum n per group for 80% power are derived from the strongest observed Age main effect in the Dark 1 segment (ZT12–18), n = 12 Young versus n = 12 Old, pooled across sex. ICC values computed from random-intercept linear mixed models on per-day metric values, n = 6 per Age × Sex group.*

| Metric | Mean ICC | ICC range | \|Cohen's d\| (Dark 1) | Min n for 80% power |
|---|---|---|---|---|
| NREM percentage | 0.47 | 0.17–0.83 | 2.41 | 3 |
| REM percentage | 0.32 | 0.11–0.65 | 1.24 | 11 |

| | | | | |
|---|---|---|---|---|
| NREM episode duration | 0.36 | 0.13–0.71 | 0.11 | effect near null |
| log-fragmentation index | 0.20 | 0.08–0.42 | 1.57 | 7 |

### S1.2.4 Optimal allocation: sample size, not recording duration, is the primary lever

Across the full range of baselines and cost assumptions examined, adding animals improved precision more than adding recording days, and the marginal benefit of additional days flattened rapidly after approximately the first week. At a representative baseline of n = 4 animals and 7 recording days, adding one animal reduced the standard error of the group mean by 10.6% (a structural property of the animal-count ratio that is metric-independent), while adding one recording day reduced SE by only 0.5–4.6% depending on metric and group. The ratio of marginal SE reduction from +1 animal versus +1 day ranged from 2.3× for the log-fragmentation index — the metric with the highest within-animal variance and therefore the strongest relative case for longer recordings — to 22.3× for NREM% in old males, where between-animal variance dominates. The grand mean ratio across metrics and groups was 8.1×. This asymmetry is strongest for high-ICC measures but extends qualitatively across the full metric set. Even fragmentation still benefits more from additional animals than from extending already multi-day protocols.

The practical implication is that beyond a moderate number of days, a study of this type becomes recording-heavy and animal-light relative to what its variance structure supports. For the present design (n = 6 animals, 14 days = 84 animal-days), an alternative allocation of n = 10 animals with 7 recording days (70 animal-days) would be expected to match or exceed the present design's performance for most metrics, at approximately 17% fewer total measurements. For studies aimed at detecting moderate effects (d = 0.8–1.5), n ≥ 10 per group is the more productive investment than extending recording duration. Studies targeting Age × Sex interactions specifically should plan for n = 10–12 per cell (40–48 total animals). These recommendations are summarized in Table S32.

**Table S32. Design recommendations for translational sleep–aging studies in C57BL/6J mice.**

*Conservative recommended sample sizes target detection of moderate Age effects (|d| = 1.0–1.5) at 80% power, robust across the full ICC confidence-interval range and across all four 6-h circadian segments, rather than the single largest observed effect. Minimum recording days are the smallest k for which the group-mean absolute precision falls within the metric-specific threshold (NREM% ≤ 3 percentage points, REM% ≤ 0.5 percentage points, NREM episode duration ≤ 10 s, log-fragmentation ≤ 0.15 log units). Recommendations assume balanced Age × Sex factorial designs.*

| Metric | Recommended n per | Min recording days | Notes |
|---|---|---|---|

| | group | (n=6) | |
|---|---|---|---|
| NREM percentage | 8–12 (10 typical) | 2–6 (segment-dependent) | Most statistically tractable Tier-1 endpoint |
| REM percentage | 12–18 (15 typical) | 2–4 | Sampling-limited; coarser binning recommended |
| NREM episode duration | 10–15 | 6–14 (cohort-level only) | Group-level inference; not individual-level |
| log-fragmentation index | 15–20 | 8–14 | Lowest ICC; benefits most from larger cohorts |

## S1.3 Implications

Across all metrics and configurations examined, sample size emerged as a stronger determinant of inferential precision than recording duration. For the most statistically tractable Tier-1 endpoint (NREM percentage), a cohort of n = 8–12 animals per group, recorded for approximately 7 days, provides robust detection of moderate Age effects across the full circadian cycle. Recordings exceeding approximately one week yield diminishing precision returns for most metrics, including NREM% and REM%; fragmentation-derived indices benefit somewhat more from longer recordings but still gain more from additional animals. Studies aimed at detecting Age × Sex interactions, where minimum detectable effects under the present design are large ($d \approx 1.5–2.0$), should plan for n = 10–12 per cell. These observations are offered as practical guidance derived from the present dataset and should be validated in independent or larger cohorts before adoption as design recommendations.
