# Supplementary material for: A Hierarchical and Multiscale Framework for Characterizing Mouse Sleep–Wake Dynamics from 14-Day Continuous EEG: Validation of Age- and Sex-Dependent Remodeling
Source: Cells. 2026 Jun 13;15(12):1075. doi: 10.3390/cells15121075 (PMC13296934; doi:10.3390/cells15121075)
Supplement: Supplementary file 1 [file cells-15-01075-s001.zip › Supplementary Tables.pdf]

Supplementary Table S1. Robustness of Tier 2 LMM Hour-containing interaction tests to time-factor parameterization. For each vigilance state (TDW, nTDW, NREM, REM), the Age × Time, Sex × Time, and Age × Sex × Time interactions were evaluated under three alternative representations of the 24-hour time axis: (i) a 24-level categorical Hour factor (primary analysis reported in Results §3.1, fit by random-intercept linear mixed model via ML-BFGS; inference by likelihood-ratio test, LRT, with df = 23); (ii) a two-harmonic cosinor basis (24-h fundamental + 12-h second harmonic; 4 time coefficients); (iii) a three-harmonic cosinor basis (+ 8-h third harmonic; 6 time coefficients). For the cosinor parameterizations, inference was performed via OLS with cluster-robust standard errors (Mouse as clustering variable; Cameron & Miller, 2015 [61]) to circumvent small-sample convergence difficulties of the mixed-model likelihood at these low-dimensional parameterizations; Wald F-tests on the coefficients corresponding to each interaction term provided significance tests. ΔR^2 is the marginal R^2 contribution of the interaction terms over the reduced model (reported for cosinor parameterizations only; not directly comparable to the categorical LMM's χ^2). The hourly categorical row for each state is highlighted in Panel A because it is the primary reported statistic in §3.1. Significance codes: *** p < 0.001, ** p < 0.01, * p < 0.05, † p < 0.10, ns = not significant. n = 24 mice (6 per Age × Sex cell); 576 mouse-hour observations (24 mice × 24 hours) for all models.

Panel A. Age × Time interaction. *The Age × Time interaction is robustly significant across all four states under every parameterization.*

State	Time parameterization	df	Test statistic	p	Sig	ΔR^2
**TDW**	**Hourly (24-level factor)**	23	**χ^2 = 258.08**	**< 0.0001**	***	—
	Two-harmonic cosinor (24 h + 12 h)	4,23	F = 18.57	6.1×10^{-7}	***	0.085
	Three-harmonic cosinor (+8 h)	6,23	F = 12.90	2.3×10^{-6}	***	0.097
**nTDW**	**Hourly (24-level factor)**	23	**χ^2 = 151.57**	**< 0.0001**	***	—
	Two-harmonic cosinor (24 h + 12 h)	4,23	F = 9.71	9.5×10^{-5}	***	0.060

State	Time parameterization	df	Test statistic	p	Sig	ΔR^2
	Three-harmonic cosinor (+8 h)	6,23	F = 9.37	3.0×10^{-5}	***	0.077
**NREM**	**Hourly (24-level factor)**	23	**χ^2 = 110.36**	**< 0.0001**	***	—
	Two-harmonic cosinor (24 h + 12 h)	4,23	F = 11.72	2.5×10^{-5}	***	0.032
	Three-harmonic cosinor (+8 h)	6,23	F = 8.42	6.7×10^{-5}	***	0.035
**REM**	**Hourly (24-level factor)**	23	**χ^2 = 82.88**	**< 0.0001**	***	—
	Two-harmonic cosinor (24 h + 12 h)	4,23	F = 14.42	5.0×10^{-6}	***	0.018
	Three-harmonic cosinor (+8 h)	6,23	F = 13.51	1.6×10^{-6}	***	0.020

Panel B. Sex × Time interaction. *Sex × Time attenuates under harmonic parameterization in TDW, nTDW, and NREM but remains borderline-significant to significant under the 3-harmonic basis. The hourly significance in these states is likely inflated by the DF effect.*

State	Time parameterization	df	Test statistic	p	Sig	ΔR^2
**TDW**	Hourly (24-level factor)	23	$\chi^2 = 74.39$	2.5×10^{-7}	***	—
	Two-harmonic cosinor (24 h + 12 h)	4,23	F = 2.61	0.062	†	—
	Three-harmonic cosinor (+8 h)	6,23	F = 3.17	0.020	*	—
**nTDW**	Hourly (24-level factor)	23	$\chi^2 = 38.06$	0.025	*	—
	Two-harmonic cosinor (24 h + 12 h)	4,23	F = 2.52	0.069	†	—
	Three-harmonic cosinor (+8 h)	6,23	F = 2.97	0.027	*	—
**NREM**	Hourly (24-level factor)	23	$\chi^2 = 67.28$	3.2×10^{-6}	***	—
	Two-harmonic cosinor (24 h + 12 h)	4,23	F = 2.40	0.079	†	—
	Three-harmonic cosinor (+8 h)	6,23	F = 3.76	0.009	**	—
**REM**	Hourly (24-level factor)	23	$\chi^2 = 46.65$	0.002	**	—
	Two-harmonic cosinor (24 h + 12 h)	4,23	F = 4.65	0.007	**	—

State	Time parameterization	df	Test statistic	p	Sig	ΔR²
	Three-harmonic cosinor (+8 h)	6,23	F = 3.20	0.020	*	—

Panel C. Age × Sex × Time interaction. *Non-significance of the three-way interaction is preserved across all parameterizations in all states.*

State	Time parameterization	df	Test statistic	p	Sig	ΔR²
**TDW**	Hourly (24-level factor)	23	χ^2 = 20.49	0.612	**ns**	—
	Two-harmonic cosinor (24 h + 12 h)	4,23	F = 1.64	0.198	**ns**	—
	Three-harmonic cosinor (+8 h)	6,23	F = 1.60	0.191	**ns**	—
**nTDW**	Hourly (24-level factor)	23	χ^2 = 33.20	0.078	†	—
	Two-harmonic cosinor (24 h + 12 h)	4,23	F = 0.79	0.542	**ns**	—
	Three-harmonic cosinor (+8 h)	6,23	F = 0.82	0.563	**ns**	—
**NREM**	Hourly (24-level factor)	23	χ^2 = 27.71	0.227	**ns**	—
	Two-harmonic cosinor (24 h + 12 h)	4,23	F = 1.33	0.290	**ns**	—
	Three-harmonic cosinor (+8	6,23	F = 1.48	0.230	**ns**	—

State	Time parameterization	df	Test statistic	p	Sig	ΔR²
	h)					
**REM**	Hourly (24-level factor)	23	χ^2 = 20.38	0.619	**ns**	—
	Two-harmonic cosinor (24 h + 12 h)	4,23	F = 0.81	0.533	**ns**	—
	Three-harmonic cosinor (+8 h)	6,23	F = 0.59	0.733	**ns**	—

Supplementary Table S2. Variance components and convergence diagnostics from the random-intercept LMM on 24-hour vigilance-state profiles. Model: state% ~ Age × Sex × Hour + (1 | Mouse_ID), REML estimation (Section 2.6). Var_mouse = between-mouse variance component (σ^2_between); Var_resid = residual within-mouse variance (σ^2_within); ICC = Var_mouse / (Var_mouse + Var_resid); AIC = Akaike information criterion; Cond. No. = condition number of the Hessian (values > 10^6 flag ill-conditioning). n = 6/group.

State	Var_mouse	Var_resid	ICC	AIC	Cond. No.
TDW	4.960	75.488	0.062	3822.6	1.79e+06
nTDW	16.209	36.722	0.306	3506.8	4.30e+04
NREM	8.252	92.669	0.082	3925.0	1.48e+06
REM	0.033	1.847	0.018	2029.9	1.96e+05

Supplementary Table S3. Nakagawa's marginal and conditional R^2 for the random-intercept LMM on 24-hour vigilance-state profiles. R^2 marginal = variance explained by fixed effects alone; R^2 conditional = variance explained by fixed + random effects; Gap = conditional − marginal (proportion attributable to between-mouse random intercept). n = 6/group.

State	R^2 marginal	R^2 conditional	Gap
TDW	0.772	0.786	0.014
nTDW	0.555	0.691	0.136
NREM	0.762	0.781	0.020
REM	0.807	0.811	0.003

Supplementary Table S4. Per-mouse autocorrelation diagnostics of LMM residuals on 24-hour vigilance-state profiles. ACF(1) = mean lag-1 autocorrelation of Pearson residuals across mice (negative values trimmed to 0); SD = standard deviation of ACF(1) across mice; % LB sig = percentage of mice for which the Ljung–Box test rejected independence at p < 0.05 (lags 1–12); Interp. = qualitative label (Negligible: ACF(1) < 0.1; Modest: 0.1–0.3; Substantial: > 0.3); N_eff = Bartlett's effective sample size out of 24 hourly observations per mouse; Reduction = 100% × (1 − N_eff / 24). n = 6/group.

State	ACF(1)	SD	% LB sig	Interp.	N_eff	Reduction
TDW	0.424	0.21	62%	Substantial	9.7/24	60%
nTDW	0.049	0.30	29%	Negligible	21.8/24	9%
NREM	0.201	0.20	21%	Modest	16.0/24	34%
REM	0.208	0.28	29%	Modest	15.7/24	34%

Supplementary Table S5. *Phase-level between-group and within-group paired contrasts on vigilance-state percentages, with Holm–Bonferroni correction. Between-group contrasts compare the four mouse groups pairwise within each state × phase cell (4 states × 2 phases × 4 pair types = 32 contrasts). Within-group paired contrasts compare dark vs. light phase within each group (4 states × 4 groups = 16 contrasts). Δ% = mean between-group (or within-group paired) difference with bootstrap 95% confidence interval. Cohen's d is the standardized effect size. p (raw) = uncorrected two-sample or paired t-test p-value; p (Holm) = Holm-corrected within each state × phase family of 4 between-group contrasts (4 pair types per cell) or within each state family of 4 paired dark-vs-light contrasts. Sig: * p_Holm < 0.05, ** p_Holm < 0.01, *** p_Holm < 0.001, ns = not significant. n = 6 per group.*

State	Phase	Type	Contrast	Δ% [95% CI]	Cohen's d	p (raw)	p (Holm)	Sig
TDW	Dark	Between-group	Young_Male vs Old_Male	+17.0 [+8.1, +25.9]	+2.45	0.0017	0.0051	**
TDW	Dark	Between-group	Young_Female vs Old_Female	+18.3 [+12.3, +24.3]	+3.90	0.0000	0.0000	***
TDW	Dark	Between-group	Young_Male vs Young_Female	-6.5 [-15.2, +2.2]	-0.96	0.1270	0.1970	ns
TDW	Dark	Between-group	Old_Male vs Old_Female	-5.2 [-11.5, +1.2]	-1.05	0.0985	0.1970	ns
TDW	Light	Between-group	Young_Male vs Old_Male	-0.9 [-3.7, +1.9]	-0.42	0.4792	0.9584	ns
TDW	Light	Between-	Young_Female	-0.3 [-	-0.08	0.8892	0.9584	ns

		group	vs Old_Female	4.8, +4.2]				
TDW	Light	Between-group	Young_Male vs Young_Female	-3.7 [-7.1, -0.3]	-1.40	0.0358	0.1432	ns
TDW	Light	Between-group	Old_Male vs Old_Female	-3.1 [-7.1, +1.0]	-0.96	0.1255	0.3765	ns
nTDW	Dark	Between-group	Young_Male vs Old_Male	-11.2 [-20.1, -2.2]	-1.61	0.0192	0.0576	ns
nTDW	Dark	Between-group	Young_Female vs Old_Female	-9.0 [-15.8, -2.2]	-1.71	0.0144	0.0576	ns
nTDW	Dark	Between-group	Young_Male vs Young_Female	-1.6 [-6.0, +2.8]	-0.48	0.4243	0.8486	ns
nTDW	Dark	Between-group	Old_Male vs Old_Female	+0.6 [-9.8, +10.9]	+0.07	0.9080	0.9080	ns
nTDW	Light	Between-group	Young_Male vs Old_Male	+0.8 [-3.4, +5.0]	+0.26	0.6672	1.0000	ns
nTDW	Light	Between-group	Young_Female vs Old_Female	+1.6 [-3.0, +6.3]	+0.45	0.4500	1.0000	ns
nTDW	Light	Between-group	Young_Male vs Young_Female	-2.0 [-4.6, +0.6]	-0.99	0.1179	0.4716	ns
nTDW	Light	Between-group	Old_Male vs Old_Female	-1.2 [-6.9, +4.5]	-0.27	0.6479	1.0000	ns
NREM	Dark	Between-group	Young_Male vs Old_Male	-6.1 [-14.5, +2.4]	-0.92	0.1415	0.2160	ns
NREM	Dark	Between-group	Young_Female vs Old_Female	-9.3 [-14.8, -3.8]	-2.19	0.0036	0.0144	*
NREM	Dark	Between-group	Young_Male vs Young_Female	+7.8 [-0.8, +16.4]	+1.16	0.0720	0.2160	ns
NREM	Dark	Between-	Old_Male vs	+4.5 [-	+1.13	0.0790	0.2160	ns

		group	Old_Female	0.6, +9.7]				
NREM	Light	Between-group	Young_Male vs Old_Male	-0.8 [-5.5, +3.9]	-0.21	0.7175	0.7175	ns
NREM	Light	Between-group	Young_Female vs Old_Female	-2.4 [-7.0, +2.1]	-0.68	0.2637	0.5274	ns
NREM	Light	Between-group	Young_Male vs Young_Female	+5.9 [+3.2, +8.6]	+2.82	0.0006	0.0024	**
NREM	Light	Between-group	Old_Male vs Old_Female	+4.3 [-1.7, +10.3]	+0.93	0.1397	0.4191	ns
REM	Dark	Between-group	Young_Male vs Old_Male	+0.3 [-0.7, +1.3]	+0.36	0.5508	1.0000	ns
REM	Dark	Between-group	Young_Female vs Old_Female	+0.0 [-0.7, +0.7]	+0.04	0.9443	1.0000	ns
REM	Dark	Between-group	Young_Male vs Young_Female	+0.3 [-0.5, +1.2]	+0.52	0.3908	1.0000	ns
REM	Dark	Between-group	Old_Male vs Old_Female	+0.1 [-0.8, +1.0]	+0.12	0.8394	1.0000	ns
REM	Light	Between-group	Young_Male vs Old_Male	+0.9 [+0.1, +1.7]	+1.43	0.0323	0.0969	ns
REM	Light	Between-group	Young_Female vs Old_Female	+1.1 [+0.5, +1.6]	+2.35	0.0022	0.0088	**
REM	Light	Between-group	Young_Male vs Young_Female	-0.2 [-0.8, +0.4]	-0.46	0.4444	0.8888	ns
REM	Light	Between-group	Old_Male vs Old_Female	-0.0 [-0.8, +0.7]	-0.05	0.9301	0.9301	ns
TDW	Dark-vs-Light	Within-group paired	Dark vs Light in Young_Male	+31.4 [+21.1, +41.6]	+3.21	0.0005	0.0015	**
TDW	Dark-	Within-	Dark vs Light in	+34.2	+4.86	0.0001	0.0004	***

	vs-Light	group paired	Young_Female	[+26.8, +41.5]				
TDW	Dark-vs-Light	Within-group paired	Dark vs Light in Old_Male	+13.5 [+6.1, +20.8]	+1.93	0.0052	0.0052	**
TDW	Dark-vs-Light	Within-group paired	Dark vs Light in Old_Female	+15.6 [+10.4, +20.8]	+3.15	0.0006	0.0015	**
nTDW	Dark-vs-Light	Within-group paired	Dark vs Light in Young_Male	+3.7 [+0.9, +6.5]	+1.38	0.0196	0.0392	*
nTDW	Dark-vs-Light	Within-group paired	Dark vs Light in Young_Female	+3.3 [+0.1, +6.6]	+1.07	0.0473	0.0473	*
nTDW	Dark-vs-Light	Within-group paired	Dark vs Light in Old_Male	+15.7 [+5.8, +25.7]	+1.66	0.0097	0.0291	*
nTDW	Dark-vs-Light	Within-group paired	Dark vs Light in Old_Female	+14.0 [+10.7, +17.2]	+4.54	0.0001	0.0004	***
NREM	Dark-vs-Light	Within-group paired	Dark vs Light in Young_Male	-30.2 [-38.7, -21.8]	-3.77	0.0003	0.0003	***
NREM	Dark-vs-Light	Within-group paired	Dark vs Light in Young_Female	-32.1 [-39.2, -25.0]	-4.76	0.0001	0.0003	***
NREM	Dark-vs-Light	Within-group paired	Dark vs Light in Old_Male	-25.0 [-31.0, -19.0]	-4.37	0.0001	0.0003	***
NREM	Dark-vs-Light	Within-group paired	Dark vs Light in Old_Female	-25.2 [-28.9, -21.5]	-7.12	0.0000	0.0000	***
REM	Dark-vs-Light	Within-group paired	Dark vs Light in Young_Male	-4.8 [-5.8, -3.9]	-5.41	0.0000	0.0000	***
REM	Dark-vs-Light	Within-group paired	Dark vs Light in Young_Female	-5.4 [-6.4, -4.4]	-5.62	0.0000	0.0000	***
REM	Dark-vs-Light	Within-group paired	Dark vs Light in Old_Male	-4.2 [-5.7, -2.8]	-3.05	0.0007	0.0007	***
REM	Dark-	Within-	Dark vs Light in	-4.3 [-	-11.07	0.0000	0.0000	***

vs-<br>Light	group<br>paired	Old_Female	4.7, -<br>3.9]

Supplementary Table S6. Two-harmonic cosinor parameters fitted to 24-hour vigilance-state profiles per group. Model: $Y(t) = \text{Mesor} + A_1 \cdot \cos(2\pi(t - \varphi_1)/24) + A_2 \cdot \cos(2\pi(t - \varphi_2)/12)$. Mesor = 24-hour mean (%); Amplitude = $\sqrt{(A_1^2 + A_2^2)}$ total amplitude (%); Acrophase = timing of the principal peak (ZT hour) derived from the fitted curve. Single-harmonic R^2 for TDW ≈ 0.46; two-harmonic R^2 = 0.57–0.85 (Section 2.6). See Supplementary Figure S3 for overlays. n = 6 mice per group.

State	Group	Mesor (%)	Amplitude (%)	Acrophase
TDW	Young Male	23.5	28.4	ZT 14.4
TDW	Young Female	28.6	27.1	ZT 14.3
TDW	Old Male	15.5	10.5	ZT 13.7
TDW	Old Female	19.6	12.3	ZT 22.1
nTDW	Young Male	24.9	6.3	ZT 12.0
nTDW	Young Female	26.7	4.1	ZT 12.7
nTDW	Old Male	30.0	13.1	ZT 12.3
nTDW	Old Female	30.4	9.8	ZT 14.1
NREM	Young Male	46.7	28.2	ZT 6.4
NREM	Young Female	39.8	26.6	ZT 6.1
NREM	Old Male	50.1	19.4	ZT 5.4
NREM	Old Female	45.7	16.8	ZT 5.6
REM	Young Male	4.9	4.5	ZT 6.5
REM	Young Female	4.9	4.3	ZT 6.4
REM	Old Male	4.3	3.3	ZT 5.9
REM	Old Female	4.3	3.4	ZT 6.5

Supplementary Table S7. Sensitivity analysis: significant phase-level age contrasts after excluding five mice with within-mouse CV > 0.80. Full = all 24 mice; Excl = 19 mice after exclusion (Section 2.6). Δ = between-group mean difference (%); p = independent-samples t-test (df = 10 for Full, df = 8 for Excl), uncorrected. "Robust: Yes" = contrast retains nominal significance ($p < 0.05$) after exclusion. n = 6/group (4–5 after exclusion).

State	Phase	Comparison	Δ Full	Δ Excl	p Full	p Excl	Robust
TDW	Dark	Old vs Young | M	-17.0	-14.3	0.002	0.013	Yes
TDW	Dark	Old vs Young | F	-18.3	-14.1	<0.001	0.001	Yes
NREM	Dark	Old vs Young | F	9.3	5.1	0.004	0.051	Yes
NREM	Light	F vs M | Young	-5.9	-7.6	0.001	0.006	Yes
REM	Light	Old vs Young | F	-1.1	-0.8	0.002	0.032	Yes

Supplementary Table S8. Bootstrap difference-smooth clusters from Phase-1 GAM analysis of 24-hour NREM profiles. Contiguous ZT windows in which the bootstrap 95% CI of the group-vs-group mean-difference smooth excluded zero, for each of the six pairwise contrasts (YM vs. OM, YF vs. OF, YM vs. YF, OM vs. OF, Young-pooled vs. Old-pooled, Male-pooled vs. Female-pooled). Cluster boundaries in ZT hours, durations in hours, and direction of effect are listed. Bootstrap based on 2,000 resamples of 24 mice with replacement. Cluster-level inference is exploratory (Tier-3); the Tier-1 confirmatory endpoint (TDW fraction at ZT16) is reported in Supplementary Table S10, Panels B and C, and the Tier-2 descriptive trough analysis is reported in Supplementary Table S11. n = 6/group.

Contrast	ZT Range	Duration (h)	Direction
Young vs. Old (collapsed)	ZT12.0–18.1	6.1	Young < Old
	ZT23.8–1.1	1.3	Young < Old
Male vs. Female (collapsed)	ZT18.1–22.3	4.2	Male > Female
	ZT0.9–6.3	5.4	Male > Female
	ZT8.3–9.4	1.1	Male > Female
YM vs. OM	ZT14.0–18.0	3.9	YM < OM
	ZT4.1–5.3	1.2	YM < OM
YF vs. OF	ZT12.0–16.2	4.2	YF < OF
	ZT23.7–1.1	1.4	YF < OF
YM vs. YF	ZT18.2–22.4	4.2	YM > YF
	ZT1.6–9.7	8.1	YM > YF
OM vs. OF	ZT19.8–21.8	2.0	OM > OF
	ZT3.2–5.8	2.6	OM > OF

Supplementary Table S9. Global ultradian metrics (24-hour). Values are mean ± SD. Age p: Mann–Whitney U. Age d: Cohen's d. n = 6 per group. The nominal weighted circular variance age effect (p = 0.040) is discussed in Results 3.2.2 but is not emphasized in the primary interpretation.

Metric	YM	YF	OM	OF	Age p	Age d
Total Peaks	7.67±1.37	6.83±0.98	6.83±1.33	7.83±0.75	0.759	−0.07
Total Troughs	7.33±1.51	6.67±1.03	6.67±1.86	8.17±0.98	0.275	−0.29
Wt. Circ. Var.	0.78±0.17	0.84±0.09	0.73±0.09	0.72±0.12	0.040	+0.72
Mean IPI (h)	2.77±0.49	3.22±0.57	3.42±0.97	2.62±0.41	0.862	−0.03
IPI CV	0.42±0.05	0.53±0.23	0.44±0.13	0.49±0.10	1.000	+0.05
Mean Osc. Range	26.6±1.3	31.3±5.4	32.1±6.6	29.6±3.7	0.214	−0.38

Supplementary Table S10. TDW fraction at five pre-specified fixed ZT reference points: per-group means (Panel A), Age main effect (Panel B), and Sex/interaction effects (Panel C). TDW fraction computed per mouse by pooling all 14 recording days (sum of TDW epochs / sum of total wake epochs within the 15-min bin centered on each reference point). Factorial Age × Sex ANOVA on per-mouse 14-day-averaged values (OLS, Type II sum of squares; n = 24; 6 per cell). Hedges' g reflects the pooled Age main effect (Young n = 12 vs Old n = 12) with small-sample correction. ZT16 is the Tier 1 primary confirmatory endpoint (highlighted); the remaining four points are supporting/sensitivity analyses. Group values are mean ± SD of per-mouse TDW fractions. All F values are df = 1, 20. Positive d and g indicate Young > Old; negative values indicate Old > Young. See Supplementary Figure S12 for visual summary.

Panel A. *TDW fraction by group (mean ± SD) at each fixed ZT reference point.*

ZT point	YM — Young Male	YF — Young Female	OM — Old Male	OF — Old Female
ZT16 (Tier 1)	0.672 ± 0.058	0.584 ± 0.096	0.387 ± 0.157	0.396 ± 0.112
ZT20	0.351 ± 0.113	0.435 ± 0.156	0.292 ± 0.129	0.347 ± 0.134
ZT23.5	0.703 ± 0.107	0.586 ± 0.120	0.511 ± 0.174	0.555 ± 0.133
ZT0.5	0.453 ± 0.205	0.499 ± 0.141	0.407 ± 0.076	0.467 ± 0.151
ZT3	0.242 ± 0.095	0.269 ± 0.090	0.339 ± 0.082	0.348 ± 0.083

Panel B. ***Age main effect: F-statistic, uncorrected p-value, and effect sizes (Cohen's d, Hedges' g) for the pooled Young vs Old contrast.***

ZT point	F(Age)	p(Age)	Cohen's d	Hedges' g
ZT16 (Tier 1)	26.94	<0.0001	+2.12	+2.05
ZT20	1.82	0.192	+0.55	+0.54

ZT point	F(Age)	p(Age)	Cohen's d	Hedges' g
ZT23.5	4.02	0.059	+0.81	+0.78
ZT0.5	0.40	0.536	+0.26	+0.26
ZT3	6.01	0.024	−1.04	−1.01

Panel C. ***Sex main effect and Age × Sex interaction: F-statistics and uncorrected p-values from the 2 × 2 factorial ANOVA.***

ZT point	F(Sex)	p(Sex)	F(Age × Sex)	p(Age × Sex)
ZT16 (Tier 1)	0.76	0.392	1.12	0.303
ZT20	1.62	0.217	0.07	0.793
ZT23.5	0.43	0.521	2.10	0.163
ZT0.5	0.73	0.403	0.01	0.913
ZT3	0.26	0.618	0.07	0.798

Supplementary Table S11. Tier-2 descriptive test — factorial Age × Sex ANOVA on TDW fraction at the data-driven trough (Section 3.2.3). Type II sum of squares, ordinary least squares. Columns: SS = sum of squares; df = degrees of freedom; F = F-statistic; p = uncorrected p-value; partial η^2 = SS_effect / (SS_effect + SS_residual). Age, Sex, and Age × Sex effects are reported; this Tier-2 descriptive analysis complements the Tier-1 primary confirmatory endpoint at the pre-specified ZT16 time point, which is reported in Supplementary Table S10 (ZT16 row) and Section 3.2.3. n = 24 (6 per Age × Sex cell).

Effect	F	df	p
Age (main effect)	12.58	1, 20	0.002

Sex (main effect)	0.40	1, 20	0.533
Age × Sex interaction	2.17	1, 20	0.156

Supplementary Table S12. Exploratory N-shape landmarks (Tier 3). † Trough ZT values are reported as circular mean ± circular SD (in ZT clock hours on the 24-hour cycle); hypothesis tests for trough ZT were performed on the linear representation "hours after ZT20" (values in [0, 6] h, where 0 corresponds to ZT20 and 6 corresponds to ZT2). All other metrics are mean ± SD. n = 6 per group.

Metric	YM	YF	OM	OF	Age p	Age d	Sex p	Sex d
Trough ZT †	ZT23.75±0.29	ZT22.29±0.42	ZT23.05±0.91	ZT22.21±0.64	0.323	+0.45	0.001	+1.66
Peak 2 Amp (%)	77.12±3.61	72.31±5.48	82.08±4.45	75.14±5.30	0.069	−0.71	0.026	+1.17
Pk2/Pk1 Ratio	1.13±0.09	1.27±0.26	1.23±0.15	1.28±0.26	0.260	−0.29	0.371	−0.50
Asymmetry	0.98±0.63	1.70±0.74	0.95±0.81	1.07±0.24	0.429	+0.48	0.106	−0.71
NREM–TDW corr	−0.94±0.02	−0.95±0.02	−0.86±0.10	−0.89±0.03	<0.001	−1.22	0.840	+0.32

Supplementary Table S13. Binomial GLM on wake microcomposition at the N-shape trough. Endpoint: TDW counts out of total wake counts per 15-min bin (90 epochs). Logit link, Age × Sex fixed effects. OR = odds ratio.

Effect	Log-odds	Odds Ratio	SE	p
Intercept (YM reference)	+1.152	3.17	0.104	<0.0001
Age [old]	−1.148	0.32	0.137	<0.0001
Sex [female]	−0.519	0.60	0.142	0.0003
Age × Sex interaction	+0.710	2.03	0.191	0.0002

Supplementary Table S14. ICC values for primary landmarks across preprocessing variants. ICC(2,1); computed across nine combinations of Savitzky–Golay window (3, 5, 7 bins) × peak prominence threshold (8%, 10%, 12%). Interpretation: <0.50 = poor; 0.50–0.75 = moderate; 0.75–0.90 = good; >0.90 = excellent.

Landmark	ICC (2,1)	Interpretation
TDW Frac. at Trough	0.913	Excellent
Peak 1 Amplitude	0.855	Good
Time to Peak 1	0.718	Moderate

FWHM	0.485	Poor
Peak 1 ZT	0.296	Poor

Supplementary Table S15. Day-level validation: mixed-effects models on daily TDW fraction. Two models confirm that the primary age-related TDW fraction reduction is detectable at the day level rather than only after 14-day averaging. Panel A reports the Tier 1 primary confirmatory endpoint (ZT16 TDW fraction); Panel B reports the Tier 2 descriptive endpoint (data-driven trough TDW fraction). *Panel A — Primary model:* ZT16_TDW_frac ~ Age × Sex + (1 | Mouse), n = 320 animal-days (24 mice × 14 days = 336, minus 16 animal-days excluded due to zero wake epochs in the ZT16 15-min bin). *Panel B — Secondary model:* trough_TDW_frac ~ Age × Sex + (1 | Mouse), n = 336 animal-days. Both models: REML estimation; LBFGS optimizer; Mouse_ID as random intercept. The Day drift coefficient is estimated in a separate additive-Day model fit independently for each endpoint and is reported in the Day row of each panel; it tests whether daily TDW fraction at the relevant timepoint shows a progressive linear trend across the 14-day recording window. Animal ICC quantifies the proportion of total variance attributable to between-mouse differences in this single day-of-recording window. Reference category for fixed effects: YM (Young Male). n = 6 mice per Age × Sex cell.*Panel A. Tier-1 primary endpoint: ZT16 TDW fraction (n = 320 animal-days; 16 excluded for zero wake)*

Effect / Parameter	Estimate	SE	z	p
Intercept (YM reference)	+0.622	0.043	+14.60	<0.001
Age [old]	−0.229	0.060	−3.78	<0.001
Sex [female]	−0.089	0.061	−1.46	0.145
Age × Sex interaction	+0.062	0.086	+0.72	0.470
Day (drift, separate model)	−0.0016	0.004	−0.41	0.680
Animal ICC	0.063	—	—	—

Panel B. Tier-2 descriptive endpoint: data-driven trough TDW fraction (n = 336 animal-days)

Effect / Parameter	Estimate	SE	z	p
Intercept (YM reference)	+0.765	0.060	+12.71	<0.001
Age [old]	−0.200	0.085	−2.35	0.019
Sex [female]	−0.147	0.085	−1.72	0.085
Age × Sex interaction	+0.221	0.120	+1.83	0.067
Day (drift, separate model)	−0.0004	0.003	−0.16	0.873
Animal ICC	0.318	—	—	—

Supplementary Table S16. Bi-exponential mixture decomposition of TDW bout-length distributions (Section 3.3.5). Per-mouse bout-length distributions were fit with $f(t) = \pi \cdot \lambda\_fast \cdot \exp(-\lambda\_fast \cdot t) + (1 - \pi) \cdot \lambda\_slow \cdot \exp(-\lambda\_slow \cdot t)$. Columns: π = mixing fraction (short-bout component); λ_fast = rate of fast (short-bout) exponential; λ_slow = rate of slow (long-bout) exponential; mean bout duration components = 1/λ. Age p = independent-samples t-test (df = 10) on per-mouse parameter estimates; bold rows: Age p < 0.05. n = 6/group.

**Parameter**	**Phase**	**YM**	**YF**	**OM**	**OF**	**p Age**	**η^2**

TDW π_short	Dark	0.40±0.04	0.33±0.05	0.52±0.03	0.51±0.04	**.011**	.28
TDW τ_short (s)	Dark	125±48	181±52	47±4	51±5	**.008**	.29
TDW τ_long (s)	Dark	360±30	342±49	245±41	272±23	**.020**	.24
TDW π_short	Light	0.78±0.02	0.54±0.06	0.82±0.02	0.76±0.02	**.004**	.18
nTDW π_short	Dark	0.41±0.02	0.47±0.03	0.31±0.02	0.34±0.02	**<.001**	.42
nTDW τ_long (s)	Dark	157±5	164±15	215±27	208±15	**.009**	.30

Supplementary Table S17. NREM→Wake negative binomial GLMM (Model A1). Panel A: fixed-effect coefficient estimates on the log scale (conditional model). Panel B: Type III Wald χ^2 tests for each model term. The model included Age × Sex × Phase fixed effects with a log(NREM-epochs) offset and random intercepts for Mouse and Mouse:Day; reference levels are Age = Young, Sex = Male, Phase = Light. Planned between-group contrasts derived from this model are reported in Supplementary Table S18, and model selection against Poisson and zero-inflated alternatives is reported in Supplementary Table S20.

Panel A. Fixed-effect coefficient estimates (conditional model).

Term	Estimate	SE	z	p
(Intercept)	−4.7585	0.1008	−47.20	< 2e−16***
AgeOld	0.3649	0.1420	2.57	0.0101*
SexFemale	−0.4032	0.1439	−2.80	0.0051**
PhaseDark	0.3572	0.0428	8.35	< 2e−16***
AgeOld:SexFemale	0.3936	0.2018	1.95	0.0511.
AgeOld:PhaseDark	0.1982	0.0550	3.60	0.0003***
SexFemale:PhaseDark	0.4603	0.0663	6.94	3.84e−12***
AgeOld:SexFemale:PhaseDark	−0.4891	0.0831	−5.88	4.02e−9***

Panel B. Type III Wald χ^2 tests.

Effect	χ^2	df	p
(Intercept)	2228.13	1	< 2.2e−16***
Age	6.61	1	0.0101*
Sex	7.86	1	0.0051**
Phase	69.70	1	< 2.2e−16***
Age × Sex	3.80	1	0.0511.
Age × Phase	12.99	1	0.0003***

Sex × Phase	48.20	1	3.84e−12***
Age × Sex × Phase	34.61	1	4.02e−9***

Family: nbinom2 (log link). Dispersion parameter: 5.19. Random effects: Mouse variance = 0.0558, Mouse:Day variance = 0.0097. n = 7,319 observations from 24 mice across 336 mouse-days. AIC = 28,210.9. Overdispersion ratio (Pearson): 1.574. Reference levels: Age = Young, Sex = Male, Phase = Light. Model specification: NREM_Wake ~ Age × Sex × Phase + offset(log(NREM_epochs)) + (1 | Mouse) + (1 | Mouse:Day). Significance codes: *** p < 0.001, ** p < 0.01, * p < 0.05, . p < 0.1.

Supplementary Table S18. NREM→Wake planned contrasts (EMMs, rate ratios). Key results: Old/Young females light phase RR = 2.11 [1.58, 2.83], $p < 0.0001$; Old/Young males light phase RR = 1.45 [1.07, 1.96], $p = 0.016$; Old/Young males dark phase RR = 1.73, $p = 0.0005$; Old/Young females dark phase RR = 1.67, $p = 0.002$. Sex contrast in young light phase: Female/Male RR = 0.67, $p = 0.008$; abolished in old: RR = 0.98, $p = 0.89$.

Contrast	RR	95% CI	z	p
Old/Young Male Dark	1.73	[1.27, 2.36]	3.49	0.0005***
Old/Young Male Light	1.45	[1.07, 1.96]	2.40	0.0163*
Old/Young Female Dark	1.67	[1.21, 2.31]	3.12	0.0018**
Old/Young Female Light	2.11	[1.58, 2.83]	5.06	<0.0001***
Female/Male Young Dark	1.01	[0.71, 1.43]	0.05	0.9618
Female/Male Young Light	0.67	[0.50, 0.90]	-2.66	0.0079**
Female/Male Old Dark	0.97	[0.73, 1.29]	-0.18	0.8536
Female/Male Old Light	0.98	[0.73, 1.32]	-0.14	0.8875

Supplementary Table S19. NREM→REM NB GLMM fixed-effect estimates. Main effects: Age $\chi^2(1) = 7.30$, $p = 0.007$; Sex $\chi^2(1) = 8.72$, $p = 0.003$; Phase $\chi^2(1) = 105.0$, $p < 0.0001$. Age × Phase interaction $\chi^2(1) = 14.2$, $p = 0.0002$. Three-way interaction $p = 0.54$; Age × Sex $p = 0.91$. Planned contrasts: Old/Young dark-phase RR = 0.71 (males), 0.67 (females); light-phase RR = 0.85, 0.84. Female/Male RR = 1.17–1.23 across conditions (all $p < 0.03$).

Fixed effect	β	SE	z	p
Intercept	-4.451	0.080	-55.55	<0.0001***
Age	-0.350	0.157	-2.23	0.0256*
Sex	0.192	0.091	2.10	0.0358*
Phase	0.331	0.037	9.00	<0.0001***
Age × Sex	-0.035	0.178	-0.20	0.8427

Age × Phase	0.174	0.140	1.24	0.2152
Sex × Phase	-0.018	0.056	-0.32	0.7486
Age × Sex × Phase	0.037	0.158	0.23	0.8152

Supplementary Table S20. Model comparison for NREM-exit transition models (Section 2.9). Candidate generalized linear mixed models were fit to hourly counts of NREM→Wake (models A0–A2) and NREM→REM (models B0–B1) transitions, each including Age × Sex × Phase fixed effects, a log(NREM-epochs) offset, and random intercepts for Mouse and Mouse:Day; a binomial REM-gating model (B2) is included for reference. AIC is reported for each model. For NREM→Wake, the negative binomial model (A1) was selected as the primary model for inference; the negative binomial cosinor model (A2) attained a lower AIC and is retained for descriptive characterization of circadian structure (Section 3.4.1). For NREM→REM, the negative binomial model (B0) was selected as primary; the zero-inflated negative binomial model (B1) was evaluated but not adopted because its zero-inflation component was non-significant and AIC favored the simpler model. n = 6/group, 14 recording days per mouse.

Model	Outcome	Family	AIC	Note
**A0**	NREM→Wake	Poisson	28702	Overdispersed
**A1**	NREM→Wake	NB	28211	Primary
**A2**	NREM→Wake (cosinor)	NB	28103	Circadian (descriptive)
**B0**	NREM→REM	NB	22678	Primary
**B1**	NREM→REM	ZINB	22686	ZI rejected
**B2**	REM gating	Binomial	6123	Supplement

Supplementary Table S21. Per-mouse cosinor parameters for ultradian block architecture (Section 3.5). Cosinor model $Y(t) = M + A \cdot \cos(2\pi(t - \varphi)/24)$ fitted to each mouse's 3-h-binned time series of block duration, wake fraction, NREM fraction, and REM fraction. Columns: MESOR = 24-hour mean; Amplitude = A (in native units of the metric); Acrophase = φ (ZT hour). Per-mouse estimates tested in Supplementary Table S22 via Age × Sex ANOVA. n = 6/group.

Metric	Group	MESOR	Amplitude	Acrophase (h)
Duration (s)	YM	2931±141	1468±131	14.8±0.5
Duration (s)	YF	3469±170	1358±91	14.8±0.2
Duration (s)	OM	2883±62	1670±247	16.3±0.2
Duration (s)	OF	2696±231	1508±124	16.7±0.4
Wake Frac.	YM	0.046±0.004	0.013±0.003	17.1±1.5

Wake Frac.	YF	0.037±0.006	0.015±0.003	9.5±3.1
Wake Frac.	OM	0.065±0.005	0.018±0.003	9.5±2.9
Wake Frac.	OF	0.062±0.005	0.015±0.003	17.6±1.3
REM Frac.	YM	0.079±0.003	0.027±0.002	16.4±0.5
REM Frac.	YF	0.094±0.002	0.025±0.002	17.0±0.5
REM Frac.	OM	0.063±0.003	0.027±0.004	17.2±0.6
REM Frac.	OF	0.069±0.003	0.028±0.001	18.5±0.2

Supplementary Table S22. Age × Sex ANOVA on per-mouse cosinor parameters of ultradian block architecture; significant effects only. Per-mouse MESOR / Amplitude / Acrophase estimates from Supplementary Table S21 entered into a factorial Age × Sex ANOVA per metric × parameter combination. Columns: F-statistic, uncorrected p-value. Non-significant effects (p ≥ 0.05) are omitted. n = 24 (6 per Age × Sex cell).

**Metric**	**Parameter**	**Effect**	**F**	**p**
Duration	MESOR	Age	7.17	0.015*
Duration	MESOR	Age×Sex	5.66	0.027*
Duration	Acrophase	Age	20.24	<0.001***
Wake Frac.	MESOR	Age	21.11	<0.001***
Wake Frac.	Acrophase	Age×Sex	11.06	0.003**
NREM Frac.	Amplitude	Age×Sex	6.30	0.021*
REM Frac.	MESOR	Age	53.45	<0.001***
REM Frac.	MESOR	Sex	13.66	0.001**
REM Frac.	Acrophase	Age	5.66	0.027*

Supplementary Table S23. Poisson GEE rate ratios for ultradian-block fragmentation, adjusted for total sleep duration (Section 2.10, Analysis 2). Endpoint: number of intra-block NREM→Wake transitions per 6-h period. Model: Poisson GEE with Age, Sex, and their interactions with circadian 6-h period (ED/LD/EL/LL = early-dark/late-dark/early-light/late-light), plus centered total-sleep-duration covariate, exchangeable working correlation. RR = exp(β); columns: RR, 95% CI, z-statistic, uncorrected p. n = 6/group.

**Effect**	**RR**	**95% CI**	**z**	**p**

Age (Old vs Young)	1.29	[0.92, 1.79]	1.48	0.138
Sex (Female vs Male)	0.83	[0.63, 1.08]	-1.39	0.163
Age×Sex	1.28	[1.05, 1.55]	2.40	0.016*
Age×Period (EL)	0.73	[0.56, 0.96]	-2.24	0.025*
Age×Period (LL)	0.61	[0.46, 0.81]	-3.44	<0.001**

Supplementary Table S24. Out-block hurdle model (Analysis 4).

Effect	OR / RR	95% CI	z	p
Part A — Logistic GEE (probability of ≥1 out-block per bin)				
**Age (Old vs Young)**	**2.72**	**[1.27, 5.82]**	**2.58**	**0.010***
**Sex (Female vs Male)**	**0.80**	**[0.29, 2.25]**	**−0.42**	**0.678**
**Age × Sex**	**0.80**	**[0.30, 2.15]**	**−0.44**	**0.661**
Part B — Poisson GEE (out-block count conditional on ≥1 present)				
**Age (Old vs Young)**	**1.37**	**[1.16, 1.62]**	**3.73**	**<0.001*** **
**Sex (Female vs Male)**	**1.14**	**[0.91, 1.43]**	**1.14**	**0.255**
**Age × Sex**	**0.96**	**[0.70, 1.32]**	**−0.25**	**0.803**

Part A: Logistic GEE on presence (≥1) of out-blocks per 3-h circadian bin. Part B: Poisson GEE on out-block count conditional on ≥1 present in the bin. Both models include Age, Sex, and Age × Sex as fixed effects, with exchangeable working correlation clustered by mouse. OR = odds ratio (Part A); RR = rate ratio (Part B). Reference categories: Young, Male. Part A: 24 mice × 14 days × 8 bins = 2,688 bin-observations; Part B: restricted to n = 1,462 bins with ≥1 out-block. *p < 0.05, **p < 0.01, ***p < 0.001.

Note. Out-blocks defined per Methods §2.10: single-state blocks (containing only NREM or only REM, without NREM↔REM transition), duration < 500 s (< 50 epochs), separated from nearest neighboring sleep episode by > 180 s (> 18 wake epochs) on both sides. Hurdle model decomposes out-block occurrence into two conditionally independent processes: the presence of any out-block per 3-h bin (Part A) and the count of out-blocks given that at least one is present (Part B). Total out-block episodes identified across all recordings: 2,828.

Supplementary Table S25. Summary of significant CBPT clusters on absolute PSD.

State	Phase	Effect	Freq Range (Hz)	N bins	Cluster Mass	p_FWER	Sig
TDW	dark	Age	2.8–7.6	49	169.4	0.041	*
TDW	dark	Age×Sex	1.0–9.5	86	291.7	0.009	**
TDW	dark	Age×Sex	11.7–20.0	84	213.6	0.024	*
TDW	light	Age	2.1–7.1	51	170.7	0.036	*
TDW	light	Age×Sex	1.0–9.1	82	283.3	0.015	*
TDW	light	Age×Sex	11.2–20.0	89	217.7	0.028	*
nTDW	dark	Age×Sex	1.1–20.0	190	557.5	0.006	**
nTDW	light	Age×Sex	1.0–20.0	191	572.8	0.005	**
NREM	dark	Age	10.2–20.0	99	−275.5	0.028	*
NREM	dark	Age×Sex	3.7–20.0	164	518.2	0.007	**
NREM	light	Age	11.9–20.0	82	−217.1	0.044	*
NREM	light	Age×Sex	3.3–20.0	168	501.5	0.010	**
REM	dark	Age×Sex	3.5–7.9	45	129.7	0.036	*
REM	dark	Age×Sex	9.6–15.5	60	151.1	0.028	*
REM	light	Age×Sex	2.8–20.0	173	448.1	0.008	**

**p < 0.05, **p < 0.01. Cluster mass = sum of t-statistics across bins exceeding |t| > 2.086. Significance assessed against 5,000 permutation null distribution with FWER control.*

Supplementary Table S26. *Cells appearing in the top 20 by η^2p (Age effect) in two or more normalization frameworks. Dash indicates the cell was not in that normalization's top 20.*

State	Hour (ZT)	Band (Hz)	η^2p (24h)	η^2p (Phase)	η^2p (ZT8–11)	Convergence	CBPT overlap
TDW	1 (ZT12)	10–11	0.670	0.572	0.525	All three	Age×Sex cluster
TDW	1 (ZT12)	11–12	0.703	0.671	0.591	All three	Age×Sex cluster
TDW	1	12–13	0.610	0.620	0.500	All three	Age×Sex

	(ZT12)						cluster
nTDW	2 (ZT13)	1–2	0.653	0.485	0.525	All three	Age×Sex cluster
TDW	1 (ZT12)	7–8	0.626	—	0.609	24h + ZT8–11	Age cluster
TDW	3 (ZT14)	7–8	0.607	—	0.559	24h + ZT8–11	Age cluster
TDW	6 (ZT17)	2–3	0.491	—	0.619	24h + ZT8–11	—
REM	11 (ZT22)	5–6	0.585	—	0.571	24h + ZT8–11	Age×Sex cluster
REM	12 (ZT23)	18–19	0.565	—	0.578	24h + ZT8–11	Age×Sex cluster
TDW	1 (ZT12)	13–14	0.629	0.535	—	24h + Phase	Age×Sex cluster
NREM	12 (ZT23)	3–4	0.508	0.567	—	24h + Phase	—
NREM	13 (ZT0)	1–2	—	0.506	0.490	Phase + ZT8–11	—

Supplementary Table S27. The 20 hour × band × state cells with the largest partial η^2p for the Age effect. F-statistics from 2 × 2 ANOVA on 24h-normalized $\log_{10}$ spectral power. p(FDR) = Benjamini–Hochberg corrected (family = 456 tests per state × effect). Group means are normalized values (0 = 24-hour reference level).

State	Hour	ZT	Band	Phase	F-stat	p-value	p(FDR)	η^2p	YM	OM	YF
TDW	1	12	11-12	dark	47.38	1.09e-6	0.0005	0.703	0.048	-0.000	0.049
TDW	1	12	10-11	dark	40.61	3.23e-6	0.0007	0.670	0.059	0.002	0.053
nTDW	2	13	1-2	dark	37.60	5.43e-6	0.0025	0.653	-0.008	-0.032	0.002
TDW	1	12	13-14	dark	33.91	1.07e-5	0.0013	0.629	0.026	-0.000	0.020
TDW	1	12	7-8	dark	33.43	1.17e-5	0.0013	0.626	-0.038	-0.001	-0.036
TDW	1	12	12-	dark	31.3	1.77e-	0.0015	0.61	0.03	-	0.03

			13		3	5		0	9	0.003	0
TDW	3	14	7-8	dark	30.86	1.95e-5	0.0015	0.607	-0.049	0.011	-0.034
TDW	11	22	13-14	dark	29.90	2.37e-5	0.0015	0.599	0.015	-0.009	0.006
REM	11	22	5-6	dark	12.67	0.0061	0.147	0.585	-0.020	-0.088	-0.021
REM	22	9	15-16	light	26.10	5.35e-5	0.024	0.566	-0.003	-0.015	0.012
REM	12	23	18-19	dark	11.69	0.0076	0.147	0.565	-0.035	0.005	0.003
REM	11	22	4-5	dark	10.53	0.0101	0.177	0.539	0.008	-0.020	-0.016
TDW	1	12	6-7	dark	23.22	0.0001	0.006	0.537	-0.048	-0.019	-0.038
TDW	11	22	8-9	dark	22.77	0.0001	0.006	0.532	0.011	0.033	0.003
NREM	12	23	3-4	dark	15.49	0.0013	0.121	0.508	-0.045	0.015	-0.012
REM	24	11	9-10	light	20.61	0.0002	0.045	0.508	0.025	-0.012	0.020
TDW	2	13	11-12	dark	19.61	0.0003	0.012	0.495	0.036	0.012	0.051
TDW	6	17	2-3	dark	18.32	0.0004	0.013	0.491	-0.010	-0.033	-0.011
NREM	21	8	1-2	light	18.94	0.0003	0.121	0.486	-0.007	0.017	-0.023
TDW	17	4	11-12	light	17.84	0.0005	0.014	0.484	-0.034	0.007	-0.029

Supplementary Table S28. Scalar band-power linear-model results (Section 3.6.3); only FDR-significant effects shown (q_FDR < 0.05). Six features analyzed: NREM delta (1–4 Hz, $\log_{10}$), NREM sigma (10–15 Hz, $\log_{10}$), TDW theta (5–9 Hz, $\log_{10}$), REM theta (5–9 Hz, $\log_{10}$), TDW theta-peak centroid frequency, REM theta-peak centroid frequency — each per dark and light phase. Linear models with Age, Sex, and Age × Sex terms; BH-FDR correction across 36 tests (6 features × 2 phases × 3 effects). Columns: β = fixed-effect estimate, SE = standard error, t = t-statistic, p = uncorrected, q_FDR = BH-corrected, Sig = *q < 0.05, **q < 0.01, ***q < 0.001. n = 6/group.

Feature	Phase	Effect	β	SE	t	p	q_FDR	Sig
NREM delta	dark	Age	−0.159	0.047	−3.38	7.3×10^{-4}	0.004	**
NREM delta	light	Age	−0.122	0.043	−2.82	0.005	0.014	*
NREM sigma	dark	Age	−0.151	0.035	−4.35	1.4×10^{-5}	<0.001	***
NREM sigma	dark	Age×Sex	0.173	0.049	3.53	4.1×10^{-4}	<0.001	***
NREM sigma	light	Age	−0.147	0.023	−6.31	$<10^{-9}$	<0.001	***
NREM sigma	light	Age×Sex	0.174	0.048	3.64	2.8×10^{-4}	<0.001	***
TDW theta	dark	Sex	−0.120	0.047	−2.59	0.010	0.012	*
TDW theta	dark	Age×Sex	0.255	0.071	3.61	3.1×10^{-4}	<0.001	***
TDW theta	light	Sex	−0.119	0.029	−4.05	5.2×10^{-5}	<0.001	***
TDW theta	light	Age×Sex	0.255	0.061	4.21	2.6×10^{-5}	<0.001	***
REM theta	dark	Sex	−0.104	0.032	−3.22	0.001	0.002	**
REM theta	dark	Age×Sex	0.245	0.067	3.68	2.3×10^{-4}	<0.001	***
REM theta	light	Sex	−0.095	0.023	−4.24	2.2×10^{-5}	<0.001	***
REM theta	light	Age×Sex	0.240	0.065	3.71	2.1×10^{-4}	<0.001	***
TDW centroid	dark	Age	−0.135	0.021	−6.32	$<10^{-9}$	<0.001	***
TDW centroid	light	Age	−0.134	0.029	−4.60	4.0×10^{-6}	<0.001	***

Supplementary Table S29. FOOOF spectral-parameterization linear-model results (Section 3.6.3); only FDR-significant effects shown (q_FDR < 0.05). FOOOF (specparam v2.0.0rc6) decomposition of absolute PSD over 1–20 Hz (fixed aperiodic mode, no knee) per mouse × state × phase. Parameters: Offset (aperiodic intercept), Exponent (aperiodic slope), Theta CF (peak center frequency, 5–9 Hz), Theta PW (peak power), Theta BW (peak bandwidth). Linear models with Age, Sex, and Age × Sex terms fit separately for each state × parameter combination; BH-FDR correction across aperiodic and periodic families separately. Columns: β, SE, t, p (uncorrected), q_FDR, Sig encoding as in Supplementary Table S28. Mean model $R^2 = 0.977 \pm 0.019$. n = 6/group.

Parameter	State	Effect	β	SE	t	p	q_FDR	Sig
Offset	TDW	Age×Sex	0.203	0.054	3.79	1.5×10^{-4}	0.004	**
Theta CF	TDW	Age	−0.781	0.213	−3.67	2.4×10^{-4}	0.003	**
Theta CF	nTDW	Age	−0.892	0.113	−7.91	$<10^{-9}$	<0.001	***

Theta CF	NREM	Age	−0.406	0.150	−2.70	0.007	0.042	*
Theta PW	NREM	Age	0.055	0.015	3.76	1.7×10^{-4}	0.003	**
Theta PW	nTDW	Age×Sex	0.071	0.026	2.75	0.006	0.042	*
Theta PW	REM	Age×Sex	0.125	0.046	2.74	0.006	0.042	*
Theta BW	NREM	Age	−0.473	0.180	−2.64	0.008	0.043	*

Supplementary Table S30. Sensitivity of the Age effect on out-block count to block-identification threshold choice. Eight threshold settings were tested: the baseline (highlighted) plus one-at-a-time variants of three parameters — wake-bridging duration (2, 4, 5 min vs baseline 3 min), out-block minimum block length (300, 700 s vs baseline 500 s), and out-block isolation criterion (120, 240 s vs baseline 180 s). At each setting, single-state blocks were classified as out-blocks if their duration was below the minimum length AND their left and right wake gaps both exceeded the isolation criterion. The Age rate ratio (RR) is the exponentiated Age coefficient from a negative-binomial GEE with Age + Sex fixed effects and Mouse clustering (exchangeable working correlation), fit to per-(mouse, day, 3-h bin) out-block counts conditional on at least one out-block in that bin. The Age RR is preserved in direction (Old > Young) across all eight settings and remains statistically significant ($p < 0.05$) in six of eight, with one near-significant setting (bridge = 5 min, $p = 0.073$) and one non-significant setting (min_len = 300 s, $p = 0.138$). Sig: ** $p < 0.01$, * $p < 0.05$, † $p < 0.10$, ns = not significant. See Supplementary Figure S18 (block-threshold justification) for the empirical distribution underlying these threshold choices.

Threshold setting	Bridge (min)	Min length (s)	Iso (s)	N valid blocks	N out-blocks	Age RR (count)	p	Sig
bridge = 2 min	2	500	180	6,927	2,770	1.33	0.041	*
**baseline**	3	500	180	5,917	2,809	**1.32**	0.048	*
bridge = 4 min	4	500	180	5,652	2,371	1.32	0.049	*
bridge = 5 min	5	500	180	5,451	2,023	1.29	0.073	†
min_len = 300 s	3	300	180	6,431	2,295	1.23	0.138	**ns**

Threshold setting	Bridge (min)	Min length (s)	Iso (s)	N valid blocks	N out-blocks	Age RR (count)	p	Sig
min_len = 700 s	3	700	180	5,513	3,213	1.43	0.012	*
iso = 120 s	3	500	120	5,908	2,818	1.32	0.048	*
iso = 240 s	3	500	240	6,386	2,340	1.32	0.046	*

Supplementary Table S31. Variance structure and stability summary for Tier-1 sleep metrics. Mean intraclass correlation coefficient (ICC) summarizes the share of total variance attributable to between-animal differences. |Cohen's d| and minimum n per group for 80% power are derived from the strongest observed Age main effect in the Dark 1 segment (ZT12–18), n = 12 Young versus n = 12 Old, pooled across sex. ICC values computed from random-intercept linear mixed models on per-day metric values, n = 6 per Age × Sex group.

Metric	Mean ICC	ICC range	|Cohen's d| (Dark 1)	Min n for 80% power
NREM percentage	0.47	0.17–0.83	2.41	3
REM percentage	0.32	0.11–0.65	1.24	11
NREM episode duration	0.36	0.13–0.71	0.11	effect near null
log-fragmentation index	0.20	0.08–0.42	1.57	7

Supplementary Table S32. Design recommendations for translational sleep–aging studies in C57BL/6J mice. Conservative recommended sample sizes target detection of moderate Age effects (|d| = 1.0–1.5) at 80% power, robust across the full ICC confidence-

interval range and across all four 6-h circadian segments, rather than the single largest observed effect. Minimum recording days are the smallest k for which the group-mean absolute precision falls within the metric-specific threshold (NREM% ≤ 3 percentage points, REM% ≤ 0.5 percentage points, NREM episode duration ≤ 10 s, log-fragmentation ≤ 0.15 log units). Recommendations assume balanced Age × Sex factorial designs.

Metric	Recommended n per group	Min recording days (n=6)	Notes
NREM percentage	8–12 (10 typical)	2–6 (segment-dependent)	Most statistically tractable Tier-1 endpoint
REM percentage	12–18 (15 typical)	2–4	Sampling-limited; coarser binning recommended
NREM episode duration	10–15	6–14 (cohort-level only)	Group-level inference; not individual-level
log-fragmentation index	15–20	8–14	Lowest ICC; benefits most from larger cohorts
